# Modular Assembled Targeting Chimera Enables Multimodal Targeted Protein Degradation

**DOI:** 10.1021/jacsau.5c01131

**Published:** 2025-11-05

**Authors:** Wentao Zhu, Wenqian Zhang, Yinmiao Wang, Jian Chen, Fang Xu, Jiyan Pang

**Affiliations:** † School of Chemistry, 26469 Sun Yat-sen University, Guangzhou 510006, China; ‡ International Cooperative Laboratory of Traditional Chinese Medicine Modernization & Innovative Drug Development of Chinese Ministry of Education (MOE) & Guangzhou City Key Laboratory of Precision Chemical Drug Development, School of Pharmacy, 47885 Jinan University, Guangzhou 510632, China

**Keywords:** targeted protein degradation, MDA-MB-231, self-assembly, antitumor

## Abstract

Targeted protein degradation (TPD) has emerged as a promising therapeutic strategy, with advantages over traditional protein inhibition. Despite significant advancements in novel degraders such as bivalents and multitargeting proteolysis-targeting chimeras (PROTACs), key challenges persist in the development pipeline, particularly regarding the identification of highly potent degraders and the optimization of their drug-like properties. Here, we reported a multimodal modular assembled targeting chimera (multi-MOATAC) strategy that enabled intracellular click chemistry-mediated *in situ* assembly of ligand modules with targets, integrating multiple functional units into binary/ternary complexes. This strategy validated significantly enhanced degradation efficiency via trivalent self-assembling degraders and identified a highly effective self-assembly stoichiometric ratio, achieving a DC_50_ of 4.6 ± 1.3 nM for BRD4 in MDA-MB-231 cells. To evaluate broad applicability, the target scope was expanded from nuclear protein BRD4 to challenging targets, including membrane-localized EGFR and cytoplasmic protein ALK. Furthermore, optimizing the stoichiometric ratio of self-assembling modules achieved parallel degradation of distinct targets. The superior antitumor efficacy of trivalent self-assembling PROTACs was also confirmed *in vivo*. The highly modular and scalable multi-MOATAC strategy may enable broad applications in developing novel tissue-specific degraders and advancing therapeutics for related diseases.

## Graphical abstract

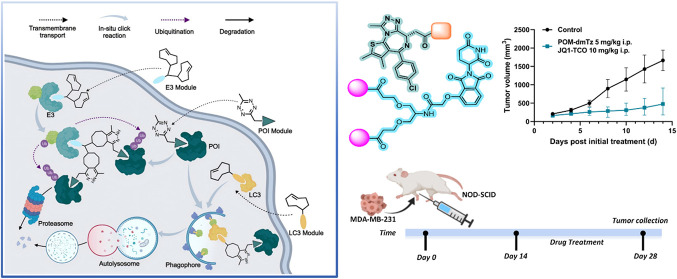

## Introduction

Targeted protein degradation (TPD) has emerged as a transformative therapeutic approach in drug discovery, offering unprecedented potential to modulate the endogenous elimination of pathogenic proteins historically deemed “undruggable”.^1^ Central to this strategy were proteolysis-targeting chimeras (PROTACs), heterobifunctional molecules that exploit the ubiquitin-proteasome system (UPS) to selectively degrade proteins of interest (POIs).^2^ Structurally, PROTACs integrate three critical components: an E3 ubiquitin ligase-binding moiety, which recruits the UPS machinery, a POI-targeting ligand, ensuring specificity and a flexible linker that spatially aligns these two domains to enable proximity-induced polyubiquitination of the POI.^3–5^ Following ubiquitination, the tagged POI was irreversibly processed by the 26S proteasome for degradation.^6–8^ In addition, novel heterobifunctional molecules that connect various effectors to various targets have been developed. For example, the autophagosome-tethering compound (ATTEC) and autophagy-targeting chimera (autotac) technologies developed in 2019 and 2022, respectively, degrade POI through the autophagy lysosome pathway. The key ligand targeting LC3 has also been validated for the degradation of other targets. By harnessing endogenous degradation pathways, TPD exemplifies a paradigm shift in precision medicine.^9–12^

With the vigorous development of small-molecule degraders, heterobifunctional molecules have also been updated. Traditional bivalent PROTACs, which incorporate a single E3 ligase ligand per molecule, can be termed “single-target PROTACs”.^13^ Trivalent PROTACs, however, open new avenues for PROTAC optimization. In 2015, Alessio Ciulli’s group developed the bivalent PROTAC MZ1, which unexpectedly achieved selective degradation of BRD4 through synergistic effects of a BET inhibitor and a VHL ligand.^14^ Further analysis of the BRD4-MZ1-VHL ternary complex crystal structure led to the development of trivalent PROTACs featuring an additional target protein ligand. These molecules, employing PEG3/PEG4 linkers coupled with VHL or CRBN ligands, demonstrated potent antiproliferative activity in BET-sensitive MV4–11 cells.^15,16^ Mechanistic studies revealed that trivalent PROTACs form stable ternary complexes with VHL at a 1:1:1 molar ratio, prolonging the intracellular retention via positive cooperative effects. Notably, in 2024, the Ciulli group further refined this design by proposing a dual-E3 ligase corecruitment strategy. They successfully developed the heterotrivalent PROTAC AB3067, comprising CRBN and VHL ligands linked to a BET inhibitor via a branched trifunctional connector. This molecule significantly reduced off-target effects associated with CRBN/VHL cross-degradation.^17^ Inspired by dual-target drugs and “two-headed” PROTACs, Li’s group pioneered trivalent PROTACs capable of simultaneously degrading EGFR and PARP.^18^ Using star-shaped linkers constructed via natural amino acids and click chemistry, they integrated gefitinib (EGFR inhibitor), olaparib (PARP inhibitor), and CRBN/VHL ligands. Although its anticancer activity remains at micromolar levels due to challenges such as molecular weight and solubility, this groundbreaking design lays the foundation for multitarget applications of PROTAC technology.

While multivalent PROTACs demonstrate significant advantages in regulating multiple targets and enhancing efficacy, their large molecular weights and complex synthesis routes severely limit practical application. In improving the drug-like properties of PROTAC, Heightman et al. verified that heterobifunctional molecules can form in cells by a bioorthogonal click combination of two smaller precursors.^19^ They selected the inverse electron-demand Diels–Alder (IEDDA) cycloaddition between tetrazine and trans-cyclooctene (TCO) from the reported bioorthogonal reactions to form the in-cell click-formed proteolysis-targeting chimeras (CLIPTACs),^20^ and degrade two key oncology targets, BRD4 and ERK1/2. This method provides more possibilities for the development of novel PROTACs. While CLIPTACs and multivalent PROTACs have demonstrated promising advancements, several limitations remain to be addressed. For example, although trivalent PROTACs exhibit superior activity, their clinical translation was constrained by the inherent challenges of high molecular weight compounds. Furthermore, the synthetic complexity of the required small molecule currently restricts the broader application of multivalent strategies for targeting diverse protein families. Therefore, we envision establishing a modular, self-assembled trivalent PROTAC strategy to improve the drug-like properties of trivalent degraders and enhance targeted protein degradation efficiency and multitarget degrading ability.

Herein, we report a multimodal modular assembled targeting chimera (multi-MOATACs) strategy that integrates the high efficacy of trivalent PROTACs with the modular flexibility of CLIPTAC technology. This approach enables the in situ self-assembly of diverse ligand modules via intracellular click reactions, facilitating the construction of ternary or quaternary complexes capable of integrating multiple functional units. Using this platform, a highly potent and selective trivalent BRD4 multi-MOATAC was successfully identified, demonstrating the powerful screening potential of the method.

Furthermore, the target scope was extended from nuclear protein BRD4 (a readily degradable substrate) to more challenging targets, including membrane-localized EGFR and ALK, and this strategy was also successfully adapted to the lysosomal degradation pathway, greatly expanding the arsenal of degradation mechanisms that can be used for targeted protein clearance by multi-MOATAC. Additionally, parallel degradation of distinct targets was achieved through optimized stoichiometry of the self-assembling modules. Importantly, the therapeutic potential of this strategy was validated *in vivo*, where it significantly inhibited tumor growth in xenograft mouse models.

This multi-MOATACs platform represents a modular and scalable small-molecule self-assembling system that combines design flexibility, enhanced efficacy, and expandable modality, demonstrating broad promise for targeted protein degradation and related therapeutic applications.

## Results and Discussion

### Design and Synthesis of Multi-MOATACs Toolkit

In order to realize the intracellular self-assembly of functional molecules, tetrazine (**Tz**) and trans-cyclooctene (**TCO**) were selected as the bioorthogonal module. The reaction had fast kinetics, high yield, catalyst-free operation, and excellent biocompatibility, which had many documented biological applications.^21^

The concept of the multi-MOATACs strategy was illustrated in Figure 1. TCO or Tz modules were conjugated to the corresponding target protein ligands or E3 ligase ligands. After transmembrane transport into cells and binding to target proteins, these modules self-assemble into targeting chimeras in cells and induce the corresponding target proteins or effectors (such as E3) to form ternary or quaternary complexes. Guided by this scheme, we synthesized a functional compound library termed the “Module-assembled degrader toolkit” (Figure 1). The study initially selected BRD4 as a model degradable target for validating the modular PROTAC design, employing JQ1 as the warhead ligand. To achieve trivalent PROTAC self-assembly, we engineered a toolbox module containing dual click-chemistry units, enabling a systematic comparison with conventional bivalent PROTAC constructs. For E3 ligase recruitment, we selected widely used CRBN ligand pomalidomide and VHL ligand VH032.^22^ Additionally, to verify the possibility that the lysosomal pathway was involved in this strategy, two LC3 ligands (AN2 and GW5074) were used as LC3-recruiting units.^23^ These ligands were functionalized with single or dual bioorthogonal units, collectively constituting the self-assembling compound library. The modular design enables the combinatorial assembly of bivalent or trivalent degraders through diverse linkage combinations, thereby establishing an efficient platform for rapid structural screening.

**Figure 1:**
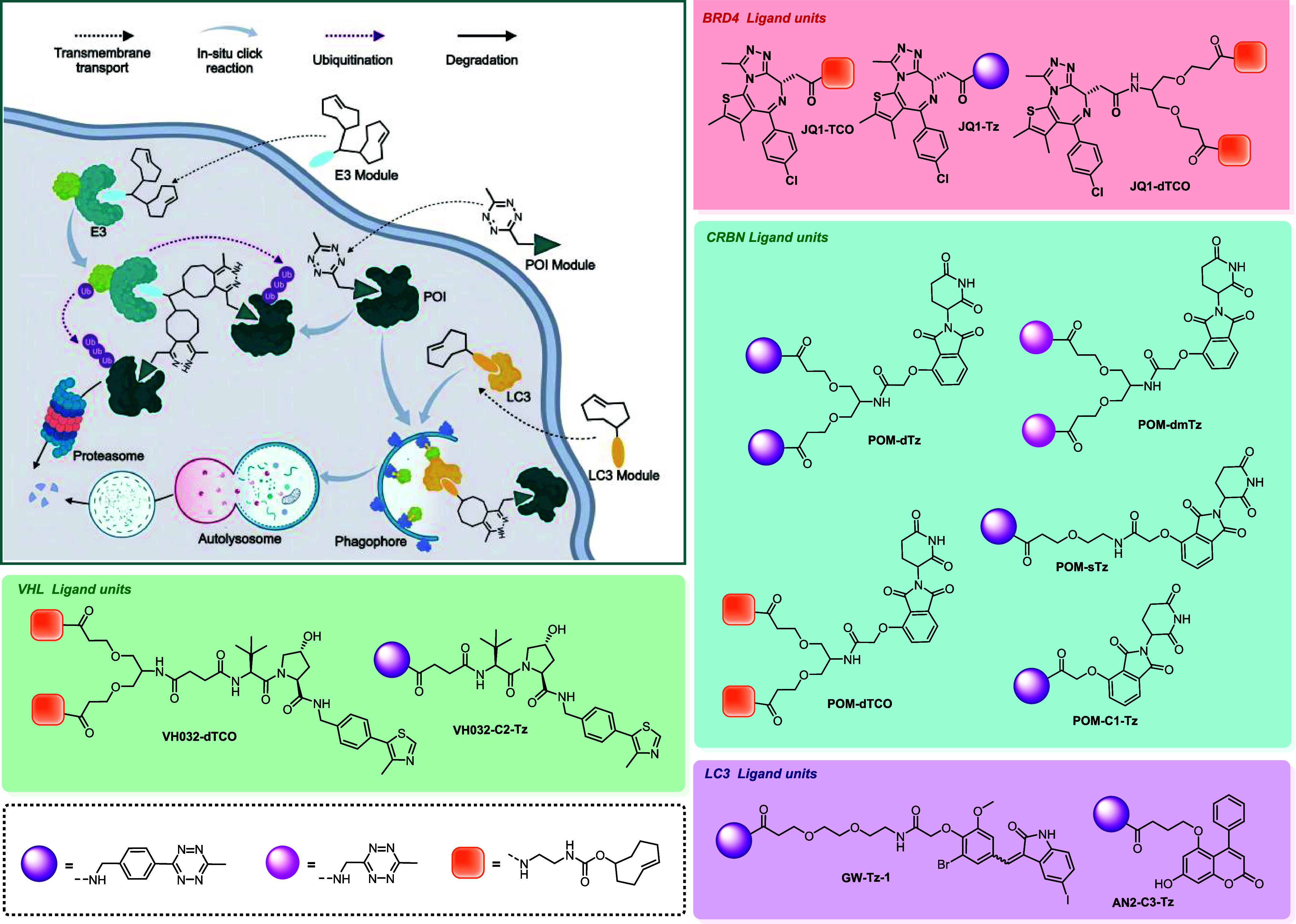
Working model of multi-MOATACs strategy and module-assembled degrader toolkit.

### Trivalent Self-Assembled Degraders Demonstrate Enhanced Degradation Efficiency *In Vitro*

Following the synthesis of the self-assembly toolbox, the target protein degradation effect was validated *in vitro*. To systematically evaluate the BRD4 degradation performance of modularly assembled degraders in MDA-MB-231 cells, 11 distinct stoichiometric assembly combinations were employed for screening (Table 1).

**Table 1:** Module Combination for Screening

no.	module combination
I	POM-dTz:JQ1-TCO = 1:2
II	POM-sTz:JQ1-TCO = 1:1
III	JQ1-dTCO:POM-C1-Tz = 1:2
IV	POM-dTCO:JQ1-Tz = 1:2
V	POM-dTz:JQ1-dTCO = 1:1
VI	JQ1-dTCO:VH032-C2-Tz = 1:2
VII	VH032-dTCO:JQ1-Tz = 1:2
VIII	JQ1-dTCO:GW-Tz-1 = 1:2
IX	JQ1-TCO:GW-Tz-1 = 1:1
X	JQ1-dTCO:AN2-C3-Tz = 1:2
XI	JQ1-TCO:AN2-C3-Tz = 1:1

To confirm the cell permeability of the degradation module, the successful *in situ* click of the module in cells was confirmed by the change in the fluorescence characteristics of the Tz unit after the click reaction (Figure S1). In MDA-MB-231 cells, Western blot analysis of BET protein levels was performed across these 11 module assembly combinations (**I-XI**) at five concentration gradients. As shown in the results, assembly combination **I** (Figure 2a,f) exhibited optimal BRD4 degradation activity and selectivity, and both the degradation potency and selectivity for BRD4 were greatly improved compared with traditional PROTAC **dBET1** (Figure S2). Compared to **II** (Figure 2b,g), the dual BRD4 ligand combination significantly enhanced the degradation efficiency. Notably, trivalent self-assembling degraders outperformed their bivalent counterparts: at 10 nM, the trivalent system achieved over 50% degradation of BRD4, while the divalent system showed almost no degradation. It was worth mentioning that the assembly combination **I** had good selectivity for BRD4, which was not present in the non-self-assembled trivalent PROTAC.^15^ Furthermore, the degradation efficiency in assembly combination **III** (Figure 2c,h) indicated that one BRD4 ligand module still maintained good degradation activity when assembled with two CRBN ligand modules, but not as good as assembly combination **I**. This difference may be due to the fact that the two JQ1 ligands could simultaneously bind to the BD1 and BD2 domains of BRD4, thereby enhancing the opportunity for the formation of ternary/quaternary complexes.

**Figure 2:**
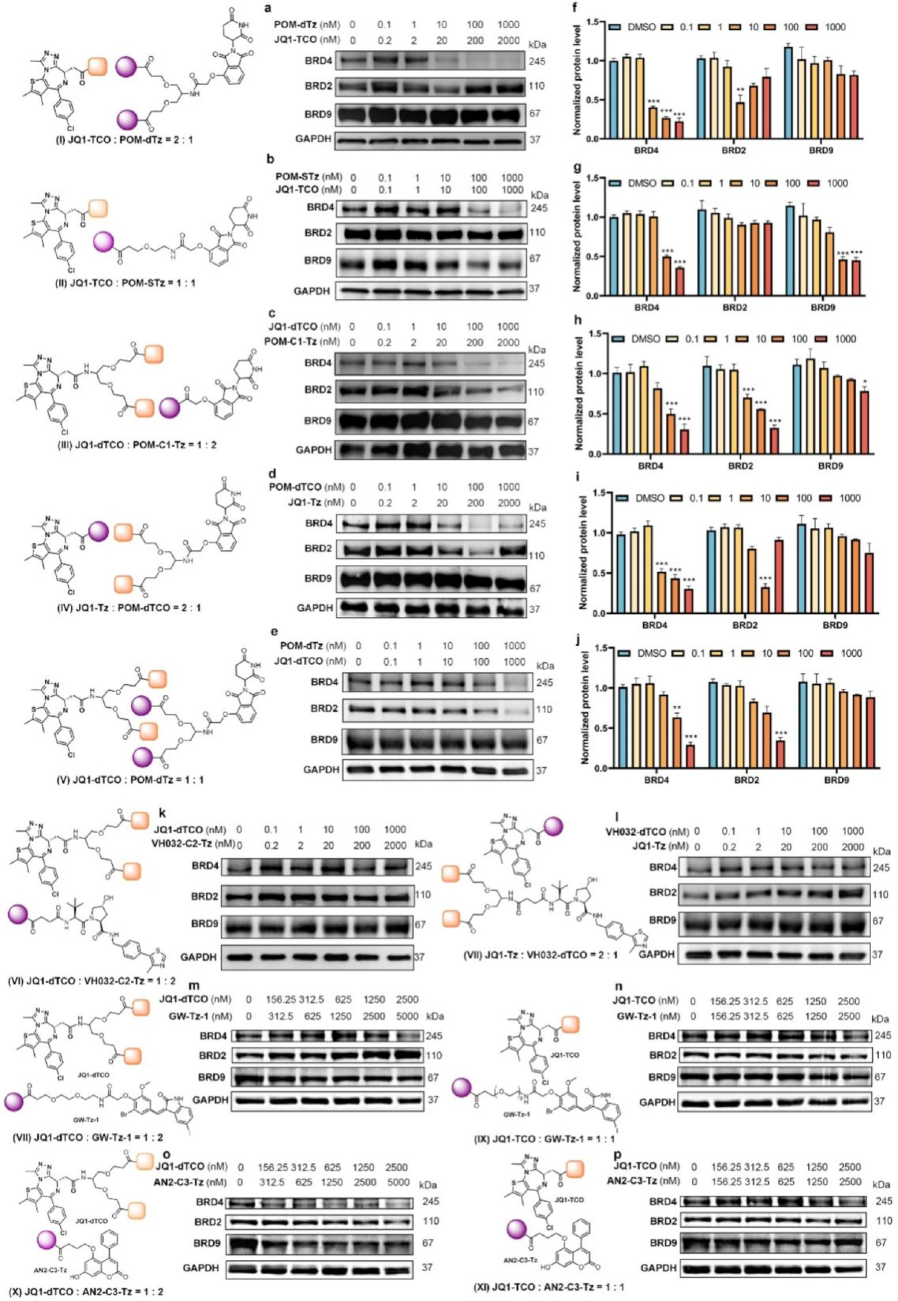
Preliminary screening of the degradation toolkit. (a) Immunoblot assay of BET protein in MDA-MB-231 cells after treatments with different concentrations of assembly combination **I** for 24 h. (b) Immunoblot assay of BET protein in MDA-MB-231 cells after treatments with different concentrations of assembly combination **II** for 24 h. (c) Immunoblot assay of BET protein in MDA-MB-231 cells after treatments with different concentrations of assembly combination **III** for 24 h. (d) Immunoblot assay of BET protein in MDA-MB-231 cells after treatments with different concentrations of assembly combination **VI** for 24 h. (e) Immunoblot assay of BET protein in MDA-MB-231 cells after treatments with different concentrations of assembly combination **V** for 24 h. (f–j) Quantitative analyses of (a–e), respectively. (k) Immunoblot assay of BET protein in MDA-MB-231 cells after treatments with different concentrations of assembly combination **VI** for 24 h. (l) Immunoblot assay of BET protein in MDA-MB-231 cells after treatments with different concentrations of assembly combination **VII** for 24 h. Quantitative data were represented as the mean ± SEM of three independent replicates. Statistical significance was assessed by one-way ANOVA (n.s.: not significant, *: *p* < 0.05, **: *p* < 0.01, ***/###: *p* < 0.001). (m–p) Validation of lysosome pathway self-assembly module. Immunoblot assay of BET protein in MDA-MB-231 cells after treatments with different concentrations of assembly combination VIII-XI for 24 h.

It was interesting that combination **IV** (Figure 2d,i), in which the **TCO** and **Tz** components were reversed, induced a significant hook effect on the degradation of BRD2 compared to combination **III**, with a change of the effective concentration range. This suggested that subtle spatial reconstruction after assembly may critically regulate degradation efficacy by altering the synergistic effect between E3 ligase and target binding. The combination of **V** (Figure 2e,j) exhibited moderate degradation activity, likely due to the formation of cyclic PROTACs during self-assembly. Moreover, this observation confirmed that cyclized PROTAC architectures retained functional bioactivity. For the VH032-based assembly combination (**VI–VII**, Figure 2k,i), no significant target degradations were observed. This inefficiency suggested that the module might require linker optimization to adjust the spatial proximity between the VHL and BRD4.

Notably, the LC3 module self-assembly also exhibited appreciable degradation capacity at high concentrations (2500 nM), with the trivalent form showing marginally superior efficacy to its divalent counterpart, indicating that this strategy remained functional in the lysosomal degradation pathway (**VIII–XI**, Figure 2m,p).

After assembly combination **I**, as the optimal assembly combination, further drug-like modifications were performed on combination **I** to obtain combination **XII**. According to literature reports,^24^ the methyl tetrazine (**mTz**) unit has better stability, and the benzene-containing **Tz** in compound **POM-dTz** was further replaced with **mTz** units (Figure 3a,b). This modified combination (**XII**) slightly enhanced the efficacy, achieving a DC_50_ of 4.6 ± 1.3 nM (calculated based on **POM-dmTz** concentration, equivalent to the theoretical trivalent complex concentration postassembly) and *D*_max_ of 89.3% after 24-h treatment in MDA-MB-231 cells. This optimized assembly combination (**XII**) was selected for further mechanistic and kinetic studies. Dynamic monitoring of degradation kinetics under treatment with 10 nM **POM-dmTz** and **20** nM **JQ1-TCO** over 0–36 h revealed a gradual reduction in target protein levels as exposure time increased. Statistical significance (*p* < 0.05) was observed at 8 h, with degradation reaching a plateau phase by 12 h (Figure 3c,d).

**Figure 3:**
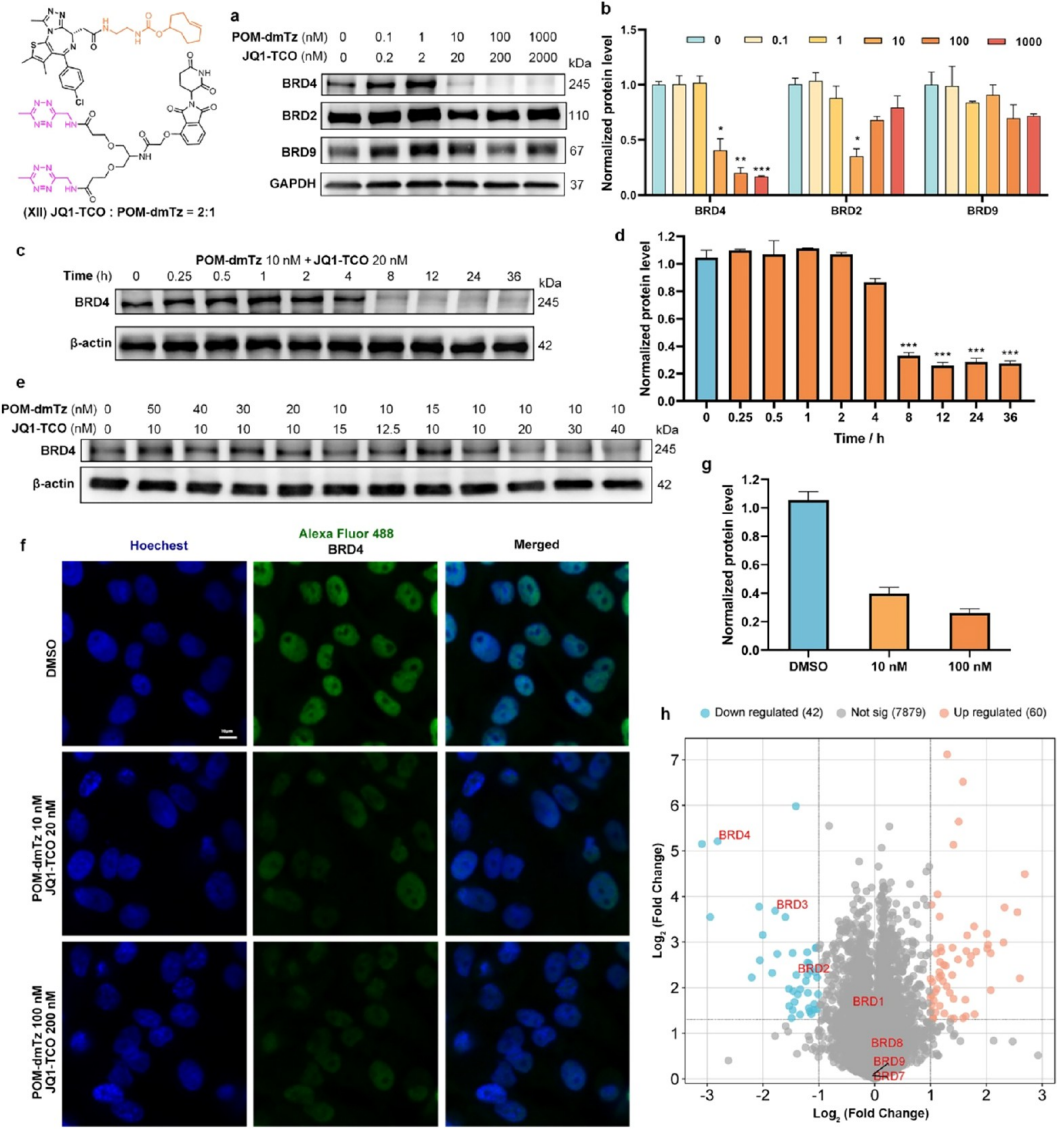
Degradation activity of self-assembled POM-dmTz: JQ1-TCO (1:2 ratio). (a) Immunoblot assay of BET protein in MDA-MB-231 cells after treatments with different concentrations of assembly combination **XII** for 24 h. (b) Quantitative analysis of BET protein levels in (a). (c) Western blot analysis of target protein degradation in MDA-MB-231 cells treated with assembly combination **XII** at the indicated time points. (d) Quantitative analyses of target protein levels in panel c. (e) Western blot analysis of target proteins in MDA-MB-231 cells treated with varying **POM-dmTz**: **JQ1-TCO** ratios for 24 h. (f) Representative confocal LSM images of BRD4 immunofluorescence (Alexa Fluor 488, green) in MDA-MB-231 cells under different conditions (scale bar: 20 μm). (g) Quantification of BRD4 fluorescence intensity in panel f. (h) Volcano plot of DIA proteomic analysis in MDA-MB-231 cells treated with **POM-dmTz**: **JQ1-TCO** (1:2) for 8 h, depicting log_2_-transformed fold changes (*x*-axis) versus -log_10_(*p*-values) from Student’s *t* test (*y*-axis) for protein abundance differences. Quantitative data were represented as the mean ± SEM of three independent replicates; statistical significance was assessed by one-way ANOVA (n.s.: not significant, *: *p* < 0.05, **: *p* < 0.01, ***/###: *p* < 0.001).

In addition, the influence of the ratio between **POM-dmTz** and **JQ1-TCO** in assembly combination **XII** on degradation activity was further investigated. The results demonstrated that higher **JQ1-TCO** proportions enhanced degradation efficiency, while excessive **POM-dmTz** concentrations reduced degradation activity (Figure 3e). The dosing intervals of the two degradation modules were also investigated, and it was found that the degradation activity decreased when the administration was not interrupted, while an interval of 15 min or more could exert a good degradation effect (Figure S3). The immunofluorescence staining results analyzed by confocal laser scanning microscopy (CLSM) showed a dose-dependent decrease in BRD4 fluorescence intensity (Figure 3f,g). DIA-based quantitative proteomics revealed a significant downregulation of BRD4 protein levels in MDA-MB-231 cells following 8 h treatment with assembly combination **XII** (Figures 3h and S4), demonstrating high selectivity over other BET family members, which is consistent with the results of Western blot analysis.

### Expanding the Target Landscape of the Multi-MOATACs Strategy

Building upon the previously demonstrated robust degradation activity of self-assembling trivalent degraders and their superior protein degradation efficacy compared to that of bivalent architectures, this study further investigated the generality of this module self-assembling strategy. To evaluate the potential of the platform in multitarget applicability and cross-cell line adaptability, we assessed the degradation ability of the membrane protein target EGFR and cytoplasmic protein ALK in PC9 cells using this strategy.

Western blot analysis of the EGFR module (Figure 4a,b)-assembled treatment in PC9 cells (Figure 4c) revealed that the module-assembled degraders significantly reduced EGFR protein levels after 24 h treatment (Figure 4c), with DC_50_ values of 2.3 ± 0.2 and 4.2 ± 0.3 μM, respectively, demonstrating that trivalent systems (Figure 4c) have higher degradation efficiency than bivalent systems (Figure 4d). Immunofluorescence confocal microscopy further confirmed a marked reduction in the EGFR signal intensity following trivalent complex treatment (Figure 4g,h). Similarly, in ALK degradation experiments, the trivalent system (Figure 4d) outperformed the bivalent counterpart (Figure 4e), with DC_50_ values of 1.6 ± 0.1 and 5.0 ± 0.7 μM, respectively.

**Figure 4:**
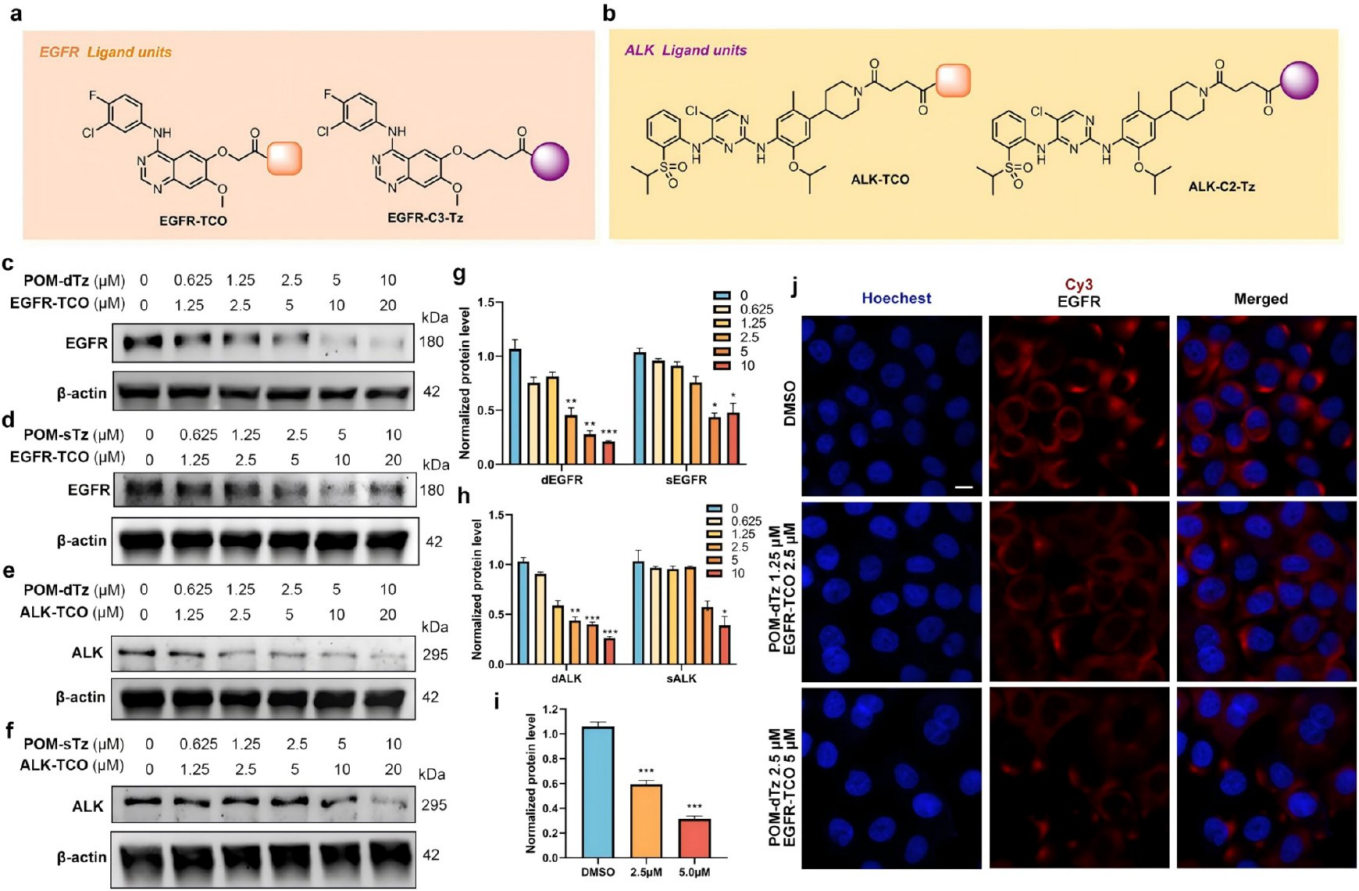
Target expansion profiling of self-assembled trivalent degraders: (a) Structure of EGFR ligand units; (b) structure of ALK ligand units; (c) Western blot analysis of target proteins in PC9 cells treated with POM-dTz and EGFR-TCO (1:2) at varying concentrations for 24 h; (d) Western blot analysis of ALK degradation in PC9 cells treated with POM-sTz and EGFR-TCO; (e) Western blot analysis of ALK degradation in PC9 cells treated with POM-dTz and ALK-TCO (1:2) at varying concentrations for 24 h; (f) Western blot analysis of ALK degradation in PC9 cells treated with POM-dTz and ALK-TCO (1:1) at varying concentrations for 24 h; (g) quantification of protein degradation efficacy from panels c and d; (h) quantification of protein degradation efficacy from panels e and f; (i) quantification of protein degradation efficacy from panel j; (j) representative confocal microscopy images of EGFR immunofluorescence (CY3, red) in PC9 cells under different self-assembly ratios (fixed with paraformaldehyde; scale bar: 20 μm). Quantitative data were represented as the mean ± SEM of six (for CCK-8) or three (for image analysis) independent replicates; statistical significance was assessed by one-way ANOVA (n.s.: not significant, */#: *p* < 0.05, **/##: *p* < 0.01, ***/###: *p* < 0.001).

Furthermore, we also verified that systematic regulation of the stoichiometric ratio between different target binding ligands could control dual-target degradation (Figure 5). Immunoblotting of PC9 cells treated with varying ratios of self-assembly components for 24 h showed that at a **POM-dmTz**: **JQ1-TCO**: **EGFR-TCO** ratio of 1000:200:1800 nM, degradation efficiencies for BRD4 and EGFR reached 78.9 and 60.5%, respectively (Figure 5). This dose-dependent dual-target degradation likely arose from synergistic ligand binding within the multivalent assembly, where optimized ratios stabilize ternary complexes between the E3 ligase and distinct target proteins.

**Figure 5:**
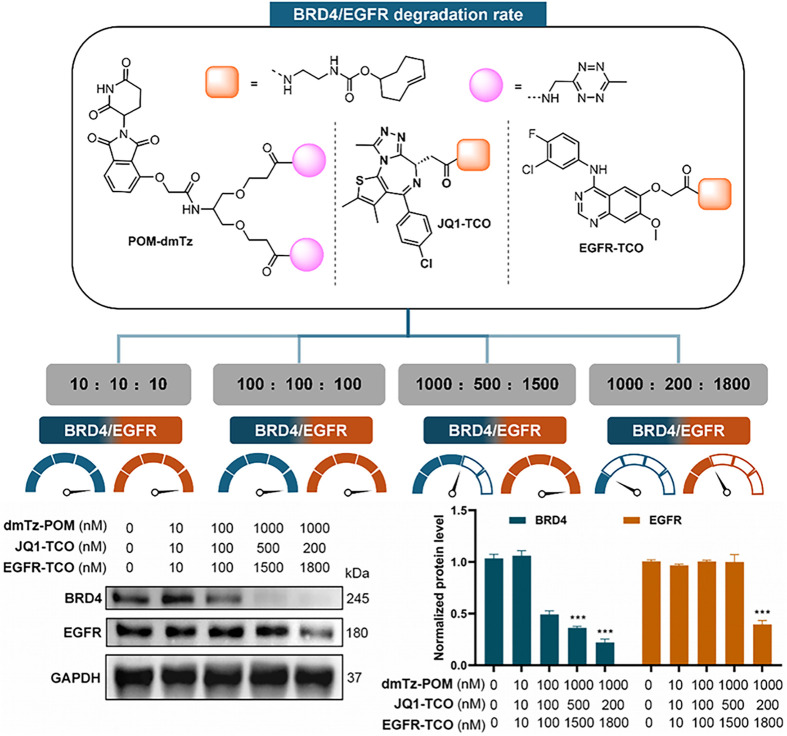
Dual-target degradation of BRD4 and EGFR. Quantitative data were represented as the mean ± SEM of six (for CCK-8) or three (for image analysis) independent replicates; statistical significance was assessed by one-way ANOVA (n.s.: not significant, */#: *p* < 0.05, **/##: *p* < 0.01, ***/###: *p* < 0.001).

However, when the **JQ1-TCO**: **EGFR-TCO** molar ratio exceeded 1:9, EGFR was significantly degraded, suggesting potential competitive binding between ligands. This finding not only validated the chemically tunable nature of multitarget degradation but also provided a rationale for developing “multitarget switchable” degraders. We hypothesized that rational extension of ligand linker lengths (e.g., increasing the **JQ1-TCO** linker to 9–12 methylene units) might reduce steric hindrance, broaden the dual-target degradation window, and establish a conceptual framework for developing multitarget regulatory networks in precision medicine.

Despite the successful degradation of membrane targets (EGFR DC_50_ ≈ 2.7 μM), efficacy remained lower than that observed for nuclear protein BRD4 (DC_50_ ≈ 4 nM). This discrepancy might relate to reduced accessibility of membrane proteins’ tertiary conformations, transmembrane domain steric effects, or structural features unique to BRD4’s BD1/2 domains. Future optimization of linker length and chemistry may be possible for improving ternary complex topological compatibility. Furthermore, the multitarget adaptability of this self-assembly strategy, while preserving specificity, will establish a novel technical framework for developing broad-spectrum protein degradation therapies.

### Combination XII Mediates Targeted Protein Degradation via the Ubiquitin-Proteasome System

Following the validation of the target protein degradation efficacy of combination **XII**, its action mechanism was further investigated (Figure 6). The results showed that neither component alone triggered detectable degradation of the target protein, indicating that the cooperative interaction of both components was strictly required for degradation (Figure 6a,c). Inhibition experiments confirmed the degradation pathway: cotreatment with the proteasome inhibitor **MG132** significantly suppressed degradation efficiency (Figure 6b,d), while the NEDDylation pathway inhibitor **MLN4924** also effectively blocked the process, confirming that this trivalent self-assembling system relied on proteasome-mediated proteolysis.

**Figure 6:**
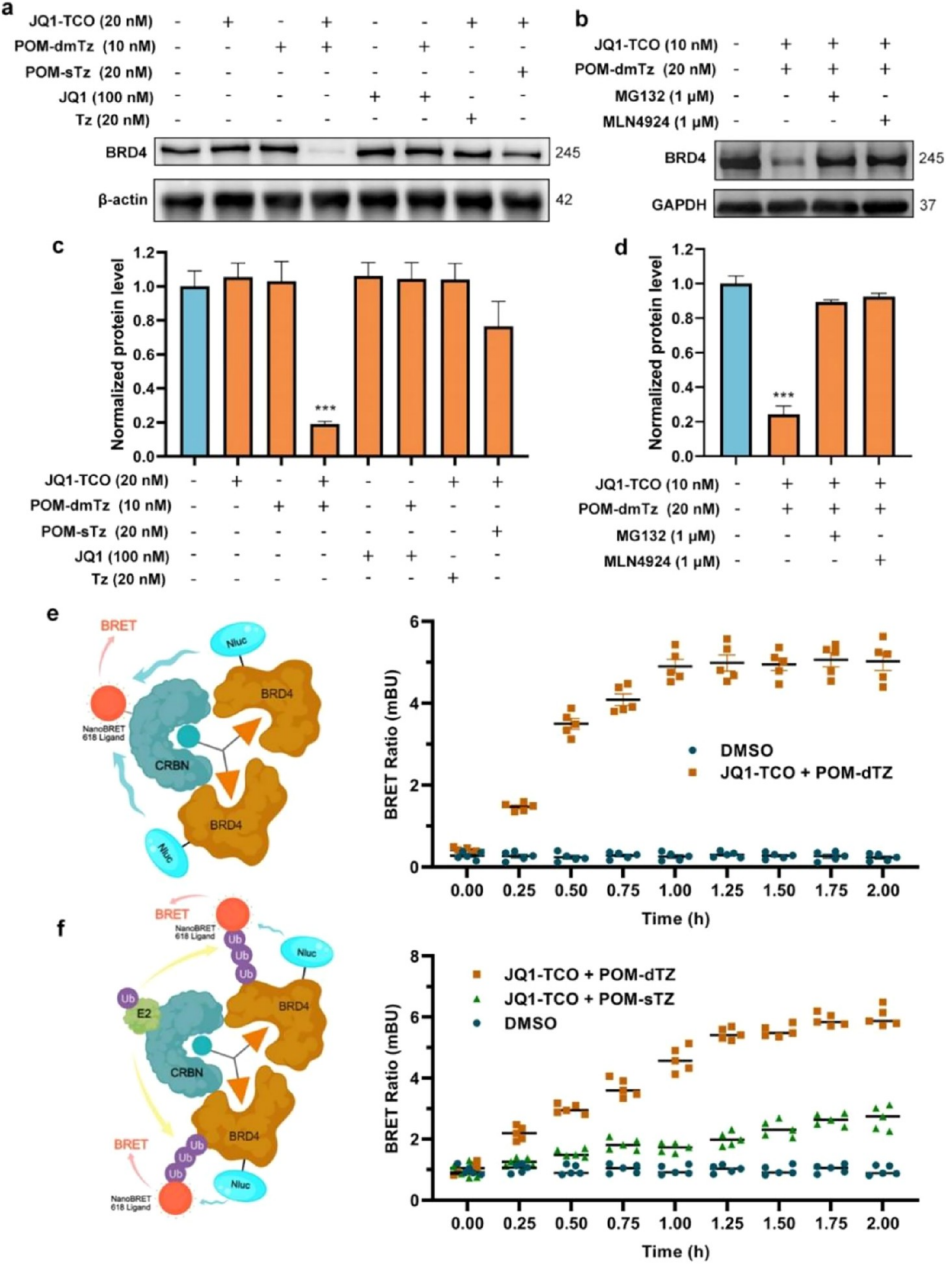
Degradation mechanism evaluation of self-assembled trivalent degraders. (a) Immunoblot for BRD4 and actin showing the effects of JQ1-TCO and POM-dmTz (POM-sTz) alone and the effects of preventing the click reaction using JQ1 and Tz on BRD4 protein levels, demonstrating that the degradation effect was dependent on in situ self-assembly. Experiments performed on MDA-MB-231 cells. (b) Functional suppression validation via catalytic degradation activity in MDA-MB-231 cells. (c) Quantitative analysis of panel a. (d) Quantitative profiling of panel b. (e) BRET signal dynamics of self-assembled complexes monitored at 15 min intervals. (f) BRET-based detection of ubiquitin-BRD4 proximity to assess target ubiquitination levels. Quantitative data were represented as the mean ± SEM of three independent replicates. Statistical significance was assessed by one-way ANOVA (n.s.: not significant, *: *p* < 0.05, **: *p* < 0.01, ***/###: *p* < 0.001).

Nano-BRET analysis revealed that the self-assembled PROTAC formed a ternary BRD4-PROTAC-CRBN complex, demonstrating its molecular capacity to spatially induce the proximity between the E3 ligase and the target protein. In addition, compared with the bivalent self-assembly combination **II**, the trivalent self-assembly combination **XII** exhibited stronger ubiquitination induction activity, and a significant increase in target protein ubiquitination was observed. This indicated that the trivalent topology structure improved the efficiency of polyubiquitination, thereby enhancing the degradation efficiency (Figure 6e,f).

Collectively, these findings demonstrated that the target protein, degrader, and E3 ubiquitin ligase formed a complex, leading to elevated ubiquitination of the target protein, and showed that the trivalent self-assembled degrader induced target protein degradation via the ubiquitin-proteasome system.

### Combination XII Exhibited Potent Antiproliferative Effects in Breast Cancer Cells

After confirming the BRD4 degradation efficacy of the assembly combination **XII**, we evaluated its cytotoxicity and antiproliferative activity against breast cancer cells (Figure 7a). Using CCK-8 assays, the IC_50_ values for three breast cancer cell lines (MCF-7, MDA-MB-231, and MDA-MB-361) were determined as 5.8 ± 0.4, 3.3 ± 0.2, and 8.1 ± 0.3 nM, respectively, demonstrating potent cytotoxicity capacities.

**Figure 7:**
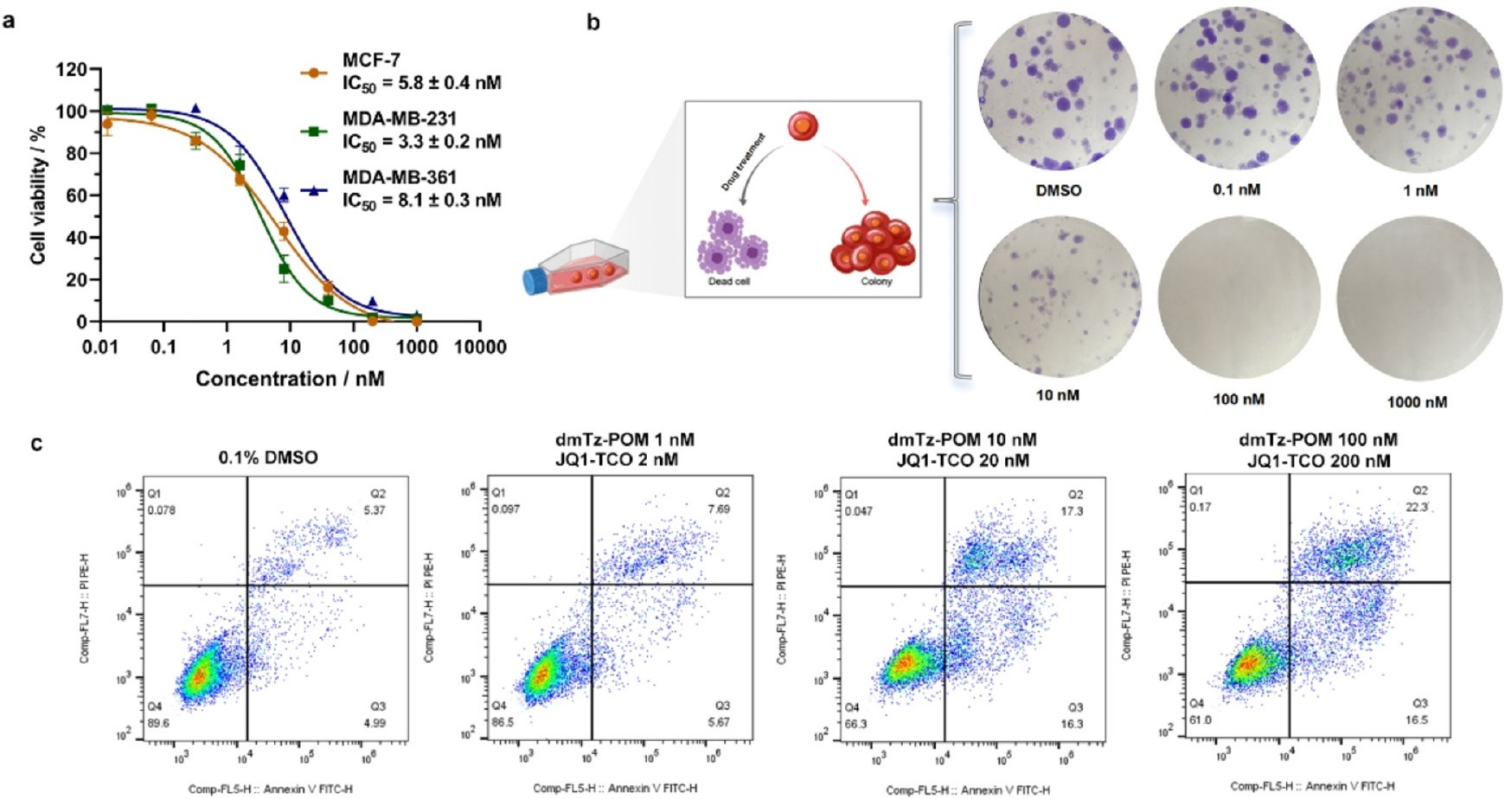
Cytotoxicity and antiproliferative activity of the POM-dmTz: JQ1-TCO (1:2) self-assembled ratio. (a) CCK-8 cytotoxicity assay of POM-dmTz: JQ1-TCO (1:2) self-assembly in three breast cancer cell lines. (b) Colony formation assay of MDA-MB-231 cells post-treatment. (c) Flow cytometry analysis of apoptosis in MDA-MB-231 cells via Annexin V/PI dual staining.

Colony formation assays demonstrated that 10 nM **POM-dmTz** and 20 nM **JQ1-TCO** significantly suppressed the clonogenic capacity of MDA-MB-231 cells (Figure 7b). Furthermore, Annexin V/PI dual staining confirmed robust apoptosis induction at this concentration (Figure 7c). These findings suggested that BRD4 degradation activated apoptotic pathways, driving the compound’s antitumor effects.

### Trivalent Self-Assembling PROTACs for Targeted BRD4 Degradation *In Vivo*

Given the favorable cellular performance of the **POM-dmTz** and **JQ1-TCO** stoichiometric ratio, we further investigated their *in vivo* pharmacokinetic (PK) profiles (Figure 8). To determine the PK parameters of the two compounds, Sprague–Dawley (SD) rats were administered either intravenous (i.v.) injection at 5 mg/kg or intraperitoneal (i.p.) injection at 10 mg/kg. Blood samples were collected from the jugular vein at 5 min, 15 min, 30 min, 1, 2, 4, 7, and 24 h postdosing for drug concentration analysis.

**Figure 8:**
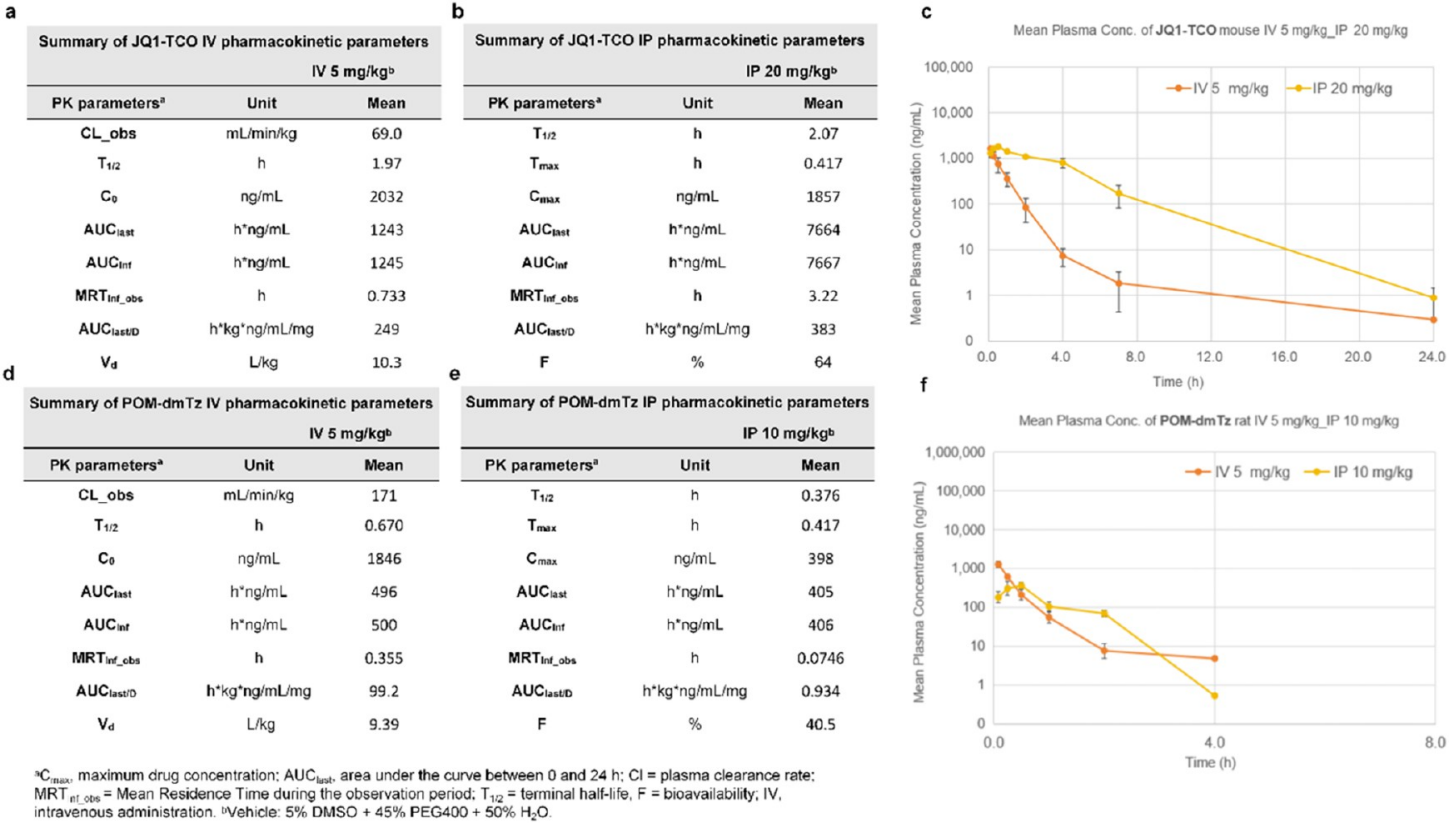
Pharmacokinetic (PK) profiles of compounds POM-dmTz and JQ1-TCO. (a, b) PK parameters of JQ1-TCO in Sprague–Dawley (SD) rats, including clearance and bioavailability. (c) Serum concentration–time profiles of JQ1-TCO under two administration routes, with serum samples collected at 5 min, 15 min, 0.5 h, 1 h, 2 h, 4 h, 7h, and 24 h postadministration for quantitative analysis (mean ± SEM; *n* = 3 mice per dose group). (d, e) PK parameters of POM-dmTz in SD rats. (f) Serum concentration–time profiles of POM-dTz following two administration routes, analyzed at identical time points (mean ± SEM; *n* = 3 mice per dose group).

Comparing the two administration methods, iv administration had a slight advantage. For **JQ1-TCO**, a half-life (*T*_1/2_) of approximately 2.07 h and high plasma concentrations were observed following i.p. administration at 10 mg/kg. However, **POM-dmTz** exhibited a shorter half-life after ip injection at 10 mg/kg. Given that in vitro experiments have validated rapid click reaction completion (∼5 min) in plasma (Figure S5), we implemented a sequential administration strategy: **JQ1-TCO** was administered first via i.p. injection, followed by **POM-dmTz** after a 10 min distribution period, as **JQ1-TCO** was administered first and **POM-dmTz** was injected 10 min later, a time point before **JQ1-TCO** reached its maximum plasma concentration, a higher dose of **JQ1-TCO** was required to ensure sufficient intracellular concentration for an efficient in vivo click reaction with the subsequently administered **POM-dmTz**. This protocol ensured rapid *in vivo* click assembly while mitigating premature clearance.

To evaluate the *in vivo* efficacy of the trivalent self-assembled degraders based on the **POM-dmTz**/**JQ1-TCO** stoichiometric ratio, a subcutaneous xenograft model of MDA-MB-231 cells was established in NOD/SCID mice to assess antitumor activity and safety. A total of 10 mice were randomized into two groups (*n* = 5 per group): (1) vehicle control and (2) treatment group (5 mg/kg of **POM-dmTz** + 10 mg/kg of **JQ1-TCO**). Intraperitoneal administration was initiated when tumor volumes reached ∼100 mm^3^, with daily dosing for 14 days (Figure 9a).

**Figure 9:**
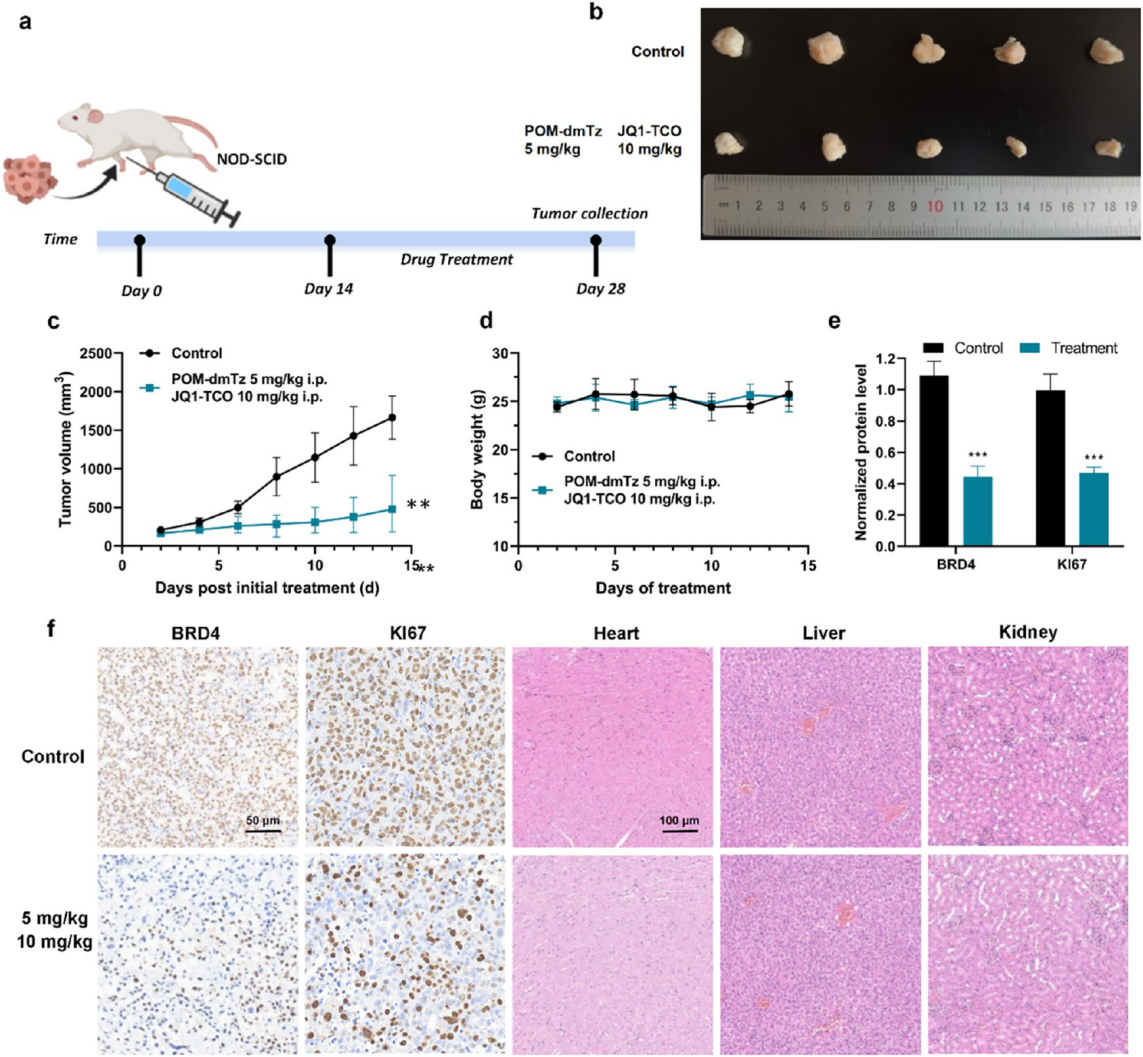
Therapeutic efficacy of trivalent self-assembling PROTAC in tumor xenograft mouse models. (a) Schematic timeline of animal experimental design. (b) Comparison of tumor tissue sizes (fixed specimens) after 14-day treatment. (c) Tumor volume dynamics were measured every 48 h postadministration. (d) Body weight changes are monitored every 48 h during treatment. (e) Quantitative analysis of BRD4 and KI67 protein expression levels. (f) Immunohistochemical (IHC) staining of BRD4 and KI67 in tumor sections (paraffin-embedded, scale bar: 50 μm), with representative microscopic images, and H&E-stained heart, liver, and kidney specimens (scale bar: 100 μm) across treatment groups. Quantitative data were represented as the mean ± SEM of three independent replicates, and statistical significance was assessed by one-way ANOVA (n.s.: not significant, */#: *p* < 0.05, **/##: *p* < 0.01, ***/###: *p* < 0.001).

The treatment group exhibited significant tumor growth inhibition (TGI) of 69.5 ± 7.2% compared to the control group (*p* < 0.01), confirming potent antitumor efficacy (Figure 9b,c). Body weight variation coefficients remained below 5% throughout the study, indicating no overt systemic toxicity at the tested dose and favorable tolerability.

Mechanistic analysis via immunohistochemical (IHC) staining revealed a marked reduction in BRD4-positive tumor areas and downregulation of the proliferation marker KI67, demonstrating that BRD4 degradation effectively disrupted tumor cell proliferation pathways (Figure 9e,f). Histopathological evaluation of major organs (heart, liver, kidney) by hematoxylin and eosin (H&E) staining showed no evidence of inflammatory infiltration, necrosis, or structural abnormalities, further supporting the biocompatibility of the degraders (Figure 9f).

Collectively, the trivalent self-assembly strategy achieved robust tumor suppression (TGI > 60%) by enhancing target engagement avidity, providing critical preclinical evidence for developing multitargeted protein degradation therapies based on polyvalent interactions.

## Conclusions

In this study, we developed a multimodal modular assembled targeting chimera (multi-MOATAC) strategy. This strategy utilized the covalent coupling of target protein ligands with biocompatible click reaction modules trans- cyclooctene (**4E-TCO**) and tetrazine (**Tz**) to design modular programmable degrader precursor modules. In the cellular microenvironment, these modules carried out precise self-assembly through the efficient Diels–Alder (IEDDA) reaction between **TCO** and **Tz** to generate degraders *in situ*.

Through this strategy, trivalent self-assembled degraders were screened and a modular self-assembled trivalent molecule was developed, which showed excellent degradation efficiency in MDA-MB-231 cells, reaching a DC_50_ of 4.6 ± 1.3 nM, a maximum degradation efficiency (*D*_max_) of 89.3%, and effective antiproliferative activity (IC_50_ = 3.3 ± 0.2 nM). DIA quantitative proteomics confirmed high target specificity, while mechanism validation showed that degradation was dependent on the ubiquitin-proteasome system (UPS).

Further extending the strategy’s utility, we constructed trivalent degraders targeting transmembrane receptor kinases (EGFR and ALK), enabling degradation of both membrane-bound receptors and intracellular kinases. Notably, by modulation of ligand combinations, the strategy achieved simultaneous degradation of BRD4 and EGFR, showcasing multitarget coordination.

In xenograft tumor models, the self-assembled trivalent BRD4 degrader demonstrated significant antitumor efficacy (tumor growth inhibition, TGI > 60%) without inducing body weight loss or hepatorenal toxicity.

In summary, this work established a novel paradigm for modular degrader construction and elucidated the molecular basis of polyvalency-enhanced ternary complex stabilization. These findings provided a rapid screening method and preclinical validation for developing next-generation protein degradation therapies with multitargeting capabilities. This approach established a foundational framework for the development of next-generation PROTACs with optimized pharmacokinetic profiles, minimized off-target effects, and enhanced therapeutic efficacy, thereby providing a robust rationale for accelerating the clinical translation of high-potency trivalent PROTAC systems.

## Supplementary Material



## Notes

Direct monitoring of the click reaction in PC9 Cells (**Figure S1)**; immunoblotting of MDA-MB-231 cells after treatment with different concentrations of the bivalent degrader dBET1 (**Figure S2**); effect of administration interval of self-assembly modules on degradation efficacy, immunoblotting of BRD4 after administration for 0, 15, 30, 60, and 120 min (**Figure S3)**; bioinformatic analysis of the proteomics data (**Figure S4)**; HPLC detection chromatogram of combination XII after mixing in plasma for 5 min (**Figure S5)**; stability test of compounds **JQ1-TCO** and **POM-dmTz** in mouse plasma, with carbamazepine as the control compound (**Figure S6)**; significantly differential proteins (TOP 30) in DIA proteomic analysis of MDA-MB-231 cells treated with combination XII for 8 h (**Table S1)**; ^1^H and ^13^C NMR spectra of synthesized compounds (**Appendix A)**; HRMS spectra of synthesized compounds (**Appendix B)**; HPLC traces of synthesized compounds (**Appendix C**) (PDF)
