# Supplementary material for: Modular Assembled Targeting Chimera Enables Multimodal Targeted Protein Degradation
Source: JACS Au. 2025 Nov 5;5(11):5635–47. doi: 10.1021/jacsau.5c01131 (PMC12648310; doi:10.1021/jacsau.5c01131)
Supplement: Supplementary file 1 [file au5c01131_si_001.pdf]

SUPPORTING INFORMATION

Modular Assembled Targeting Chimera Enables Multimodal Targeted Protein Degradation

Wentao Zhu [1],Wenqian Zhang [1], Yinmiao Wang [1], Jian Chen [1], Fang Xu [2], and Jiyan Pang [1, *]

1 *School of Chemistry, Sun Yat-sen University, Guangzhou 510006, China*

2 *International Cooperative Laboratory of Traditional Chinese Medicine Modernization & Innovative Drug Development of Chinese Ministry of Education (MOE) & Guangzhou City Key Laboratory of Precision Chemical Drug Development, School of Pharmacy, Jinan University, Guangzhou 510632, China*

* Corresponding author. Jiyan Pang, E-mail: cespjy@mail.sysu.edu.cn

Table of Contents

Figure S1 .... s3
Figure S2 .... s3
Figure S3 .... s4
Figure S4 .... s4
Figure S5 .... s5
Figure S6 .... s5
Table S1 .... s2
Experimental section s7
Appendix A ^{1}H and ^{13}C NMR spectra of synthesized compounds s28
Appendix B HRMS spectra of synthesized compounds s43
Appendix C HPLC traces of synthesized compounds s51

Table S1. Significantly Differential Proteins (TOP 30) in DIA Proteomic Analysis of MDA-MB-231 Cells Treated with combination XII for 8 Hours

Protein	FC(DBRD/NT)	Pvalue(DBRD/NT)
KRT1	2.45775057374368	7.65E-08
KRT9	2.9895721031763705	3.05E-07
IFITM3	0.37639133381782	1.04E-06
CCN2	2.83562375247912	2.29E-06
DDX56	0.5669680188290961	2.82E-06
KRT2	1.19822277370688	2.89E-06
BRD4	0.142447294024212	6.15E-06
OGN	0.11717333660741	7.03E-06
CACHD1	2.65451424816653	7.37E-06
GNAI2	1.08910602133374	8.54E-06
CDC42EP4	0.8244494718925209	1.71E-05
TFRC	1.04685568792697	2.19E-05
KRT5	1.9678500425939	2.20E-05
VTN	1.8964281121099	2.83E-05
CALU	1.10596519568279	4.91E-05
ATP5MF	1.22251523790675	4.92E-05
KRT10	1.55245523776342	5.28E-05
DESI1	0.7841230913646791	5.60E-05
SDHB	1.12130350630744	5.94E-05
FASN	1.12400612724951	6.13E-05
AHNAK	1.10822344539657	6.27E-05
CAD	0.9617569323942472	6.29E-05
MDH2	1.10903605615004	6.69E-05
ETFA	1.18002421117173	6.84E-05
ZNHIT2	0.799693814812735	7.33E-05
MYBBP1A	0.9037741585471892	7.64E-05
VIM	1.12692850182481	7.67E-05
KRT6B	2.18376091133036	8.91E-05

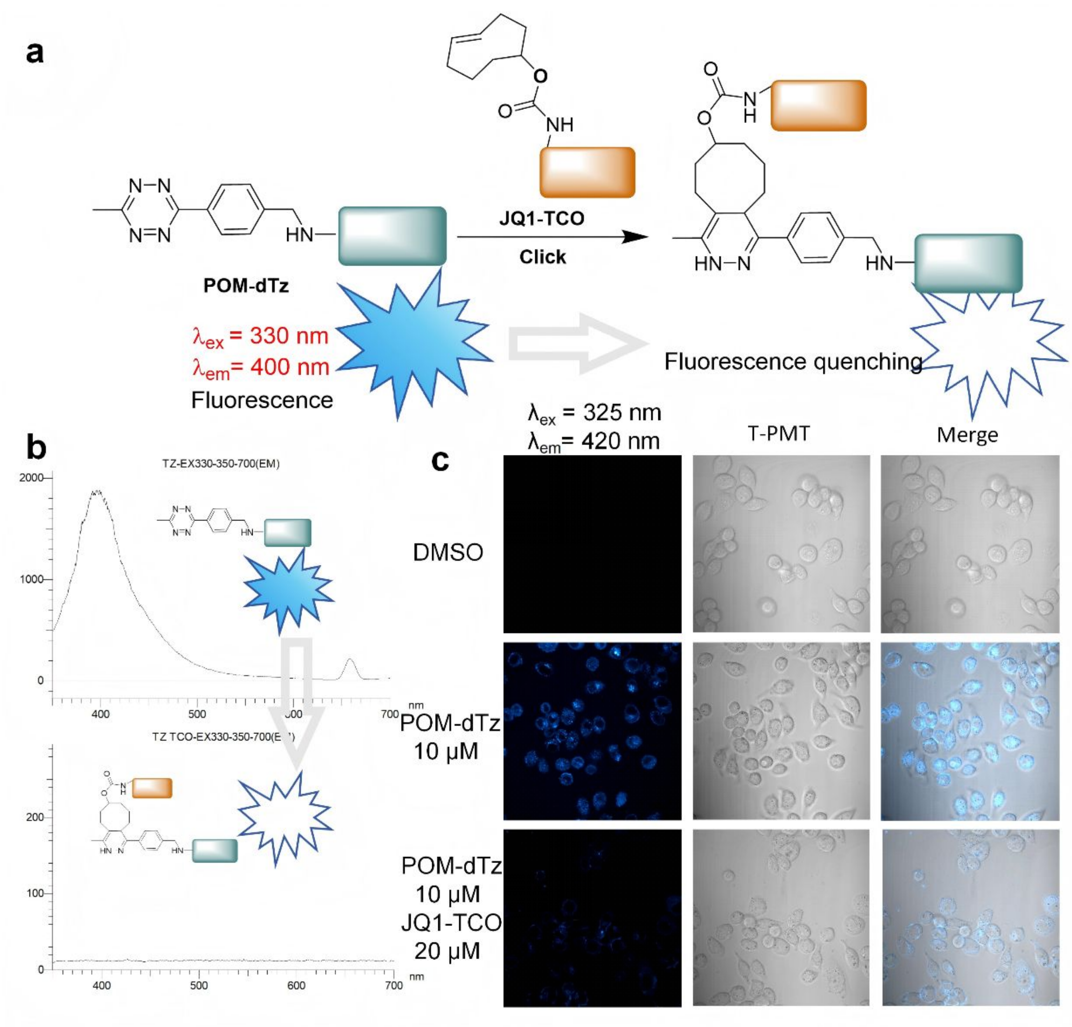

Figure S1. Direct Monitoring of the Click Reaction in PC9 Cells. (a) Schematic diagram of the fluorescence quenching phenomenon after the click reaction between the Tz and TCO. (b) Fluorescence spectrum changes after click reaction between Tz group and TCO. (c) Laser confocal images of Tz and TCO clicks under different conditions. (scale bar: 20 μm)

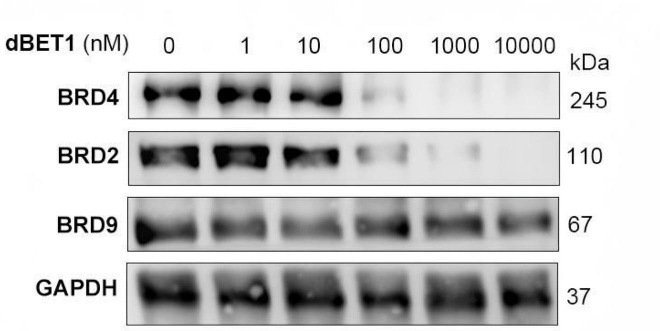

Figure S2. Immunoblotting of MDA-MB-231 cells after treatment with different concentrations of the bivalent degrader dBET1.

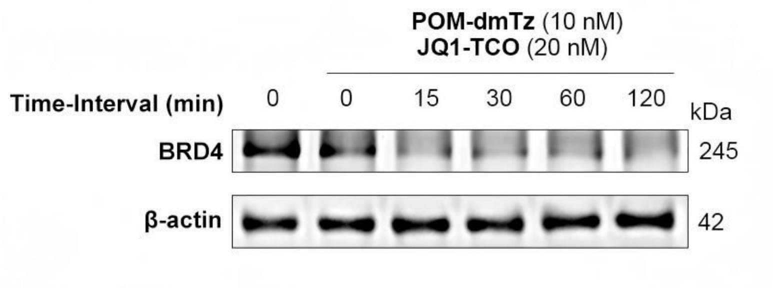

Figure S3. Effect of administration interval of self-assembly modules on degradation efficacy, Immunoblotting of BRD4 after administration for 0, 15, 30, 60, and 120 minutes.

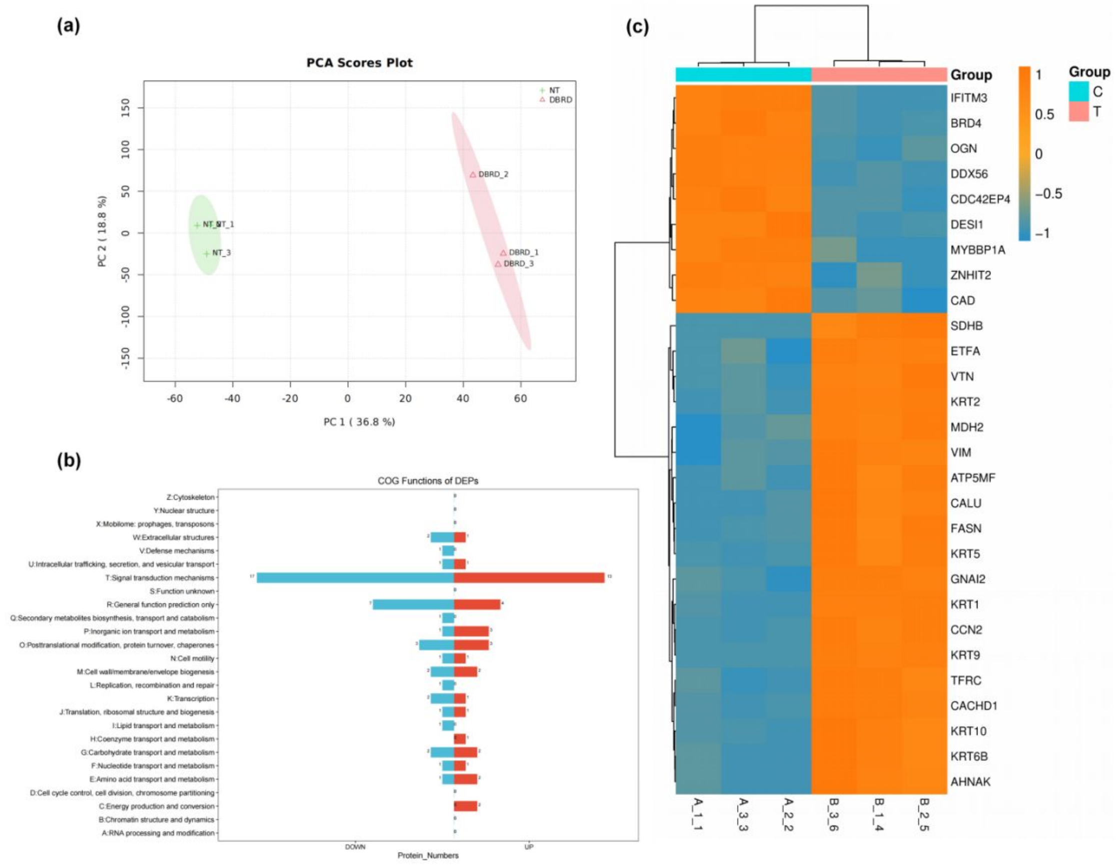

Figure S4 Bioinformatic analysis of the proteomics data. **(a)** PCA analysis of the sample, PCA score plot on PC1 and PC2 dimensions. **(b)** Differential protein COG annotation. Each bar in the figure represents a functional classification cluster of COG. The bar pointing to the left indicates the number of down-regulated proteins, while the bar pointing to the right indicates the number of up-regulated

proteins. The longer the bar, the more proteins belong to this classification cluster; the ordinate represents the functional name description of the classification cluster, and the capital letter preceding the term is the abbreviation of the functional name. **(c)** Heatmap showing the expression profiles of identified DEPs in each replicate (TOP 30).

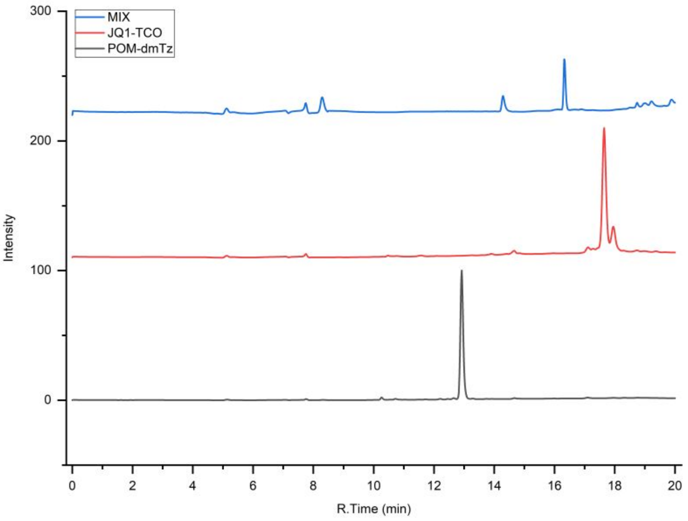

Figure S5. HPLC detection chromatogram of combination XII after mixing in plasma for 5 minutes. (Shimadzu, LC-20A; Waters XSelect HSS T3 column; 90% Acetonitrile/Water)

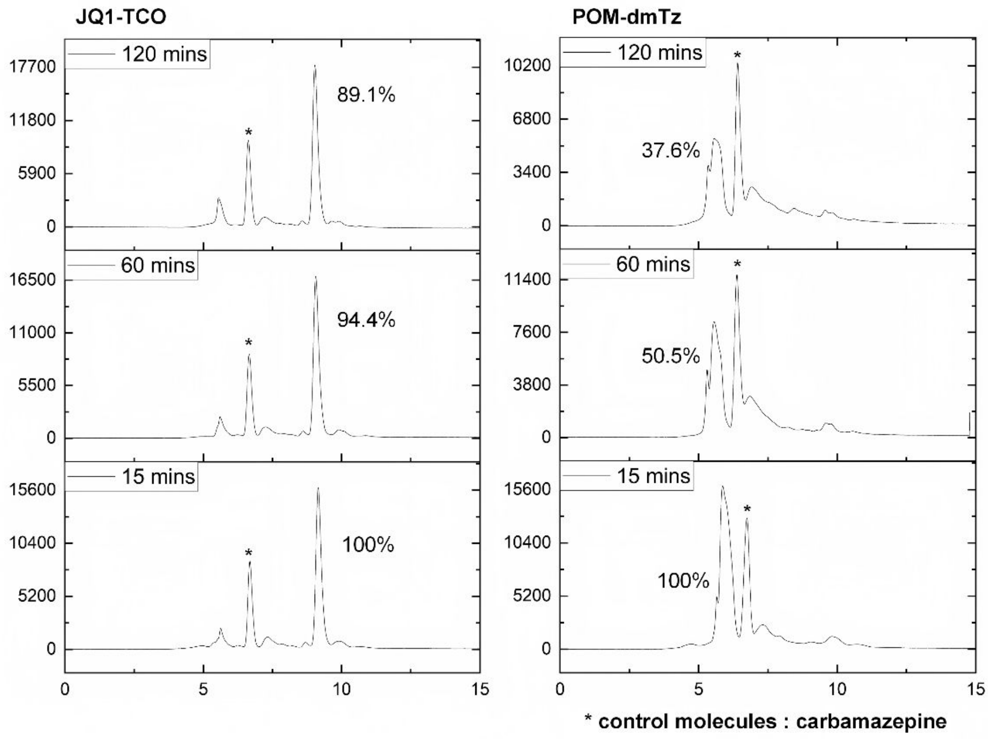

Figure S6. Stability test of compounds **JQ1-TCO** and **POM-dmTz** in mouse plasma, with carbamazepine as the control compound.

Experimental Section

Chemistry

Starting materials were purchased from BidePharm (Shanghai, China). All solvents and subsidiary materials were purchased from Guangzhou Chemical Reagent Factory (Guangzhou, Guangdong, China). The solvent ratios described in this section were volume ratios (v:v).

NMR spectra were measured using 400/600 MHz NMR system (spectrometer: Avance III; magnet: UltraShild 400/600, Bruker Biospin, Germany), chemical shifts were reported in ppm relative to TMS. MS spectra were measured using an ion trap mass spectrometer (LTQ-XL, Thermo Scientific, USA). HRMS spectra were measured using an orbitrap high-resolution mass spectrometer (Q-Exactive, Thermo Scientific).

General Method I (Deprotection):

Approximately 1 mmol of the reactant was dissolved in 10 mL of a mixed solvent of dichloromethane/trifluoroacetic acid (1:1). The reaction proceeded at room temperature with vigorous stirring for 2 h. Upon completion, the solvent was removed repeatedly under reduced pressure, followed by vacuum drying to afford the corresponding deprotected product.

General Method II (Amide Coupling):

0.25 mmol of reactant A (carboxylic acid), 0.375 mmol HATU (142.5 mg), and 0.75 mmol DIEA were dissolved in 5 mL of N,N-dimethylformamide and stirred at room temperature for 15 minutes. Then, 0.25 mmol of reactant B (amine) was added under stirring. The reaction continued for 16 h. Upon completion, the mixture was diluted with water and extracted with ethyl acetate. The combined organic layers were washed sequentially with deionized water and saturated brine, dried over

anhydrous magnesium sulfate, filtered, and concentrated under reduced pressure. The resulting crude product was purified by flash column chromatography (dichloromethane/methanol 95:5) and vacuum dried to afford the corresponding coupling product.

Synthesis of **Intermediate 2**

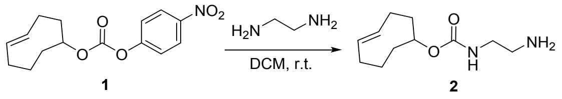

Compound 25 (1 mmol) was dissolved in anhydrous dichloromethane (2 mL). To a separate solution of **ethylenediamine** (3 mmol) in dichloromethane (1.5 mL), the solution of **25** was added **dropwise via an addition funnel over 30 min** at room temperature. After complete addition, the reaction mixture was stirred at rt for **2 h**, then quenched with **water (10 vol)**. The organic layer was washed sequentially with: Water (3 × 5 mL), Saturated brine (2 × 5 mL). Dried over **anhydrous Na_2SO_4**, concentrated *in vacuo* to afford the **crude product** as a **pale yellow solid** (85% yield), which was used directly in the next step without further purification.

Intermediate 2 ^{1}H NMR (400 MHz, Chloroform-*d*) δ 5.53 (dtd, *J* = 29.3, 15.9, 8.6 Hz, 2H), 5.09 (s, 1H), 4.31 (dd, *J* = 10.8, 5.8 Hz, 1H), 3.24 (s, 2H), 2.34 (dt, *J* = 9.8, 5.7 Hz, 3H), 1.94 (ddd, *J* = 32.2, 18.2, 12.1 Hz, 5H), 1.72 (dq, *J* = 19.7, 6.2, 5.5 Hz, 2H), 1.64 – 1.45 (m, 1H).

Synthesis of **JQ1-TCO**

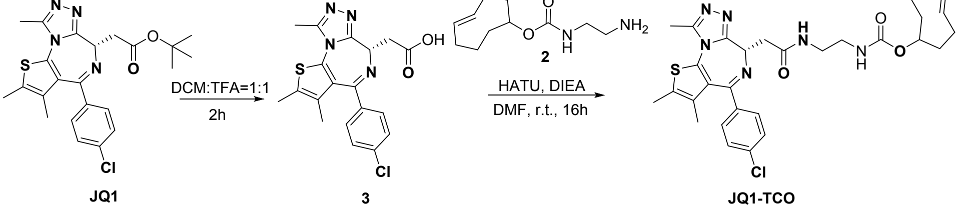

Deprotection of precursor **JQ1** (1 mmol) according to **General Procedure I** afforded a **colorless oil**, which underwent **condensation with Intermediate 2 under General Procedure II** to deliver **JQ1-TCO** as a **colorless oil** in **60-75% yield** (two-step sequence).

JQ1-TCO ^{1}H NMR (400 MHz, DMSO-d_6) δ 8.23 (t, *J* = 5.6 Hz, 1H), 7.48 (d, *J* = 8.5 Hz, 2H), 7.42 (d, *J* = 8.4 Hz, 2H), 6.93 (t, *J* = 5.5 Hz, 1H), 5.75 (s, 1H), 5.59 (m, 1H), 5.43 (m, 1H), 4.50 (dd, *J* = 8.0, 6.1 Hz, 1H), 4.22 (dd, *J* = 8.2, 4.8 Hz, 1H), 3.28 – 2.96 (m, 7H), 2.60 (s, 3H), 2.41 (s, 3H), 2.33 – 2.17 (m, 3H), 1.97 – 1.75 (m, 4H), 1.71 – 1.45 (m, 7H). ^{13}C NMR (101 MHz, DMSO) δ 170.30, 163.52, 156.34, 155.62, 150.32, 137.26, 135.71, 135.42, 133.02, 132.79, 131.20, 130.68, 130.35, 130.08, 128.98, 79.65, 55.42, 54.28, 41.19, 39.16, 38.69, 38.12, 34.26, 32.66, 31.11, 14.57, 13.19, 11.81. HRMS (ESI, *m/z*): calculated for $C_{30}H_{35}ClN_6O_3S$ [M + Na]$^+$ 617.2072, found 617.2070.

Synthesis of **JQ1-Tz**

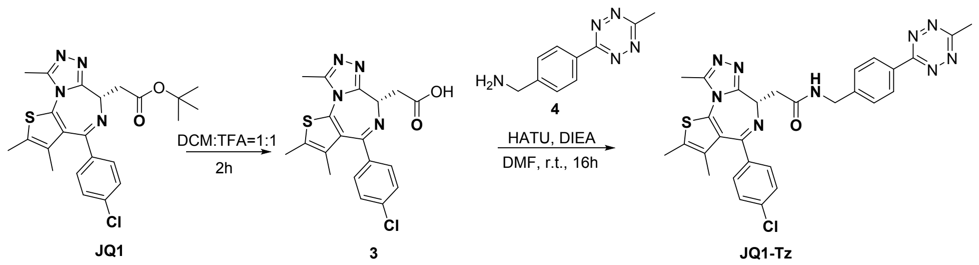

Deprotection of precursor **JQ1** (1 mmol) following **General Procedure I** afforded **intermediate 3** as a **colorless oil**, which subsequently underwent **condensation with compound 4 under General Procedure II** to provide **JQ1-Tz** as a **purple solid** in **55% yield** (over two steps).

JQ1-Tz ^{1}H NMR (400 MHz, DMSO-d_6) δ 8.92 (t, *J* = 6.1 Hz, 1H), 8.49 – 8.38 (m, 2H), 7.62 (d, *J* = 8.1 Hz, 2H), 7.52 – 7.34 (m, 4H), 4.64 – 4.38 (m, 3H), 3.17 (s, 1H), 3.01 (s, 3H), 2.62 (s, 3H), 2.41 (s, 3H), 1.63 (s, 3H). ^{13}C NMR (101 MHz, DMSO) δ 170.37, 167.56, 163.68, 163.62, 155.55, 150.35, 144.98, 137.18, 135.69, 132.77, 131.20, 130.84, 130.61, 130.31, 130.05, 128.88, 128.56, 127.84, 54.47, 42.34, 38.19, 21.30, 14.53, 13.15, 11.79. HRMS (ESI, *m/z*): calculated for $C_{44}H_{45}N_{13}O_{10}$ [M + H]$^+$ 915.43151, found 915.42975. HRMS (ESI, *m/z*): calculated for $C_{29}H_{26}ClN_9OS$ [M + H]$^+$ 584.1742, found 584.1739.

Synthesis of **JQ1-dTCO**：

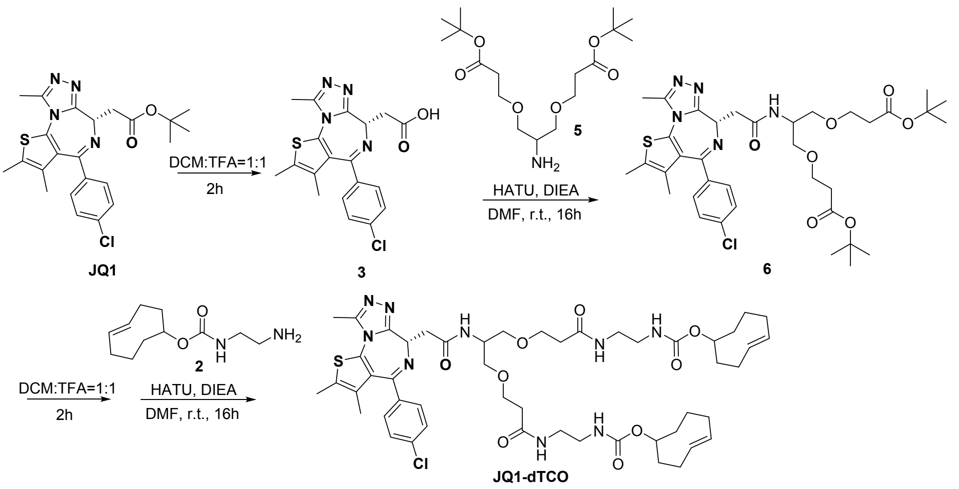

Deprotection of precursor JQ1 (1 mmol) under **General Procedure I** afforded **Compound 3** as a colorless oil, which was subjected to condensation with Precursor 5 via General Procedure II to yield **Intermediate 6**. Subsequent *deprotection* followed by condensation with **Intermediate 2** (2.0 equiv) delivered **JQ1-dTCO** as a colorless oil in 25% overall yield (over four steps).

JQ1-dTCO ^{1}H NMR (400 MHz, DMSO-d_6) δ 8.16 (d, *J* = 8.2 Hz, 1H), 7.91 (t, *J* = 5.6 Hz, 2H), 7.50 (d, *J* = 8.4 Hz, 2H), 7.43 (d, *J* = 8.3 Hz, 2H), 6.96 (t, *J* = 5.6 Hz, 2H), 5.56 (m, 2H), 5.42 (m, 2H), 4.55 – 4.43 (m, 2H), 4.27 – 4.07 (m, 4H), 4.01 (d, *J* = 7.5 Hz, 1H), 3.60 (dt, *J* = 16.6, 6.5 Hz, 5H), 3.45 – 3.36 (m, 6H), 3.17 (d, *J* = 5.4 Hz, 6H), 3.12 – 2.92 (m, 9H), 2.87 (d, *J* = 12.3 Hz, 3H), 2.60 (s, 3H), 2.41 (s, 3H), 2.29 (m, 12H), 1.86 (m, 10H), 1.72 – 1.44 (m, 11H). ^{13}C NMR (101 MHz, DMSO) δ 170.74, 170.02, 163.52, 156.31, 155.60, 150.32, 137.27, 135.74, 135.41, 133.01, 132.78, 131.20, 130.64, 130.34, 130.05, 128.97, 79.60, 69.91, 69.74, 67.47, 67.40, 63.29, 54.35, 49.10, 48.96, 41.16, 38.68, 36.53, 34.25, 32.66, 31.09, 14.59, 13.20, 11.82. HRMS (ESI, *m/z*): calculated for $C_{50}H_{48}ClN_9O_9S$ $[M + H]^+$ 1006.4622, found 1006.4619.

Synthesis of **EGFR-TCO**：

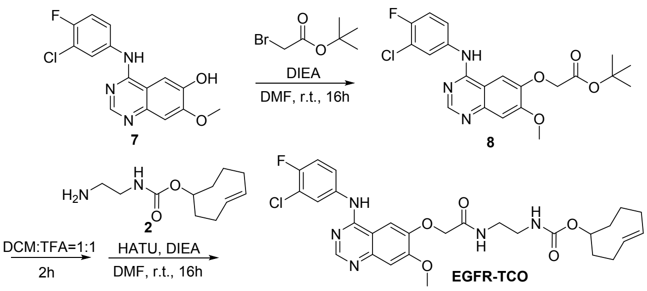

Compound **7** (1 mmol) was dissolved in anhydrous DMF, followed by addition of DIEA (3.0 equiv) and n-butyl bromoacetate (1 mmol). The reaction mixture was stirred at room temperature for 16 h. After completion, the reaction was quenched with water (50 mL). The crude product 8 was purified by flash column chromatography. Deprotection of 8 via General Procedure I, followed by condensation with compound 2 under General Procedure II, afforded the title compound EGFR-TCO as a colorless oil in 65% yield. (over two steps)

EGFR-TCO ^{1}H NMR (400 MHz, DMSO-d_6) δ 9.52 (s, 1H), 8.52 (s, 1H), 8.09 (dd, *J* = 6.9, 2.6 Hz, 1H), 7.92 (t, *J* = 5.8 Hz, 1H), 7.84 (s, 1H), 7.77 (dd, *J* = 9.2, 3.7 Hz, 1H), 7.44 (t, *J* = 9.1 Hz, 1H), 7.24 (s, 1H), 7.03 (s, 1H), 5.75 (s, 1H), 5.53 (m, 1H), 5.35 (m, 1H), 4.66 (s, 2H), 4.16 (t, *J* = 6.5 Hz, 1H), 3.98 (s, 3H), 3.24 (d, *J* = 6.1 Hz, 2H), 3.11 (d, *J* = 6.3 Hz, 2H), 2.19 (ddt, *J* = 18.2, 10.9, 5.8 Hz, 3H), 1.93 – 1.73 (m, 4H), 1.67 – 1.39 (m, 3H), 1.28 – 1.13 (m, 1H). ^{13}C NMR (101 MHz, DMSO) δ 167.78, 156.59, 156.46, 154.95, 154.89, 153.47, 152.47, 147.91, 147.77, 137.15, 137.12, 135.32, 132.92, 123.97, 122.80, 122.74, 119.37, 119.19, 117.08, 116.87, 109.03, 108.04, 104.31, 79.65, 68.61, 56.44, 55.36, 41.09, 38.59, 34.17, 32.57, 31.02. HRMS (ESI, *m/z*): calculated for $C_{28}H_{31}ClFN_5O_5$ $[M + H]^+$ 572.2071, found 572.2070.

Synthesis of **EGFR-Tz**：

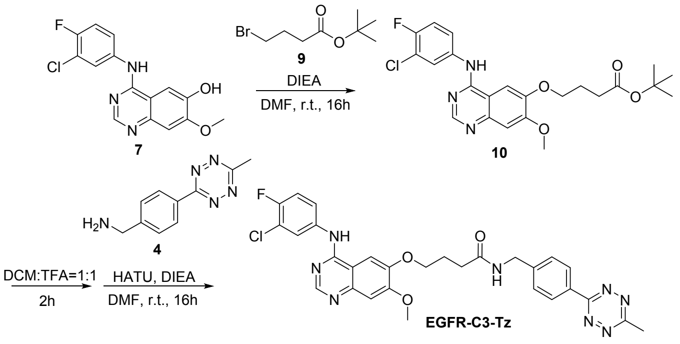

Compound 7 (1 mmol) was dissolved in anhydrous DMF. Compound 9 (1 mmol) and DIEA (3.0 equiv) were added, and the reaction mixture was stirred at room temperature for 16 h. After completion, the reaction was quenched with water (50 mL). The crude product 10 was isolated by flash column chromatography. Subsequent deprotection via General Procedure I, followed by condensation with compound 4 under General Procedure II, afforded the target molecule EGFR-Tz as a purple oil in 60% yield (over two steps).

EGFR-Tz ^{1}H NMR (400 MHz, DMSO-d_6) δ 9.55 (s, 1H), 8.62 (t, J = 6.0 Hz, 1H), 8.50 (s, 1H), 8.35 (d, J = 8.2 Hz, 3H), 8.12 (dd, J = 6.9, 2.6 Hz, 1H), 7.88 – 7.74 (m, 3H), 7.51 (d, J = 8.1 Hz, 3H), 7.42 (t, J = 9.1 Hz, 1H), 7.21 (s, 1H), 4.43 (d, J = 5.9 Hz, 3H), 4.23 – 4.07 (m, 4H), 3.95 (s, 4H), 3.17 (d, J = 5.2 Hz, 4H), 2.98 (s, 4H), 2.47 (d, J = 7.1 Hz, 2H), 2.14 (t, J = 6.7 Hz, 3H). ^{13}C NMR (101 MHz, DMSO) δ 172.28, 167.52, 163.61, 156.46, 154.95, 154.79, 153.10, 152.38, 148.80, 147.41, 144.93, 137.32, 130.81, 128.50, 127.89, 123.88, 122.73, 122.66, 119.31, 119.13, 117.07, 116.85, 109.25, 107.78, 102.95, 68.65, 56.38, 49.10, 42.41, 32.02, 25.08, 21.32. HRMS (ESI, *m/z*): calculated for $C_{29}H_{26}ClFN_8O_3$ [M + H]$^+$ 589.1873, found 589.1874.

Synthesis of **ALK-TCO module**：

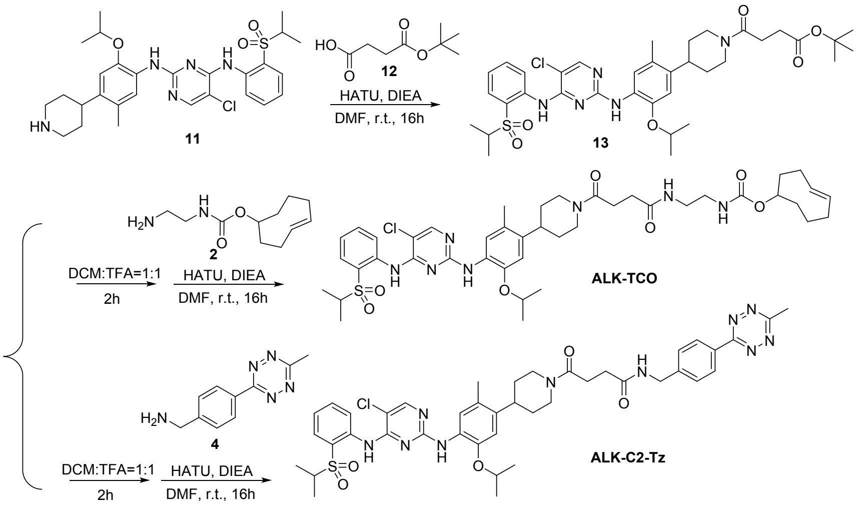

Compound **11** (1 mmol) was dissolved in anhydrous DMF and condensed with **compound 12** via **General Procedure II** at room temperature for 16 h to afford intermediate **13**. After deprotection of **13** , sequential condensations with **compound 2** and **compound 4** under **General Procedure II** yielded the final products **ALK-TCO** (as a pale yellow oil, 55% yield) and **ALK-C2-Tz** (as a purple oil, 50% yield).

ALK-TCO ^{1}H NMR (400 MHz, Chloroform-*d*) δ 8.54 (d, *J* = 8.3 Hz, 1H), 8.10 (s, 1H), 7.98 (d, *J* = 7.9 Hz, 1H), 7.82 (s, 1H), 7.60 (t, *J* = 8.5 Hz, 1H), 7.36 (t, *J* = 8.0 Hz, 1H), 6.76 (d, *J* = 2.4 Hz, 1H), 6.46 (s, 1H), 5.56 (q, *J* = 10.5, 8.8 Hz, 1H), 5.30 (s, 1H), 4.82 (d, *J* = 13.3 Hz, 1H), 4.69 – 4.55 (m, 1H), 4.07 (d, *J* = 13.6 Hz, 1H), 3.49 – 3.12 (m, 6H), 2.94 (s, 1H), 2.74 (dd, *J* = 30.5, 8.1 Hz, 3H), 2.56 (d, *J* = 6.4 Hz, 2H), 2.37 (t, *J* = 6.4 Hz, 3H), 2.21 (d, *J* = 2.4 Hz, 4H), 2.10 – 2.01 (m, 2H), 1.88 – 1.69 (m, 7H), 1.41 (p, *J* = 2.9 Hz, 14H). ^{13}C NMR (101 MHz, $CDCl_3$) δ 173.42, 170.26, 134.94, 134.73, 133.02, 131.55, 123.91, 111.12, 71.88, 55.97, 46.21, 42.79, 41.22, 38.73, 38.44, 34.33, 33.08, 32.57, 32.35, 31.63, 31.48, 31.01, 30.24, 29.73, 28.94, 22.22, 18.95, 15.39. HRMS (ESI, *m/z*): calculated for $C_{43}H_{58}ClN_7O_7S$ [M + H]$^+$ 852.3880, found 852.3874.

ALK-C2-Tz ^{1}H NMR (400 MHz, DMSO-*d*$_6$) δ 9.46 (s, 1H), 8.55 – 8.36 (m, 5H), 8.25 (s, 1H), 8.04 (s, 1H), 7.84 (dd, *J* = 8.0, 1.6 Hz, 1H), 7.68 – 7.59 (m, 1H), 7.59 – 7.50 (m, 3H), 7.41 – 7.32 (m, 1H),

6.83 (s, 1H), 4.67 – 4.51 (m, 2H), 4.42 (d, J = 6.0 Hz, 2H), 4.15 – 3.98 (m, 2H), 3.44 (p, J = 6.8 Hz, 1H), 3.18 (d, J = 5.2 Hz, 3H), 2.99 (s, 4H), 2.91 (d, J = 9.7 Hz, 2H), 2.77 – 2.61 (m, 7H), 2.16 (s, 3H), 1.18 – 1.12 (m, 11H). ^{13}C NMR (101 MHz, DMSO) δ 172.31, 170.06, 167.53, 163.67, 158.46, 155.87, 155.31, 147.09, 145.07, 139.43, 138.48, 135.32, 131.40, 130.74, 128.47, 127.82, 127.29, 126.89, 124.91, 124.24, 124.14, 124.06, 112.26, 104.72, 71.08, 60.23, 55.39, 55.29, 49.07, 46.09, 42.47, 42.30, 38.72, 38.16, 33.01, 32.54, 31.02, 28.29, 22.35, 21.29, 18.91, 15.32. HRMS (ESI, *m/z*): calculated for $C_{42}H_{49}ClN_{10}O_5S$ $[M + H]^+$ 841.3369, found 841.3364.

Synthesis of **POM-d series**：

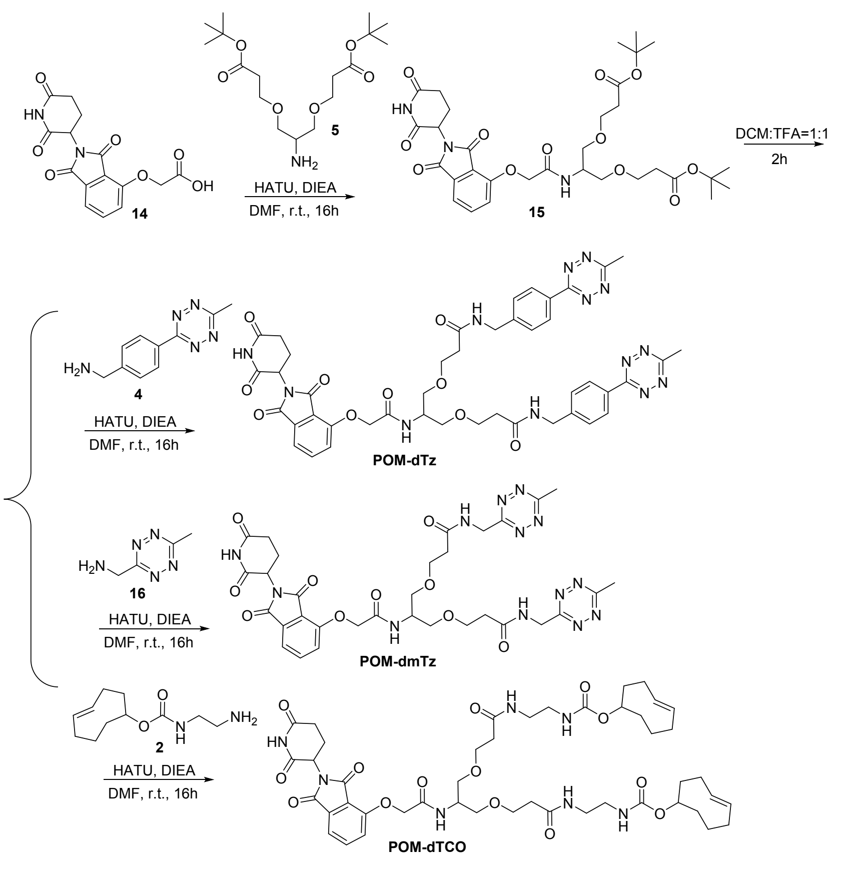

Compound **14** (1 mmol) was dissolved in anhydrous DMF and condensed with **compound 5** under **General Procedure II** to afford intermediate **15**. Subsequent deprotection via **General Procedure I**, followed by separate condensations with **compounds 4, 16, and 2** (each under **General Procedure**

II), yielded the final products:**POM-dTz** (as a pale violet powder, 25% yield),**POM-dmTz** (as a purple powder, 15% yield),**POM-dTCO** (as a white solid, 45% yield)

POM-dTz ^{1}H NMR (400 MHz, Chloroform-*d*) δ 9.41 (s, 1H), 8.37 (dd, *J* = 13.4, 8.0 Hz, 4H), 7.59 (t, *J* = 8.1 Hz, 2H), 7.38 (m, 6H), 7.00 (d, *J* = 8.4 Hz, 1H), 4.93 (dd, *J* = 12.0, 5.6 Hz, 1H), 4.63 – 4.23 (m, 7H), 3.71 (q, *J* = 6.0 Hz, 4H), 3.62 – 3.36 (m, 4H), 3.06 (s, 6H), 2.76 (ddd, *J* = 36.9, 17.1, 9.2 Hz, 5H), 2.60 – 2.41 (m, 4H), 2.19 – 2.08 (m, 1H). ^{13}C NMR (101 MHz, $CDCl_3$) δ 171.99, 171.87, 171.58, 168.90, 167.25, 166.70, 166.46, 166.13, 163.72, 163.67, 153.96, 143.70, 143.60, 136.94, 133.40, 130.62, 130.52, 128.20, 128.10, 128.02, 127.96, 118.93, 117.57, 117.01, 69.58, 69.41, 67.41, 67.32, 67.28, 53.52, 49.26, 48.49, 43.06, 42.96, 36.90, 36.80, 31.30, 22.75, 21.15. HRMS (ESI, *m/z*): calculated for $C_{44}H_{45}N_{13}O_{10}$ $[M + Na]^+$ 938.3305, found 938.3298.

POM-dmTz ^{1}H NMR (400 MHz, DMSO) δ 11.09 (s, 1H), 8.74 (td, J = 5.8, 2.0 Hz, 2H), 7.95 – 7.75 (m, 2H), 7.49 (d, J = 7.2 Hz, 1H), 7.38 (d, J = 8.5 Hz, 1H), 5.11 (dd, J = 12.9, 5.4 Hz, 1H), 4.87 – 4.69 (m, 8H), 4.08 – 3.96 (m, 1H), 3.68 – 3.52 (m, 8H), 3.41 (dd, J = 5.8, 2.8 Hz, 6H), 2.95 (s, 8H), 2.51 (s, 6H), 2.44 (td, J = 6.5, 1.8 Hz, 5H). ^{13}C NMR (101 MHz, DMSO) δ 173.26, 171.24, 170.39, 168.22, 167.30, 137.42, 120.85, 116.52, 69.53, 67.25, 54.06, 42.36, 36.19, 22.49, 21.24, 18.58, 17.24, 12.94. HRMS (ESI, *m/z*): calculated for $C_{32}H_{37}N_{13}O_{10}$ $[M + H]^+$ 764.2859, found 764.2852.

POM-dTCO ^{1}H NMR (400 MHz, DMSO-d_6) δ 11.12 (s, 1H), 7.87 (ddd, *J* = 32.9, 17.8, 8.1 Hz, 5H), 7.50 (d, *J* = 7.2 Hz, 1H), 7.39 (d, *J* = 8.5 Hz, 1H), 6.95 (t, *J* = 5.6 Hz, 2H), 5.57 (ddd, *J* = 15.0, 10.3, 4.2 Hz, 2H), 5.49 – 5.39 (m, 2H), 5.13 (dd, *J* = 12.9, 5.4 Hz, 1H), 4.80 (s, 2H), 4.19 (dt, *J* = 9.9, 5.1 Hz, 2H), 4.04 (dt, *J* = 8.1, 5.4 Hz, 1H), 3.70 – 3.54 (m, 6H), 3.43 (s, 4H), 3.02 (dq, *J* = 33.1, 7.8, 7.3 Hz, 11H), 2.93 – 2.79 (m, 3H), 2.68 – 2.55 (m, 2H), 2.26 (ddd, *J* = 20.3, 15.3, 8.7 Hz, 13H), 2.05 (m, 2H), 1.99 – 1.76 (m, 11H), 1.57 (m, 8H). ^{13}C NMR (101 MHz, DMSO) δ 173.29, 170.69, 170.66,

170.41, 167.25, 167.20, 165.97, 156.30, 155.50, 137.41, 135.41, 133.52, 133.01, 120.81, 117.18, 116.50, 79.61, 69.49, 67.82, 67.43, 55.42, 49.30, 48.73, 41.16, 38.68, 36.48, 34.25, 32.66, 31.46, 31.09, 22.50. HRMS (ESI, *m/z*): calculated for $C_{46}H_{63}N_7O_{14}$ [M + Na]$^+$ 960.4506, found 960.4314.

Synthesis of **POM-STz**：

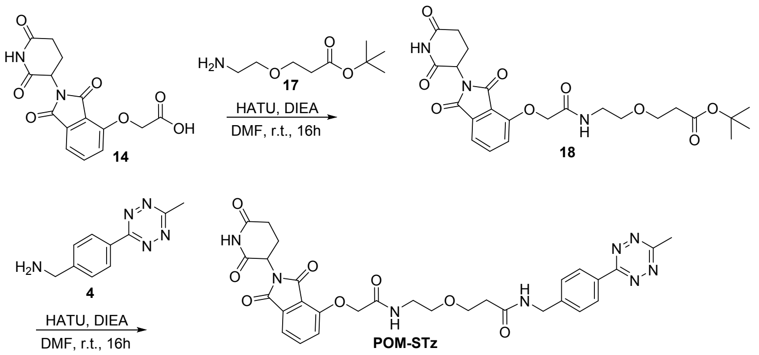

Compound **14** (1 mmol) was dissolved in anhydrous DMF and condensed with **compound 17** under **General Procedure I** to afford intermediate **18**. After purification, **18** was deprotected via **General Procedure I**. Subsequent condensation with **compound 4** under **General Procedure II** yielded the target compound **POM-STz** as a purple powder in **66% yield**.

POM-STz ^{1}H NMR (400 MHz, DMSO-d_6) δ 11.11 (s, 1H), 8.53 – 8.44 (m, 1H), 8.44 – 8.35 (m, 2H), 8.03 (t, *J* = 5.6 Hz, 1H), 7.80 (dd, *J* = 8.5, 7.3 Hz, 1H), 7.50 (dd, *J* = 16.0, 7.7 Hz, 3H), 7.38 (d, *J* = 8.5 Hz, 1H), 5.12 (dd, *J* = 12.9, 5.4 Hz, 1H), 4.78 (s, 2H), 4.41 (d, *J* = 5.9 Hz, 2H), 3.68 (t, *J* = 6.4 Hz, 2H), 3.48 (t, *J* = 5.8 Hz, 2H), 2.94 – 2.77 (m, 1H), 2.60 (dt, *J* = 14.0, 2.9 Hz, 1H), 2.55 – 2.42 (m, 4H), 2.13 – 1.98 (m, 1H). ^{13}C NMR (101 MHz, DMSO) δ 173.27, 170.90, 170.38, 167.56, 167.44, 167.23, 165.96, 163.67, 155.50, 144.87, 137.43, 133.52, 130.81, 128.45, 127.88, 120.88, 117.28, 116.54, 69.05, 68.05, 67.17, 49.32, 42.34, 38.90, 36.60, 31.46, 22.51, 21.32. HRMS (ESI, *m/z*): calculated for $C_{30}H_{30}N_8O_8$ [M + H]$^+$ 631.2253, found 631.2258.

Synthesis of **POM-C1-Tz**：

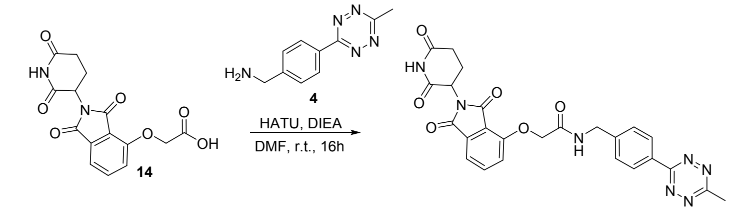

Compound 14 was condensed with **compound 4** under **General Procedure I** in anhydrous DMF to afford **POM-C1-Tz** as a purple powder in **65% yield**.

POM-C1-Tz ^{1}H NMR (400 MHz, DMSO-d_6) δ 11.13 (s, 1H), 8.69 (t, *J* = 6.1 Hz, 1H), 8.42 (d, *J* = 8.1 Hz, 2H), 7.84 (dd, *J* = 8.4, 7.3 Hz, 1H), 7.61 – 7.38 (m, 4H), 5.13 (dd, *J* = 13.0, 5.3 Hz, 1H), 4.93 (s, 2H), 4.51 (d, *J* = 5.9 Hz, 2H), 2.94 – 2.82 (m, 1H), 2.76 – 2.56 (m, 1H), 2.15 – 2.00 (m, 1H). ^{13}C NMR (101 MHz, DMSO) δ 173.29, 170.39, 167.77, 167.60, 167.24, 165.91, 163.64, 155.60, 144.24, 137.43, 133.59, 131.01, 128.60, 127.94, 120.86, 117.36, 116.59, 68.15, 49.29, 42.27, 31.45, 22.51, 21.34. HRMS (ESI, *m/z*): calculated for $C_{25}H_{21}N_7O_6$ [M + H]$^+$ 516.1626, found 516.1628.

Synthesis of **VH032-dTCO**：

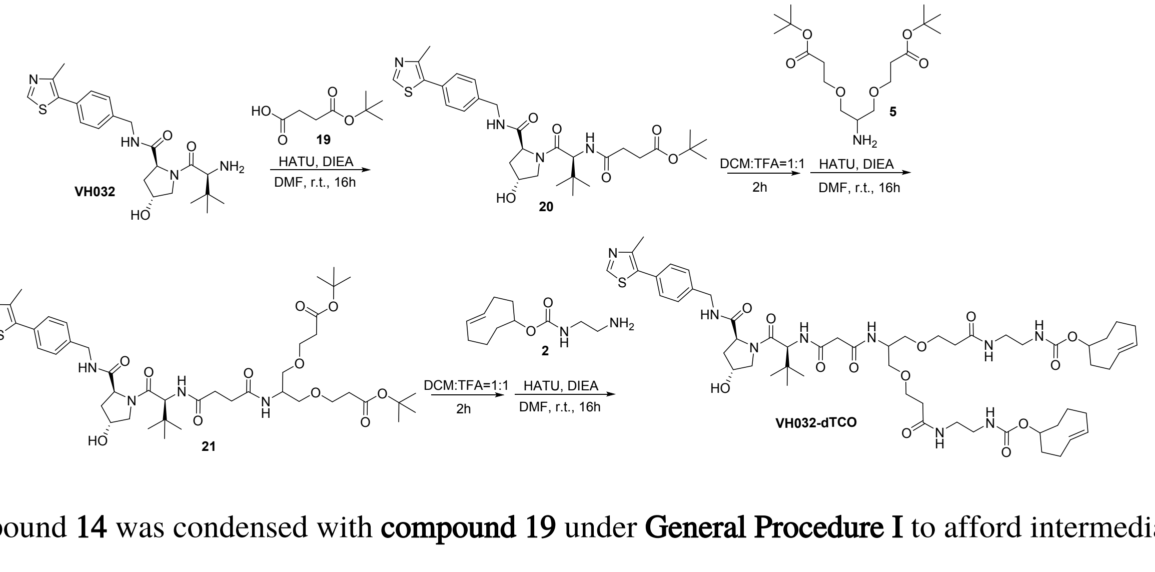

Compound **14** was condensed with **compound 19** under **General Procedure I** to afford intermediate **20**. Deprotection of **20**, followed by condensation with **compound 5**, yielded intermediate **21**. Subsequent deprotection of **21** and condensation with **2 equiv of compound 2** afforded **VH032-dTCO** as a colorless oil in **15% overall yield**.

VH032-dTCO ^{1}H NMR (400 MHz, DMSO-d_6) δ 9.00 (s, 1H), 8.57 (t, *J* = 6.1 Hz, 1H), 7.90 (t, *J* = 6.7 Hz, 3H), 7.72 (d, *J* = 8.0 Hz, 1H), 7.50 – 7.36 (m, 4H), 6.95 (t, *J* = 5.7 Hz, 1H), 5.60 (m, 2H), 5.43 (m, 1H), 4.53 (d, *J* = 9.3 Hz, 2H), 4.48 – 4.37 (m, 2H), 4.35 (s, 1H), 4.21 (td, *J* = 13.6, 11.6, 5.3 Hz, 3H), 3.92 (q, *J* = 7.0, 6.5 Hz, 2H), 3.32 (qt, *J* = 9.2, 6.0, 5.3 Hz, 5H), 3.03 (dq, *J* = 32.9, 6.9 Hz, 8H), 2.45 (s, 4H), 1.88 (tdd, *J* = 18.1, 9.7, 5.3 Hz, 8H), 1.74 – 1.40 (m, 8H), 1.25 (d, *J* = 7.7 Hz, 3H), 0.94 (s, 10H). ^{13}C NMR (101 MHz, DMSO) δ 172.39, 171.78, 170.71, 170.05, 156.29, 151.93, 148.15, 139.99, 135.38, 132.99, 131.68, 130.11, 129.12, 127.92, 79.60, 69.68, 69.35, 67.36, 59.18, 56.89, 56.75, 48.84, 42.15, 41.15, 38.65, 36.49, 35.81, 34.22, 32.63, 31.07, 26.83, 16.38. HRMS (ESI, *m/z*): calculated for $C_{57}H_{85}N_9O_{13}S$ [M + H]$^+$ 1136.6060, found 1136.6068.

Synthesis of **VH032-C2-Tz**：

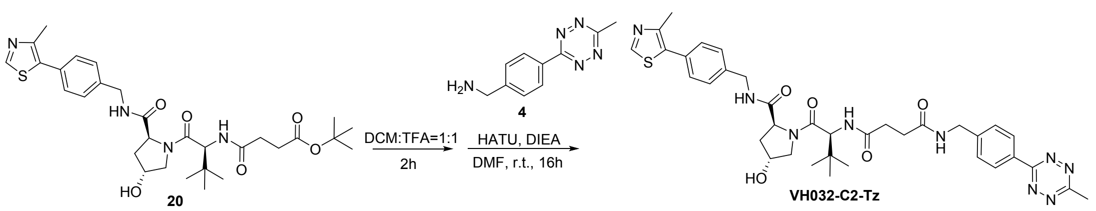

Deprotection of compound 14, followed by condensation with **compound 4** under **General Procedure I**, afforded **VH032-C2-Tz** as a purple powder in **70% overall yield**.

VH032-C2-Tz ^{1}H NMR (400 MHz, Chloroform-*d*) δ 8.84 (s, 1H), 8.47 (d, *J* = 8.1 Hz, 2H), 7.67 (t, *J* = 5.9 Hz, 1H), 7.43 (d, *J* = 8.0 Hz, 2H), 7.32 (s, 5H), 7.21 – 7.03 (m, 2H), 4.71 (t, *J* = 8.2 Hz, 1H), 4.61 – 4.38 (m, 6H), 4.34 (dd, *J* = 15.3, 5.3 Hz, 1H), 3.95 (d, *J* = 11.3 Hz, 1H), 3.61 (dd, *J* = 11.4, 3.4 Hz, 1H), 3.08 (s, 3H), 2.66 – 2.44 (m, 8H), 2.33 (m, 1H), 2.15 (dd, *J* = 13.4, 7.9 Hz, 1H), 0.97 (s, 9H). ^{13}C NMR (101 MHz, $CDCl_3$) δ 172.69, 172.65, 171.49, 171.28, 167.30, 163.74, 143.17, 138.67, 130.78, 130.19, 129.36, 128.17, 128.15, 128.04, 70.11, 63.76, 58.76, 57.84, 56.99, 53.45, 43.17, 43.08, 36.69, 35.36, 31.22, 31.04, 26.46, 21.17, 15.66. HRMS (ESI, *m/z*): calculated for $C_{36}H_{43}N_9O_5S$ [M + H]$^+$ 714.3181, found 714.3171.

Synthesis of **GW-Tz-1**：

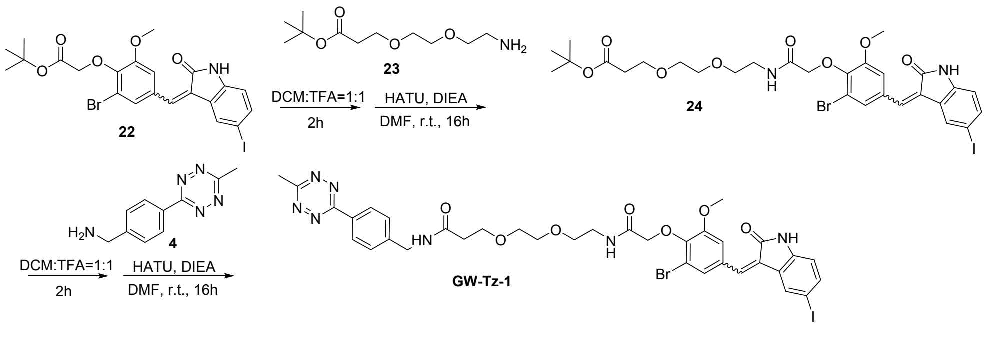

Deprotection of compound 22, followed by condensation with **compound 23** under **General Procedure I**, afforded intermediate **24**. Subsequent deprotection of **24** and condensation with **compound 4** yielded **GW-Tz-1** as a purple powder in **13% overall yield**.

GW-Tz-1 ^{1}H NMR (400 MHz, DMSO-d_6) δ 10.75 (d, *J* = 7.4 Hz, 1H), 8.49 (s, 1H), 8.46 – 8.35 (m, 2H), 8.02 – 7.86 (m, 1H), 7.60 – 7.47 (m, 3H), 7.51 – 7.44 (m, 1H), 7.44 – 7.31 (m, 1H), 7.18 (t, *J* = 2.2 Hz, 1H), 6.72 (dd, *J* = 17.4, 8.2 Hz, 1H), 4.47 (d, *J* = 3.1 Hz, 2H), 4.40 (d, *J* = 5.9 Hz, 1H), 3.87 (d, *J* = 5.0 Hz, 2H), 3.71 (t, *J* = 6.3 Hz, 2H), 3.56 – 3.48 (m, 2H), 2.97 (d, *J* = 5.4 Hz, 2H), 2.45 (d, *J* = 6.3 Hz, 1H), 2.01 (t, *J* = 7.5 Hz, 1H), 1.97 (s, 1H), 1.57 – 1.43 (m, 1H). ^{13}C NMR (101 MHz, DMSO) δ 170.91, 167.91, 167.53, 163.66, 144.87, 140.70, 138.79, 130.80, 128.46, 127.86, 116.74, 71.94, 70.09, 69.41, 67.40, 56.71, 42.34, 40.67, 40.46, 40.25, 40.04, 39.83, 39.62, 39.41, 38.78, 36.71, 31.75, 30.35, 21.31. HRMS (ESI, *m/z*): calculated for $C_{35}H_{35}BrIN_7O_7$ $[M + H]^+$ 872.0899, found 872.0891.

Synthesis of **AN2-C2-Tz**：

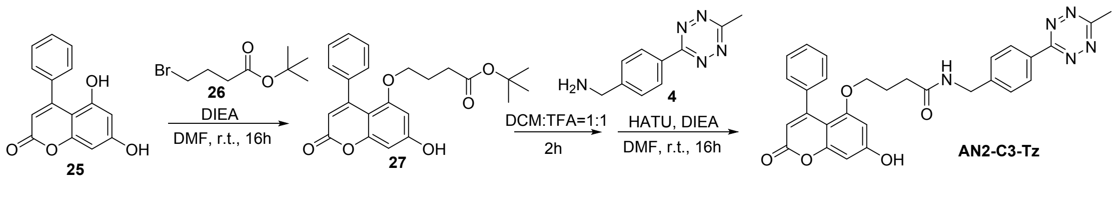

A solution of compound **25** (1 mmol) in DMF was treated with DIEA (3 equiv) and compound **26** (1 mmol). The reaction mixture was stirred at room temperature for 16 h, quenched with water (50 mL), and purified by flash column chromatography to afford crude intermediate **27**. Subsequent

deprotection under General Procedure I, followed by condensation with compound 4 under **General Procedure II**, yielded **AN2-C3-Tz** as a purple oil in 60% yield (over two steps from **25**).

AN2-C3-Tz ^{1}H NMR (400 MHz, DMSO-d_6) δ 10.67 (s, 1H), 8.44 (d, J = 8.0 Hz, 2H), 8.26 (d, J = 5.9 Hz, 1H), 7.57 – 7.25 (m, 9H), 6.41 (d, J = 2.1 Hz, 1H), 6.25 (d, J = 2.2 Hz, 1H), 5.80 (s, 1H), 4.36 (d, J = 5.8 Hz, 2H), 3.67 (m, 2H), 3.00 (s, 3H), 2.51 (m, 8H), 1.78 (t, J = 7.4 Hz, 2H), 1.28 (m, 3H). ^{13}C NMR (101 MHz, DMSO) δ 171.93, 167.53, 163.67, 162.62, 160.10, 158.13, 157.06, 156.10, 145.02, 140.35, 130.85, 128.53, 128.12, 127.98, 127.92, 127.88, 127.42, 111.25, 101.96, 96.97, 96.01, 68.02, 42.31, 38.72, 31.72, 24.34, 21.29. HRMS (ESI, *m/z*): calculated for $C_{29}H_{25}N_5O_5$ $[M + H]^+$ 524.1928, found 524.1929.

The full NMR and MS spectra of the synthesized degrader compounds were shown in **Appendix A** and **B**, respectively. The degrader compounds were dissolved in DMSO as 10 mM stock solution and stored at -20°C, freeze-thaw cycle was avoided. Inhibitors for bioassays were purchased from MedChemExpress.

Cell culture and drug treatment

The breast cancer cells and leukemia cells used were purchased from Wuhan Purdue Life Technology Co., Ltd. All cell lines were tested for mycoplasma to ensure no contamination. The cells were cultured in a complete medium containing 10-15% fetal bovine serum (FBS, cell culture reagents were purchased from Gibco Company in the United States), 100 U/mL penicillin and 100 μg/mL streptomycin: breast cancer cells were incubated in a 37 °C, 5% CO_2 incubator using DMEM medium, and PC9 cells were incubated in 1640 medium.

Direct Monitoring of the Click Reaction

The fluorescence spectrum of POM-dTz was measured using a Hitachi F4500. Three groups of PC9 cells were prepared and treated with DMSO, 10 μM POM-dTz, and a combination of 10 μM POM-dTz and 20 μM JQ1-TCO, respectively. Observations were carried out using confocal laser scanning microscopy (excitation at 325 nm, emission at 410 nm).

Immunoblot analysis

The total protein of cultured cells was extracted using a strong SDS lysis buffer (Beyotime, Cat. No. P0013G) premixed with a protease inhibitor cocktail. The lysate was then processed by low-temperature ultrasound lysis (30 s, 0.5 s on/0.5 s off) and centrifugation (12000×g, 10 min). The supernatant was quantified and adjusted using a bicinchoninic acid (BCA) method and then boiled in an SDS loading buffer. The protein samples were electrophoretically separated by 15% polyacrylamide gel (PAG) and subsequently transferred onto a 0.45 μm polyvinylidene difluoride (PVDF) microporous membrane (Millipore). The membrane was blocked in tris-buffered saline with tween-20 (TBST) with 5% nonfat dry milk at room temperature for 1 h and then incubated overnight with corresponding primary antibodies at 4℃. After 3 washes with TBST, the membrane was incubated with HRP-conjugated secondary antibodies at room temperature for 1 h. After another 3 washes with TBST, the membrane was incubated with an enzymatic chemiluminescence (ECL) reagent, and the signal was detected instantly by a chemiluminescence imager (JP-K600, Jiapeng, Shanghai, China). The signal values were quantified by ImageJ software. The expression level of a target protein was determined by the ratio of its signal value to GAPDH's. All antibodies used for immunodetection (including immunoblotting, dot blotting, co-IP, IHC, and IF) were listed below:

BRD4 antibody (Cell Signaling Technology, item # 13440, used for immunoblotting IF, Co-IP), EGFR antibody (Cell Signaling Technology, item # 4267, used for immunoblotting IF, Co-IP), ALK

antibody (Cell Signaling Technology, item # 3633), Anti GAPDH antibody [6C5] (Abcam UK, item number ab8245), Cy3 labeled goat anti rabbit IgG (Abcam, UK, item number ab6939), HRP labeled anti rabbit IgG (Cell Signaling Technology, item # 7074, USA), IPKine HRP labeled mouse anti rabbit IgG (Abbkine, Hubei, China, item number A25022)

IF assay (confocal LSM and FCM)

After drug treatment, the cells were washed with PBS, fixed with 4% paraformaldehyde (PFA) for 30 minutes, permeabilized with cold methanol for 10 minutes, and blocked with 5% bovine serum albumin (BSA) for 1 hour. After overnight incubation of the primary antibody, Alexa Fluor 488/Cy3 labeled secondary antibody was incubated at room temperature for 4 hours. DAPI (Shanghai Biyun Tian Company, product number C1005) was counterstained and observed using confocal laser scanning microscopy (Zeiss, Germany, LSM 710 NLO). Images were analyzed using ImageJ software (Ver. 1.52s).

Annexin V/PI dual staining method for detecting cell apoptosis

Wash the cells twice with pre cooled phosphate buffered saline (PBS) (centrifuge at 2000 × g for 5 minutes), and gently digest the adherent cells with EDTA free trypsin to avoid membrane damage. After resuspension, the cells were transferred to a system containing 1 × binding buffer (concentration controlled at 1-5 × 10^{6}/mL), and Ca^{2+} - dependent phospholipid binding probe (Annexin V-FITC 5 μ L) and nucleic acid fluorescent dye (PI 5 μ L) were added for double labeling. The reaction was carried out at room temperature in the dark for 5-15 minutes. The experiment requires synchronous setting of blank control (no dye) and single staining compensation control (Annexin V or PI staining separately). All samples need to be detected by flow cytometry within 30 minutes after staining, using FITC (Ex 488 nm/Im 530 nm) and PI (Ex 535 nm/Im 615 nm) dual channel analysis. This process can

distinguish live cells (double negative), early apoptotic cells (Annexin V $^{+}$/PI $^{-}$), and late apoptotic/necrotic cells (double positive) through differences in fluorescence signals.

Compound mediated cell cloning experiment

Cells in the exponential growth phase were digested with trypsin EDTA and seeded at low density (500-1000 cells/well) in a six well plate. After the cells adhered to the wall, fresh medium containing gradient concentration compounds was replaced, and fluid was replenished every 48 hours to maintain effective concentration. After 7-14 days of cultivation, discard the culture medium, gently rinse with pre cooled PBS, fix with 4% paraformaldehyde at room temperature for 15 minutes, stain with 0.1% crystal violet dye (containing 20% methanol) at room temperature in the dark for 20 minutes, and rinse with double distilled water until the background is transparent. Use a digital camera to take photos and record.

Ternary complex and ubiquitination level assays

NanoBRET™ CRBN ternary complex formation and ubiquitination levels were quantitatively analyzed using PROMEGA's ND2720 and ND2690 assay kits following the manufacturer's protocols. Time-course measurements of fluorescence intensity were performed, with the acquired data subsequently converted to nanoBRET values for quantitative comparison.

DIA quantitative proteomic profiling

The experimental steps and parameters for DIA relative quantitative proteomics were provided on page s5 of the supporting information.

Bioinformatic analysis

The raw sequencing data is subjected to bioinformatics quality control processes (such as automated preprocessing modules) to eliminate low-quality sequences and adapter contamination; Complete

sequence localization through reference genome mapping tools and construct gene expression matrices based on standardized transcriptome annotation files; Using differential expression analysis algorithm to screen significant differentially expressed genes between the experimental group and the control group, and synchronously performing functional enrichment analysis (such as molecular pathway annotation and gene ontology analysis) to reveal biological process characteristics; Finally, the results were presented using bioinformatics (https://www.bioinformatics.com.cn) to implement multidimensional data visualization techniques such as heatmaps and volcano maps.

Cell viability assay (CCK-8 assay)

Cells were transplanted onto a 96-well plate (1×10^6/mL). After an overnight incubation, cells were treated with the compounds of interest. After the treatment, the culture medium was removed, and cells were co-incubated with fresh culture medium containing 10% CCK-8 reagent (Beyotime, Cat. No. C0009S) at 37℃ for 4 h. The optical absorbance at 460 nm (OD_{460}) was subsequently measured using a microplate reader (Synergy H1, BioTek).

Animal experiment procedures

General methods

General method: All animal experiments involved in this study were approved by the Animal Control Committee and Laboratory Animal Ethics Committee (approval number, 2024040) of South China University of Technology, Guangzhou, China. And the experimental procedures followed international standards for animal welfare throughout the entire process. Female NOD-SCID mice (60-70 days old, weighing 22-24 g) were purchased from Guangdong Medical Experimental Animal Center in China and housed in SPF environment (24 ± 2 ℃, 12 hours of light dark cycle).

PK characteristic analysis

This pharmacokinetic study protocol used SPF grade male SD rats (6-8 weeks old, weight 200-300g, supplied by a biotechnology company in Beijing) to evaluate the metabolic characteristics of compound GP262 through intravenous injection (IV) and intraperitoneal injection (IP). The IV group received a dose of 5 mg/kg (2.5 mg/mL concentration, 2 mL/kg administration), while the IP group received a dose of 15 mg/kg (1.5 mg/mL concentration, 10 mL/kg administration). Each group consisted of 3 fasted animals. The IV group solvent was a mixed system containing 10% DMSO, 60% PEG400, and 30% water. Collect whole blood samples at 5 minutes, 15 minutes, 0.5 hours, 1, 2, 4, 7, and 24 hours after administration. Immediately after collection with EDTA-K2 anticoagulant tube, centrifuge at 4 °C and 4000 × g for 5 minutes. Separate the plasma and transfer it to an anticoagulant free EP tube. Store it at -75 ± 15 °C for testing at ultra-low temperature. Throughout the experiment, a dual drug group parallel control was set up, and standardized operating procedures were used to ensure the timeliness and storage stability of plasma separation, providing standardized biological samples for subsequent LC-MS/MS analysis.

Evaluation of the therapeutic effects

10 mice were randomly divided into 2 groups (n=5 in each group) to establish a subcutaneous xenograft tumor cell model. The drug was administered when the tumor tissue grew to about 100 cubic centimeters (i.p.). Record weight and tumor size every two days. After 14 days of administration, the mice were euthanized and the tumor tissue was fixed in 4% paraformaldehyde (histological examination) or frozen in liquid nitrogen (biochemical analysis).

Histological examinations

For IHC staining, tissue sections after antigen retrieval were blocked with 5% nonfat dry milk at 37°C for 1 h and incubated overnight with corresponding primary antibodies at 4°C. After 3 washes

with PBS, slices were incubated with HRP-conjugated secondary antibodies at 37℃ for 1 h. After another 3 washes with PBS, slices were stained using a 3,3'-diaminobenzidine (DAB) substrate kit (all reagents for histological examinations were purchased from Servicebio, Shanghai, China) and counterstained with hematoxylin. For H&E staining, tissue sections were processed with corresponding staining kits according to the manufacturer's instructions. The histological features were observed and captured using a fluorescent microscope (Axio Observer Z1, Zeiss) or a slide scanner (Pannoramic MIDI, 3D Histech, Budapest, Hungary). The captured images were analyzed by ImageJ software.

Statistics and data visualization

Statistical analysis was performed using GraphPad Prism software (Ver. 8.3.0), SPSS Statistics software (Ver. 21.0), and R (Ver. 4.2.0). Experimental data were expressed as mean ± SEM, and statistical significances between corresponding groups were assessed by one-way ANOVA, and multiple comparisons were performed using Tukey's tests. The visualization of data and results was powered by GraphPad Prism and BioRender.com.

Experimental steps and parameters for DIA relative quantitative proteomics
1.1 Peptide Sample Preparation

1. Based on protein concentration, take 30–50 μg of the sample protein and supplement with an appropriate volume of lysis buffer24.
2. Add TEAB to achieve a final concentration of 100 mM in the solution.
3. Add TCEP to achieve a final concentration of 10 mM, then incubate at 37°C with shaking for 60 min3.
4. Add IAM (iodoacetamide) to a final concentration of 40 mM, and react at room temperature under light-protected conditions for 40 min3.
5. Add 800 μL of acetone, and precipitate proteins at –20°C for 2 h.
6. Centrifuge at 12,000 ×g for 15 min, discard supernatant, and wash the precipitate with 800 μL of 90% acetone solution4.
7. Centrifuge at 12,000 ×g for 15 min, discard supernatant, and air-dry in a fume hood for 5 min.
8. Add trypsin at an enzyme-to-protein mass ratio (m/m) of 1:40, and digest at 37°C with shaking overnight3.

1.2 Peptide Desalting

Sola Cartridge Protocol

1. **Activation**: Load 200 μL acetonitrile once.
2. **Equilibration**: Load 200 μL of 0.1% TFA once.
3. **Sample Loading**: Load the sample (pH < 3) twice.
4. **Wash**: Load 800 μL of 0.1% TFA once.
5. **Wash**: Load 200 μL water once.
6. **Elution**: Load 100 μL of 70% acetonitrile once2.

1.3 Peptide Quantification

Quantify peptides using ultraviolet spectrophotometry via a NanoDrop One (Thermo Scientific)4.

1.4 Mass Spectrometry Analysis

1.4.1 Liquid Chromatography Method

Dissolve peptides in MS loading buffer (0.1% formic acid) based on quantification results2.

- **Software**: Compass HyStar (Bruker, Germany)
- **Column**: Homemade column (15 cm × 100 μm, 1.7 μm)
- **LC System**: Vanquish Neo (Thermo, USA)
- **MS System**: Astral (Thermo, USA)
- **Run Time**: 8 min
- **Mobile Phase**:
  - **A**: 98% water, 2% acetonitrile, 0.1% formic acid
  - **B**: 80% acetonitrile, 20% water, 0.1% formic acid
  - **Gradient Program**:

**Time (min)**	**B (%)**
0	8.0
1	17.0
5.5	55.0
7	99.0
8	99.0

1.4.2 Mass Spectrometry Method

Samples separated by nanoLC were analyzed using an Astral mass spectrometer in DIA mode4.

- **Ionization Mode**: Positive ion
- **Ion Source Voltage**: 2.2 kV
- **Scan Ranges**:
  - MS1: 380–980 *m/z*

- MS2: 150–2000 *m/z*

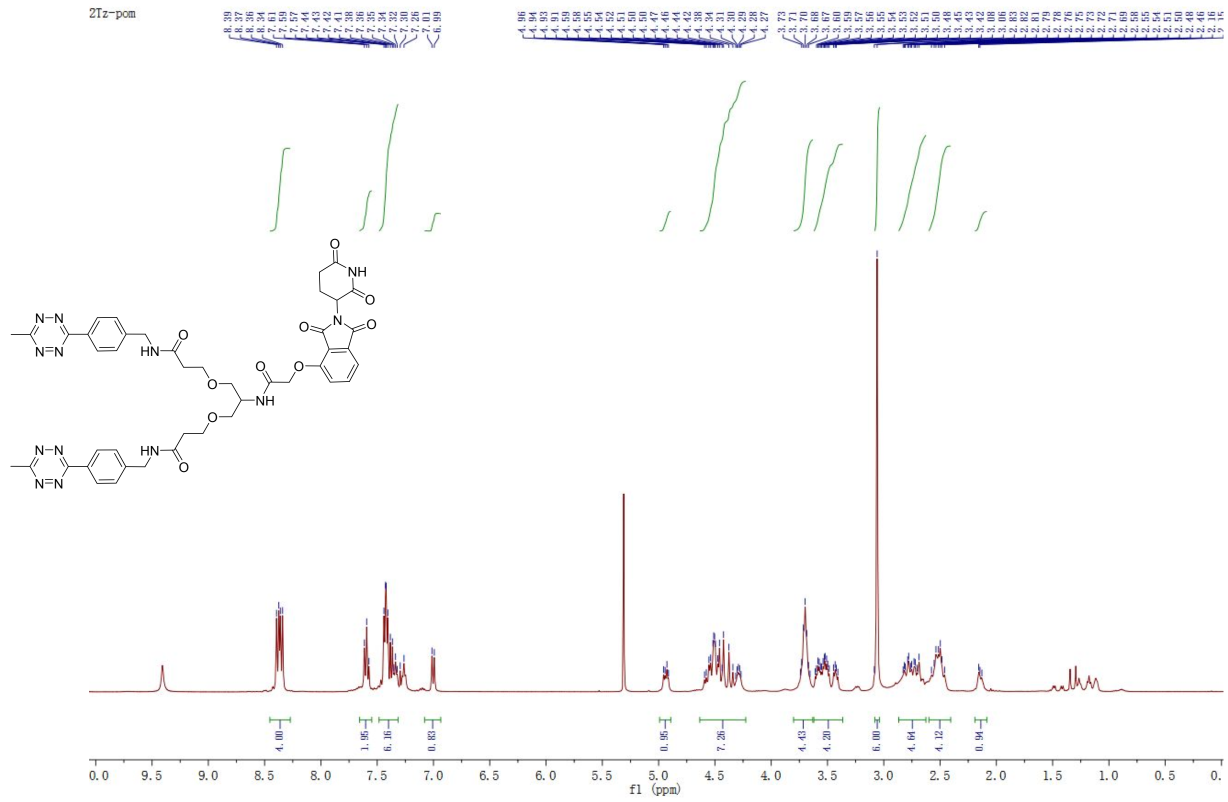

^{1}H NMR spectrum of POM-dTz

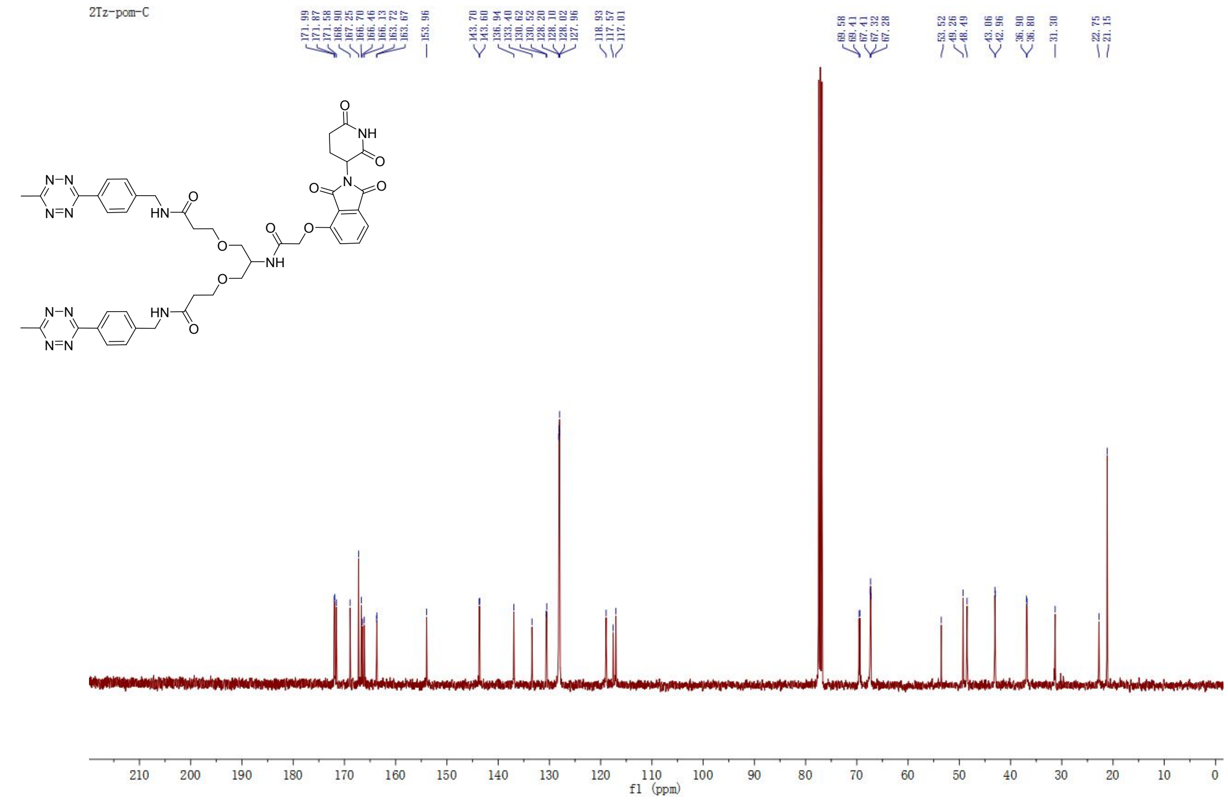

^{13}C NMR spectrum of POM-dTz

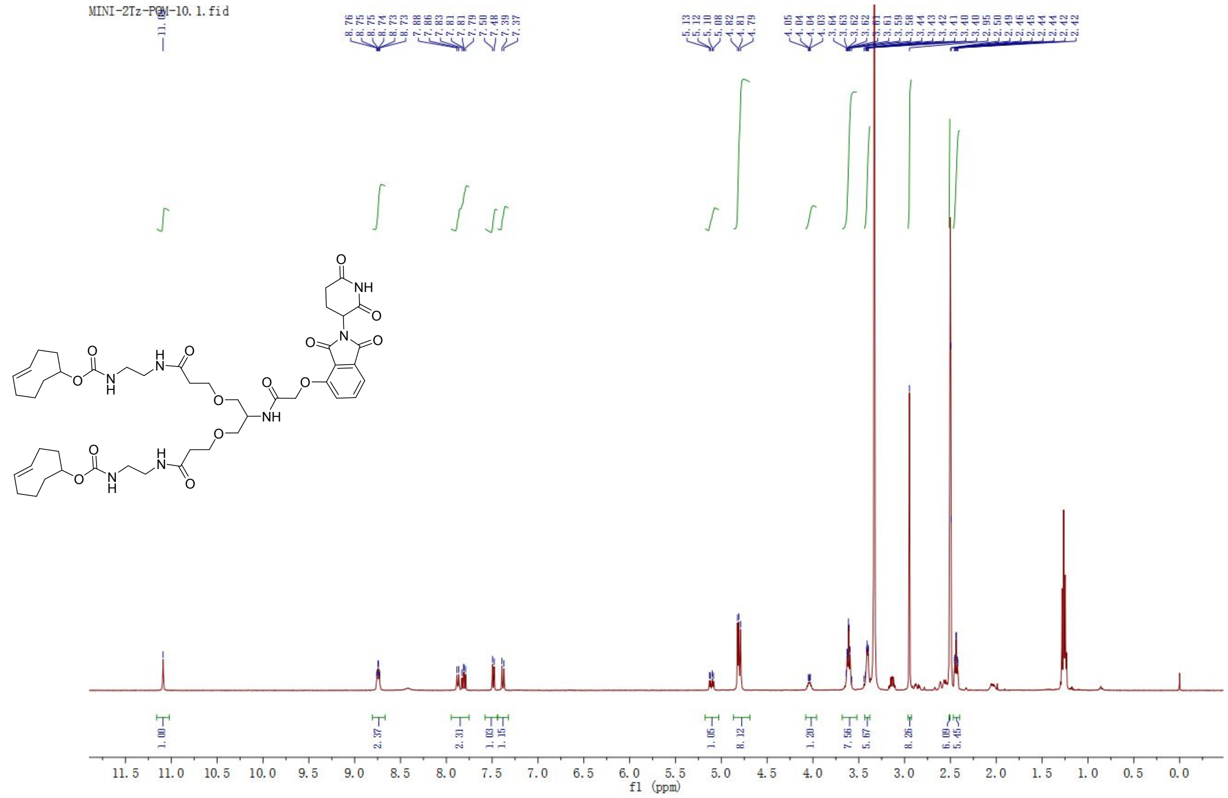

^{1}H NMR spectrum of POM-dTCO

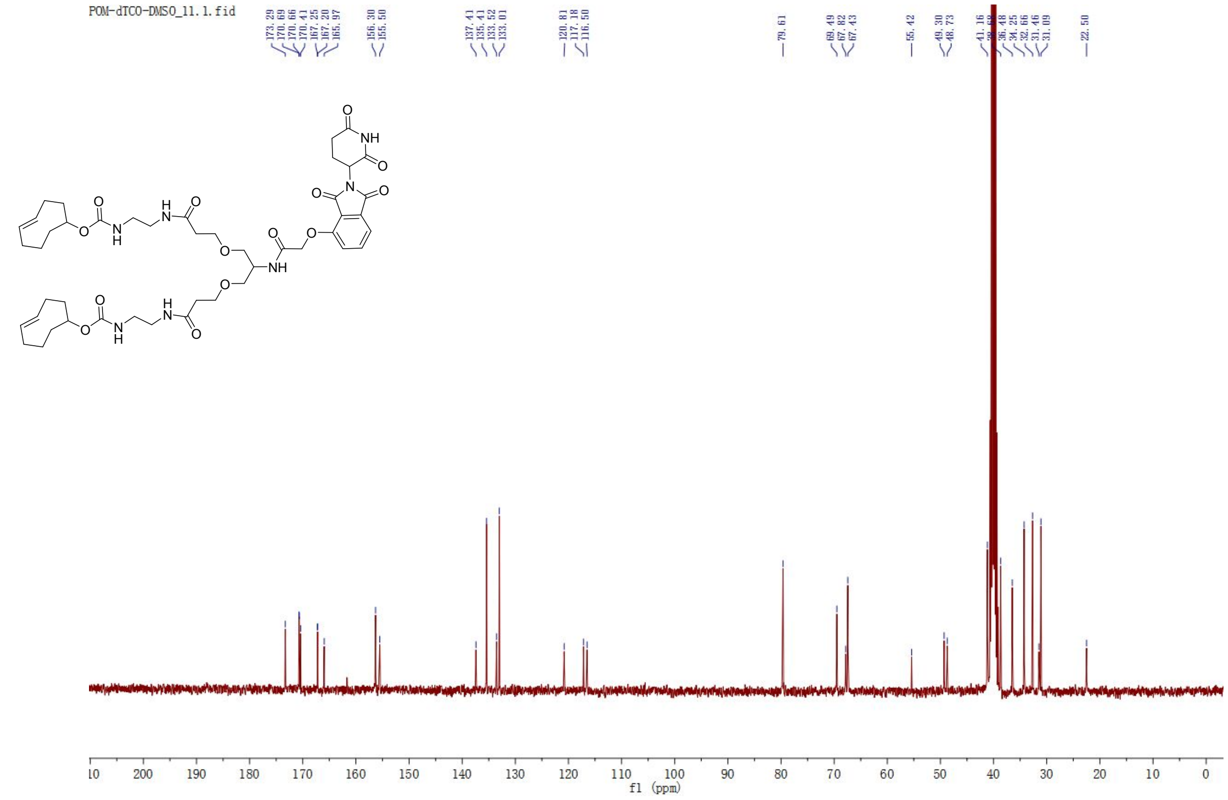

^{13}C NMR spectrum of POM-dTCO

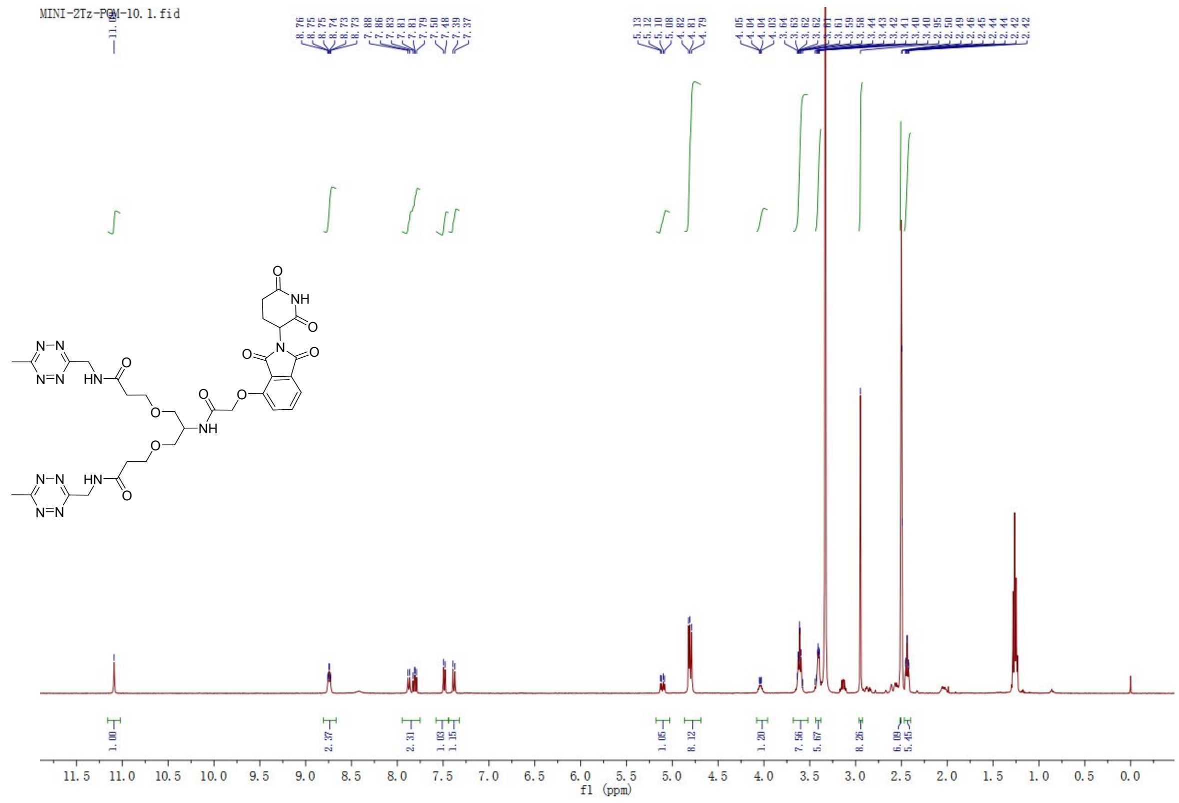

^{1}H NMR spectrum of POM-dmTz

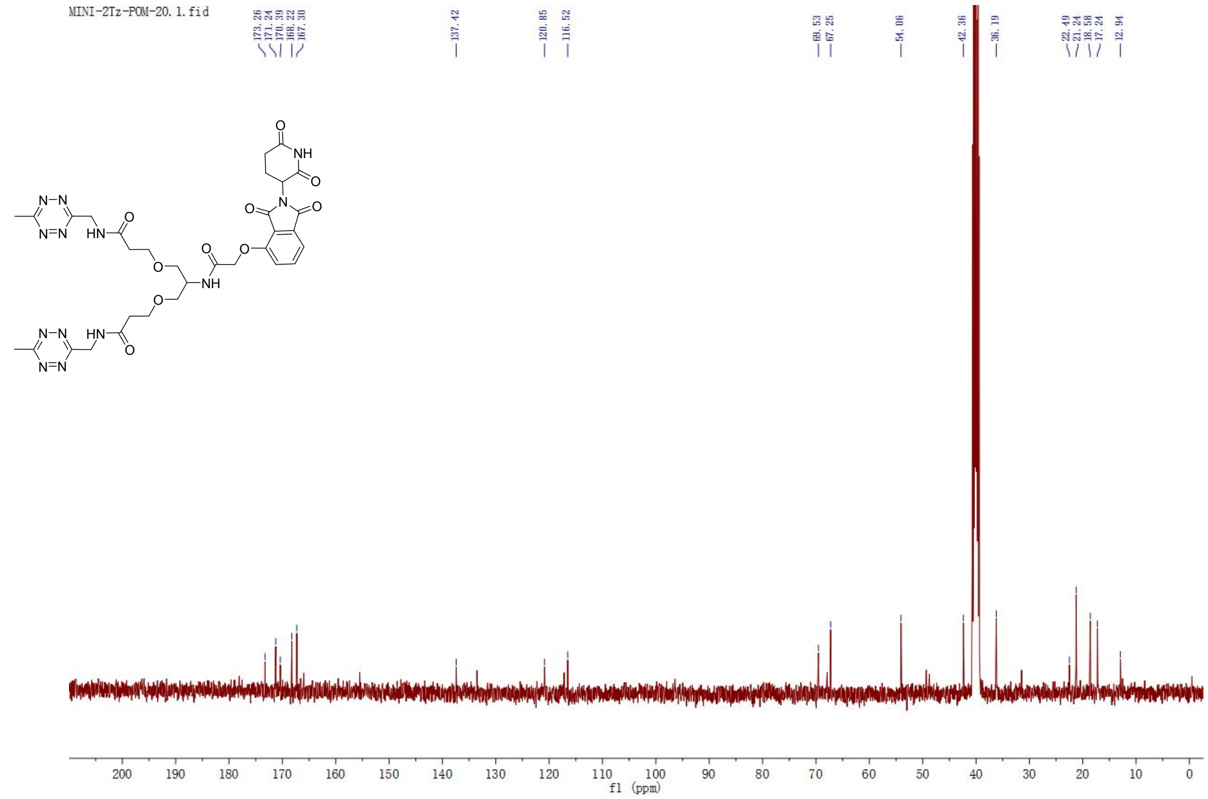

^{13}C NMR spectrum of POM-dmTz

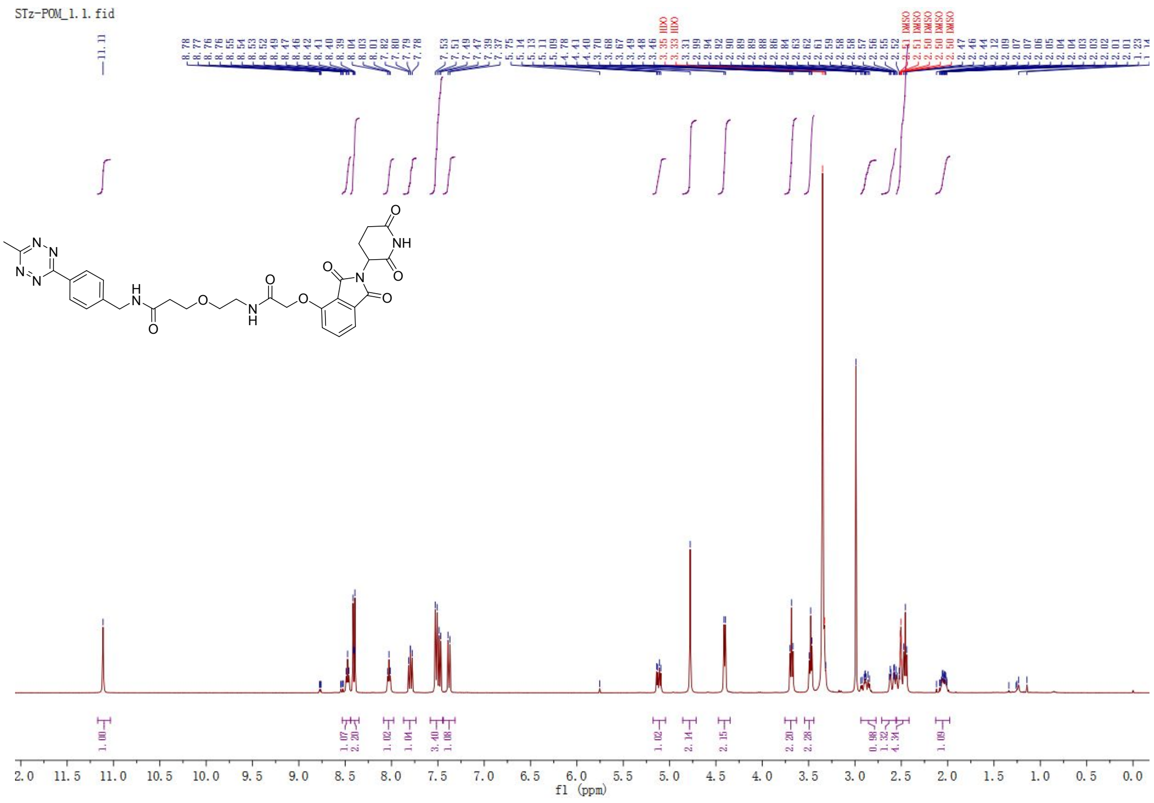

^{1}H NMR spectrum of POM-STz

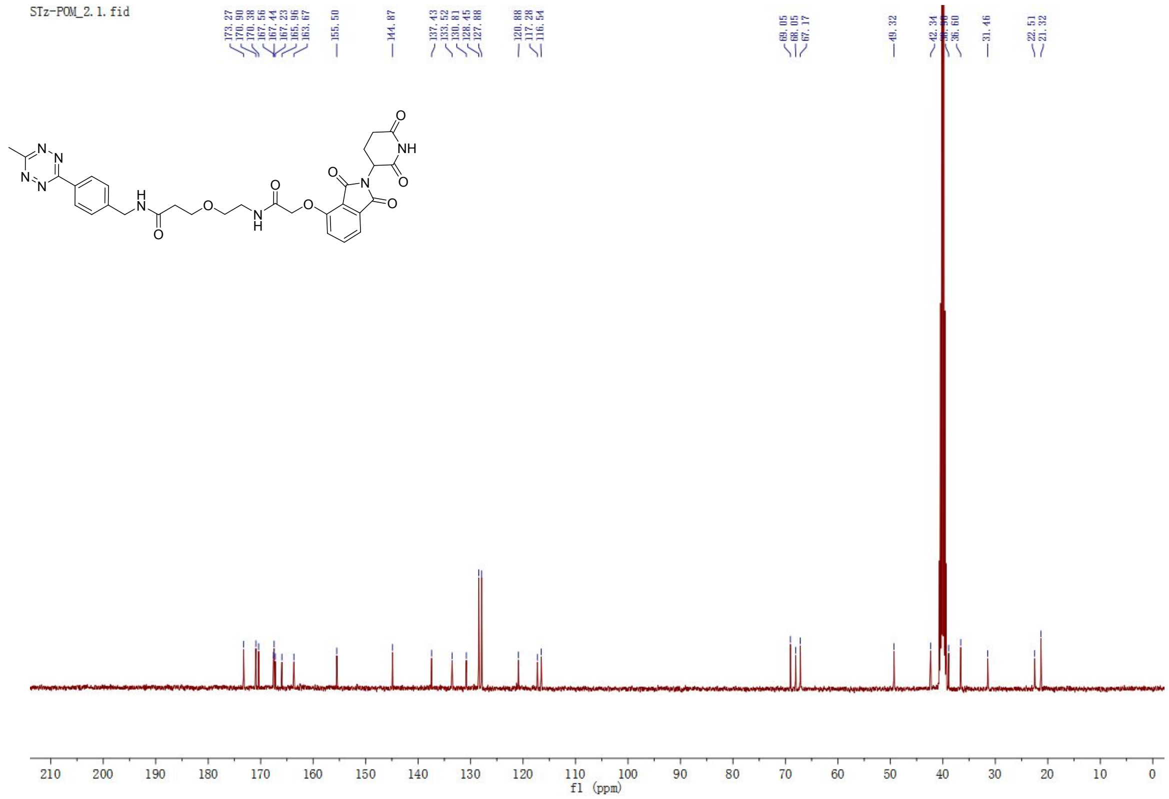

^{13}C NMR spectrum of POM-STz

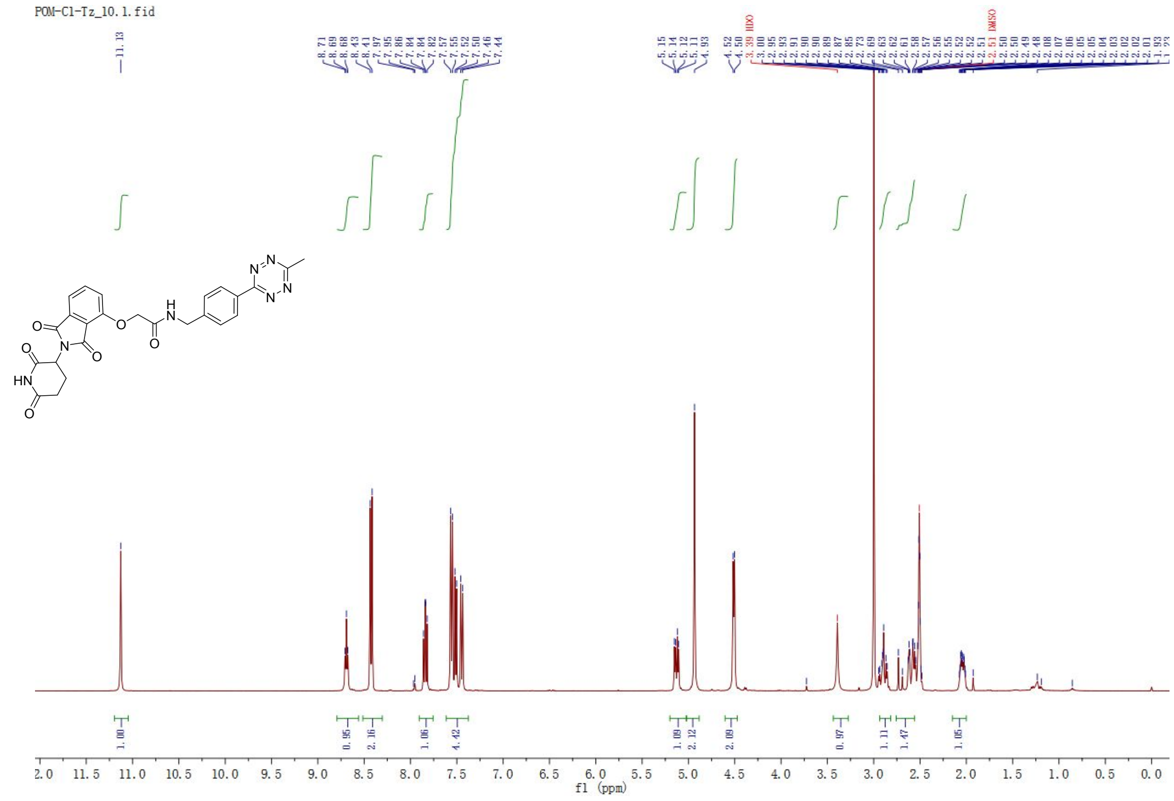

^{1}H NMR spectrum of POM-C1-Tz

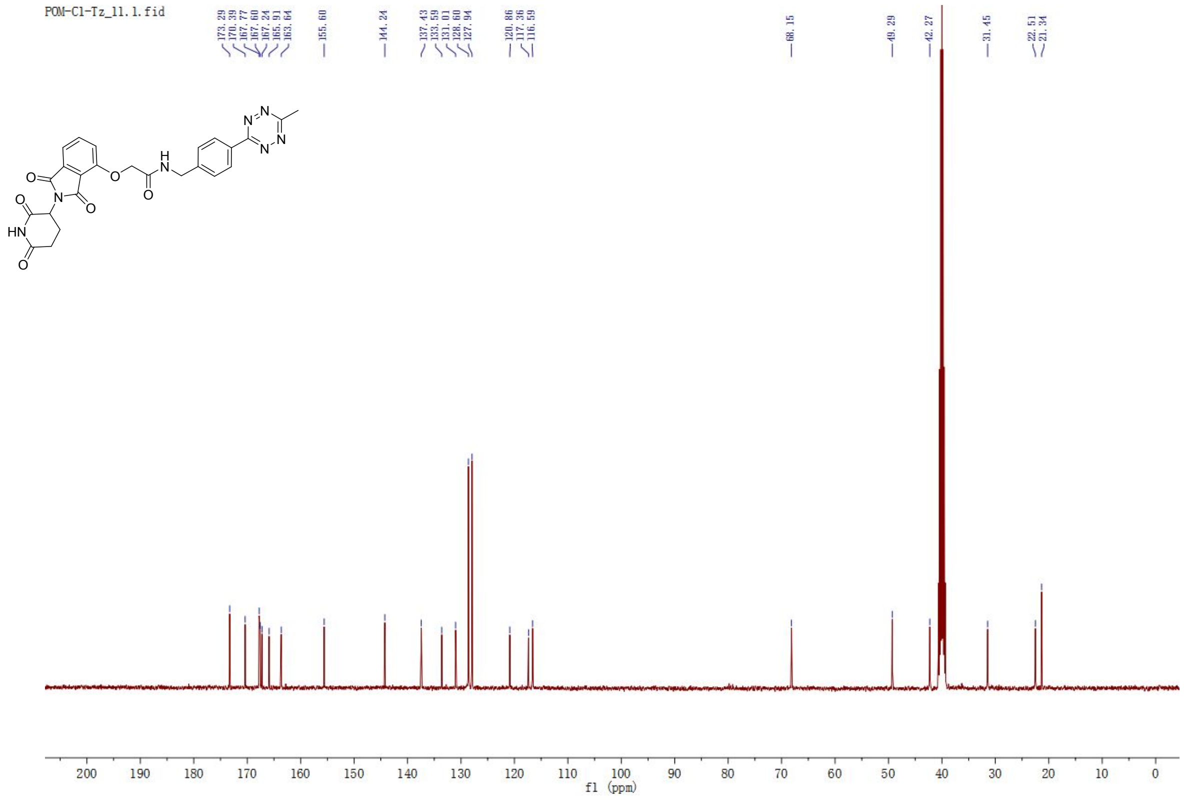

^{13}C NMR spectrum of POM-C1-Tz

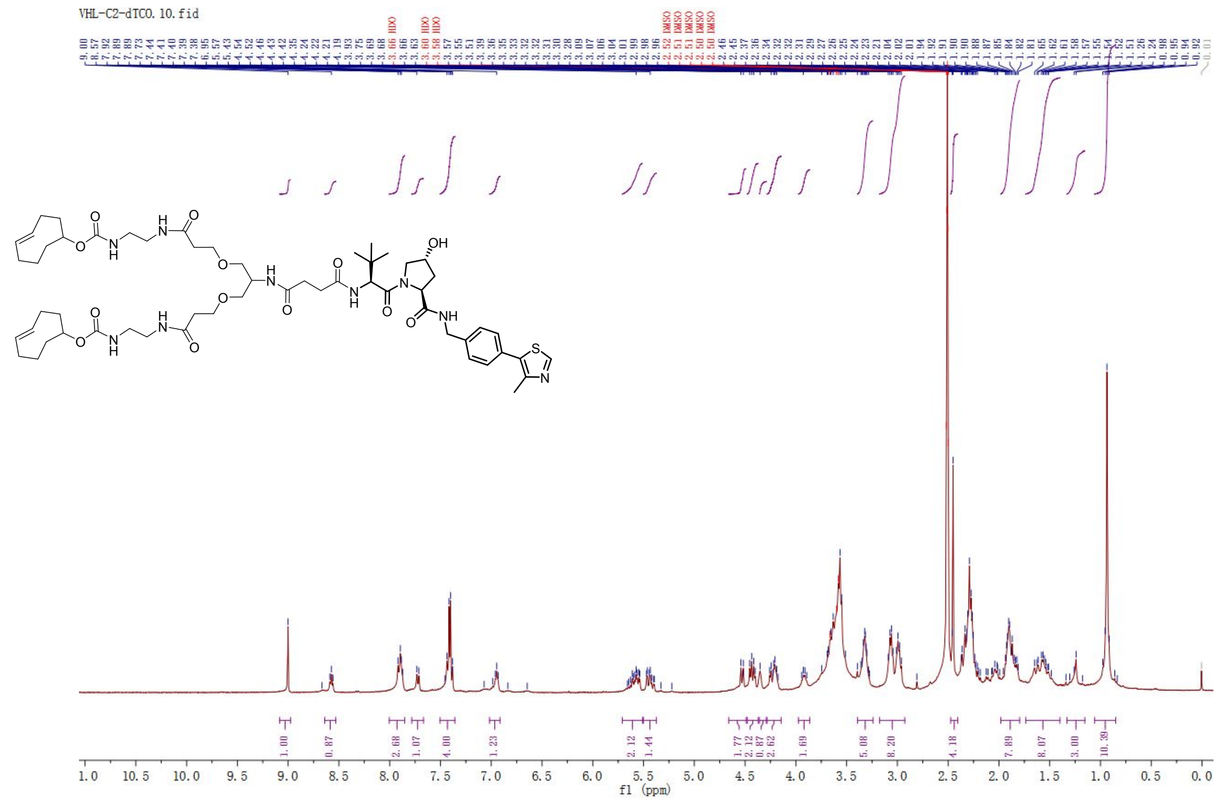

^{1}H NMR spectrum of VH032-dTCO

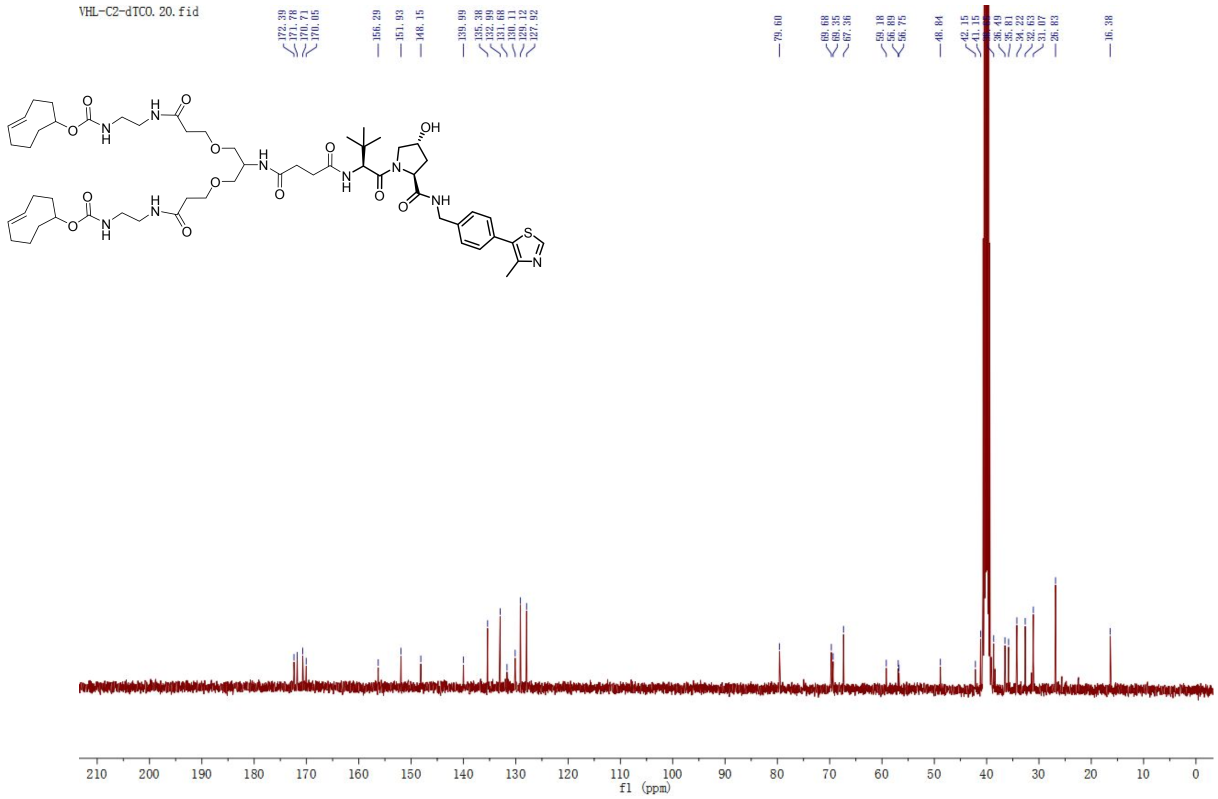

^{13}C NMR spectrum of VH032-dTCO

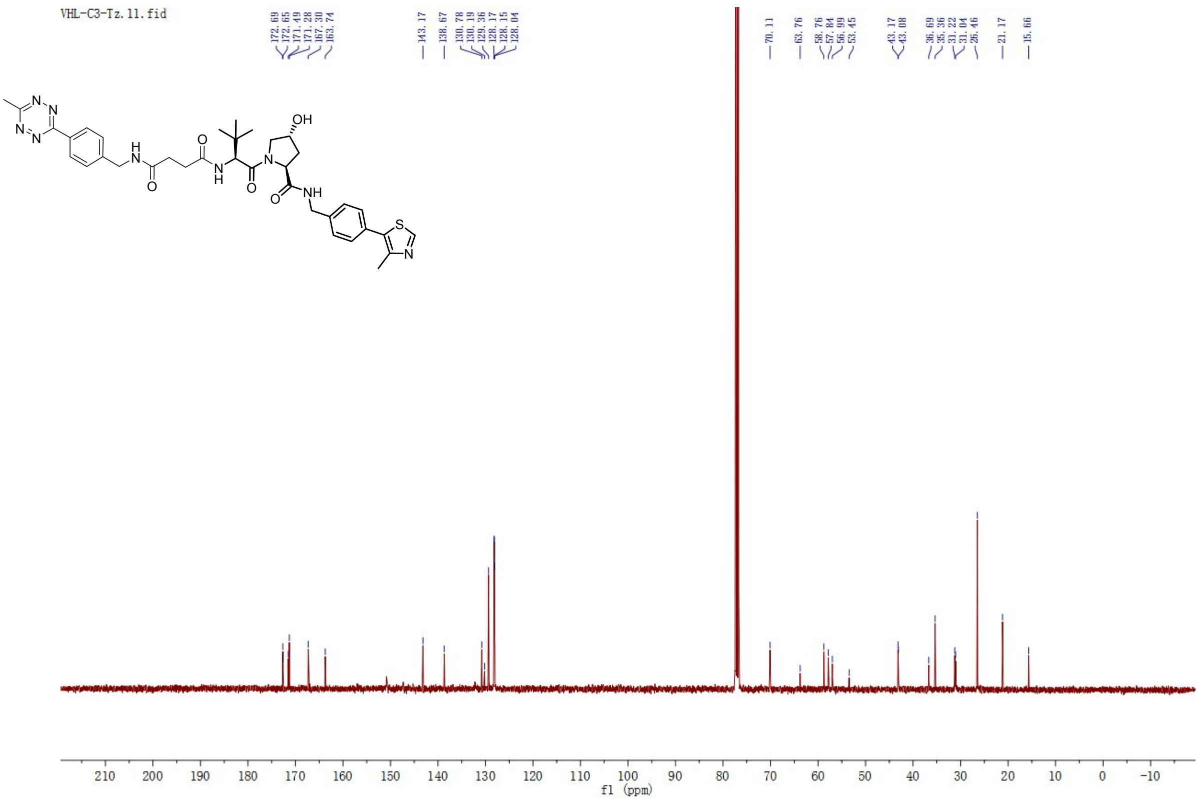

^{1}H NMR spectrum of VH032-C2-Tz

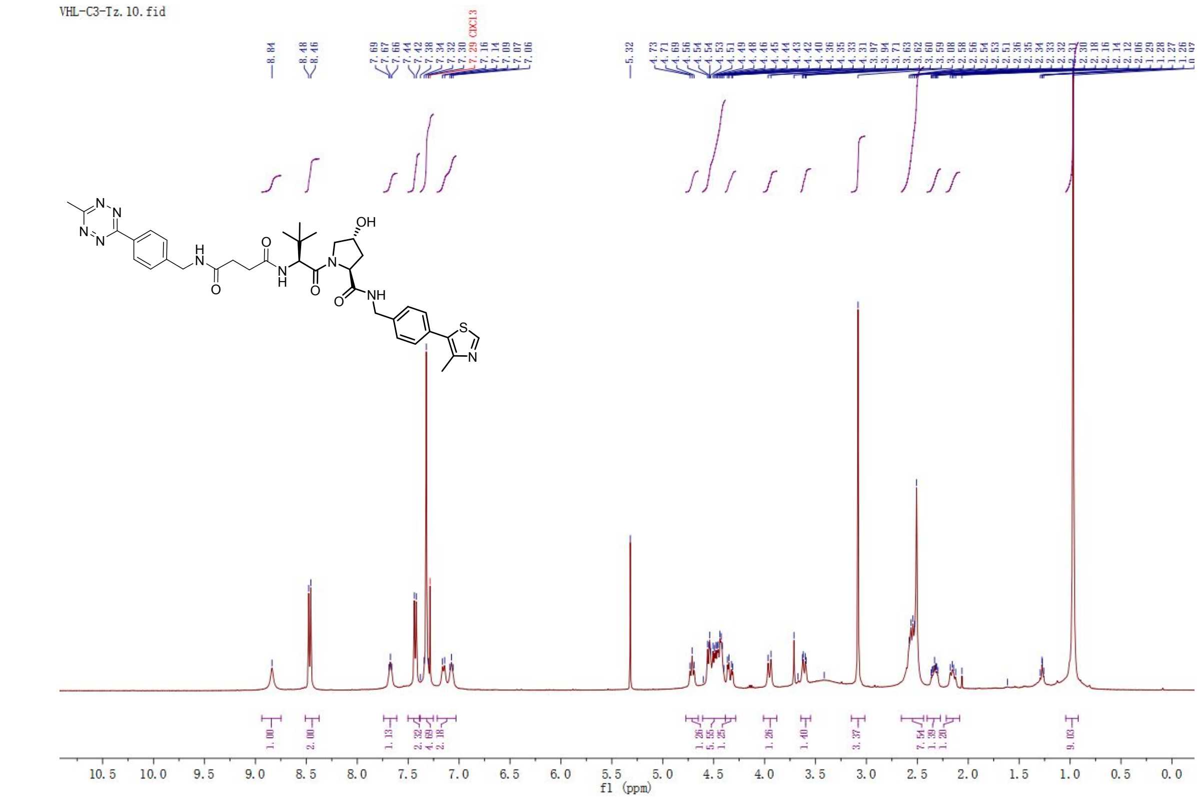

^{13}C NMR spectrum of VH032-C2-Tz

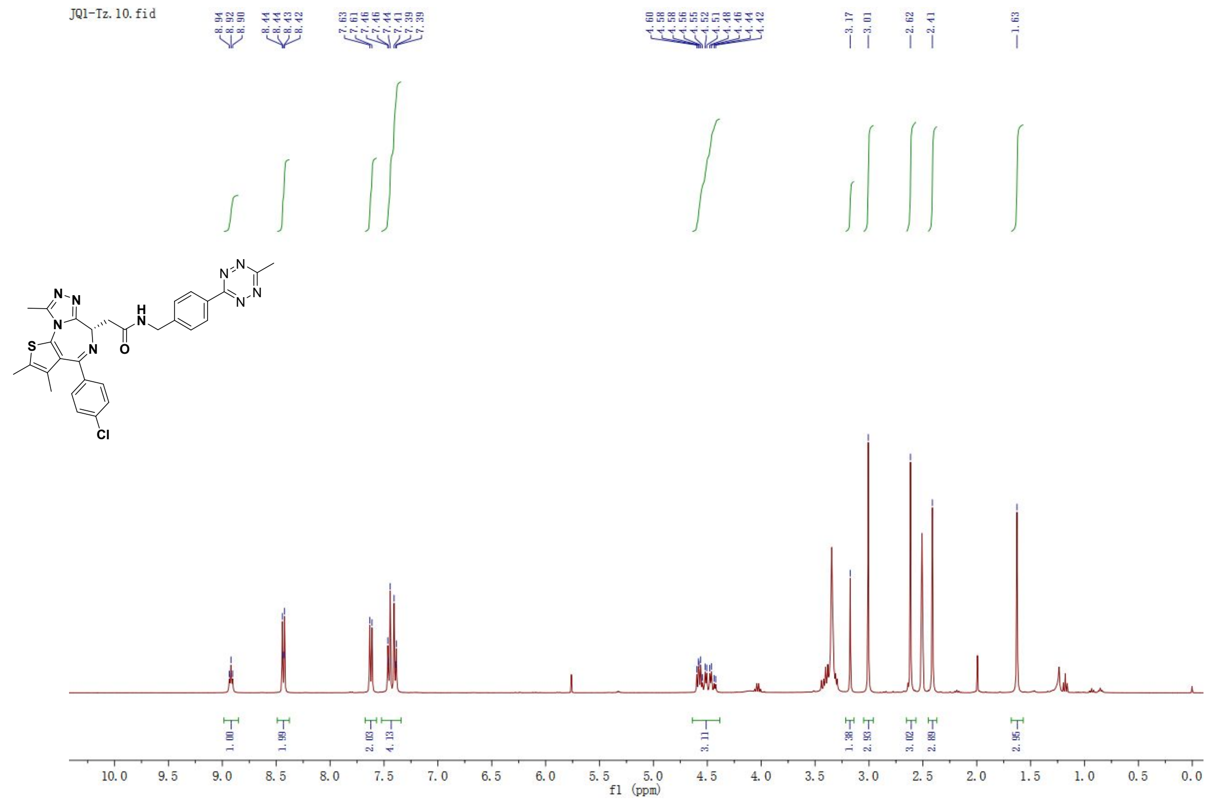

^{1}H NMR spectrum of JQ1-Tz

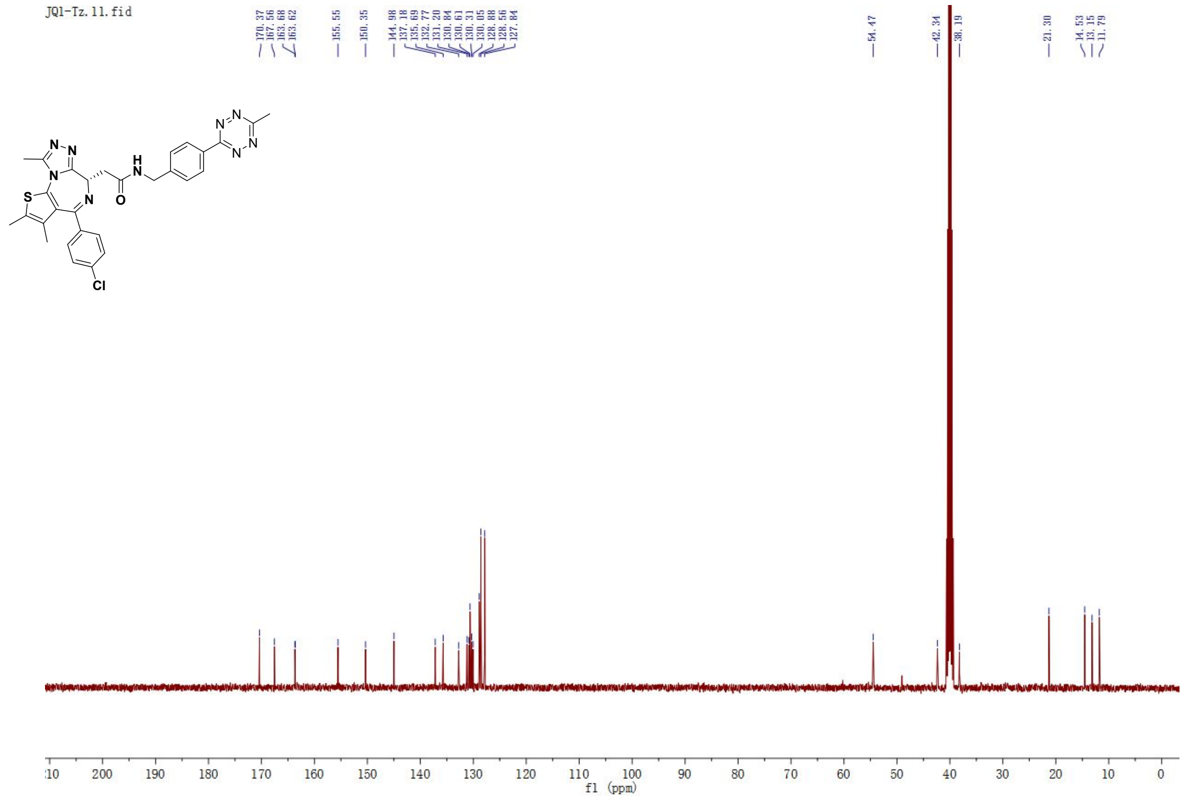

^{13}C NMR spectrum of JQ1-Tz

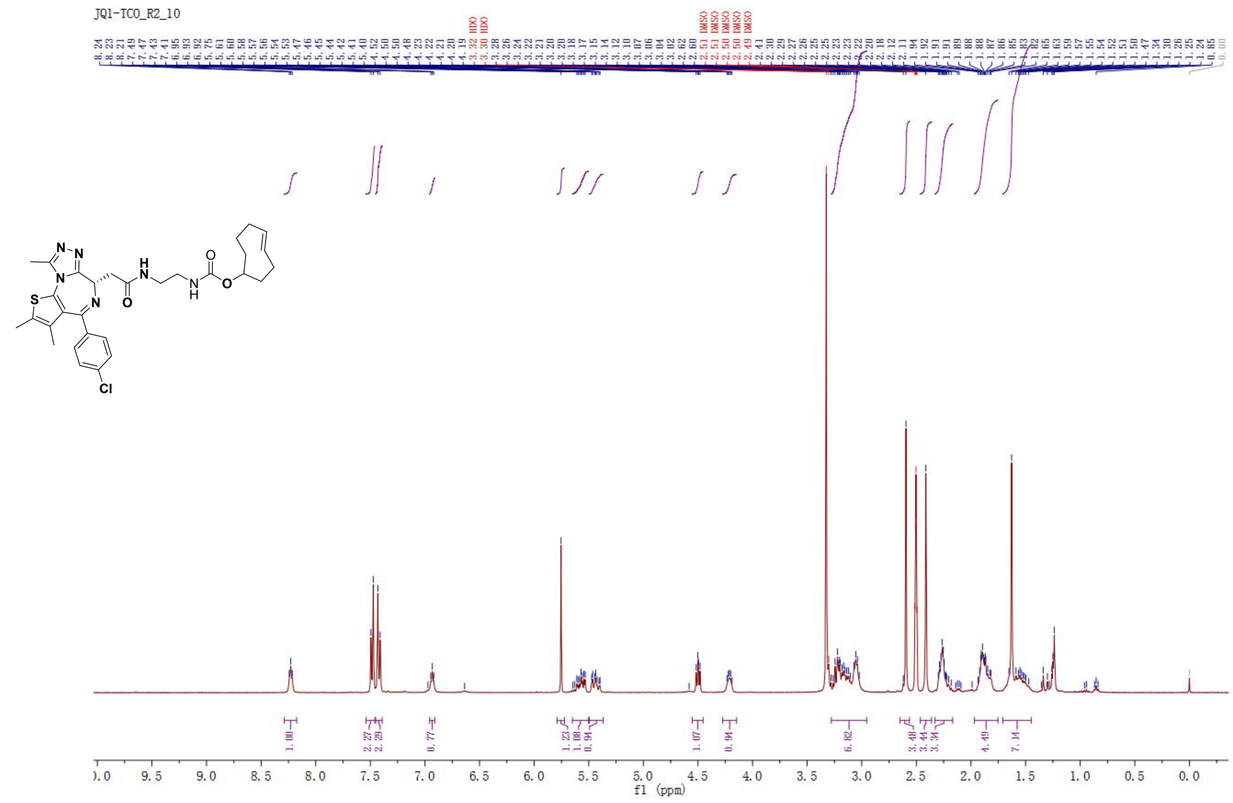

^{1}H NMR spectrum of JQ1-TCO

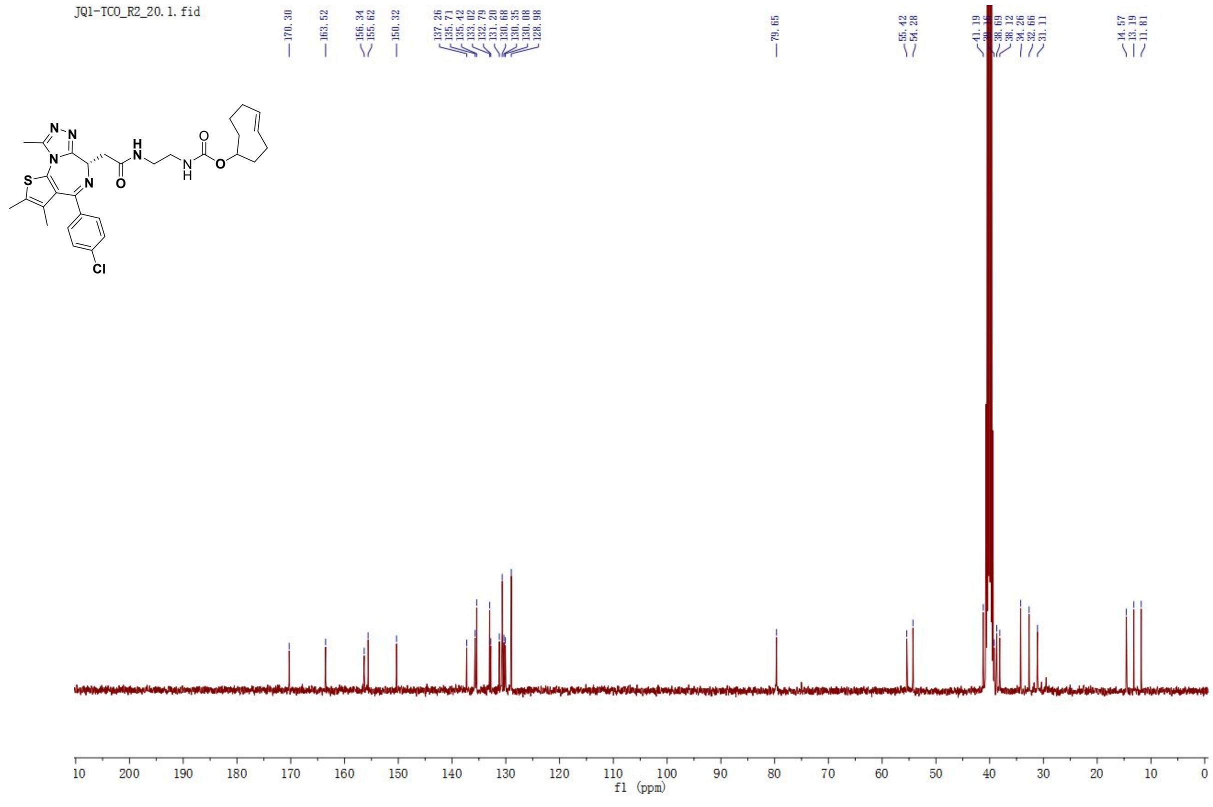

^{13}C NMR spectrum of JQ1-TCO

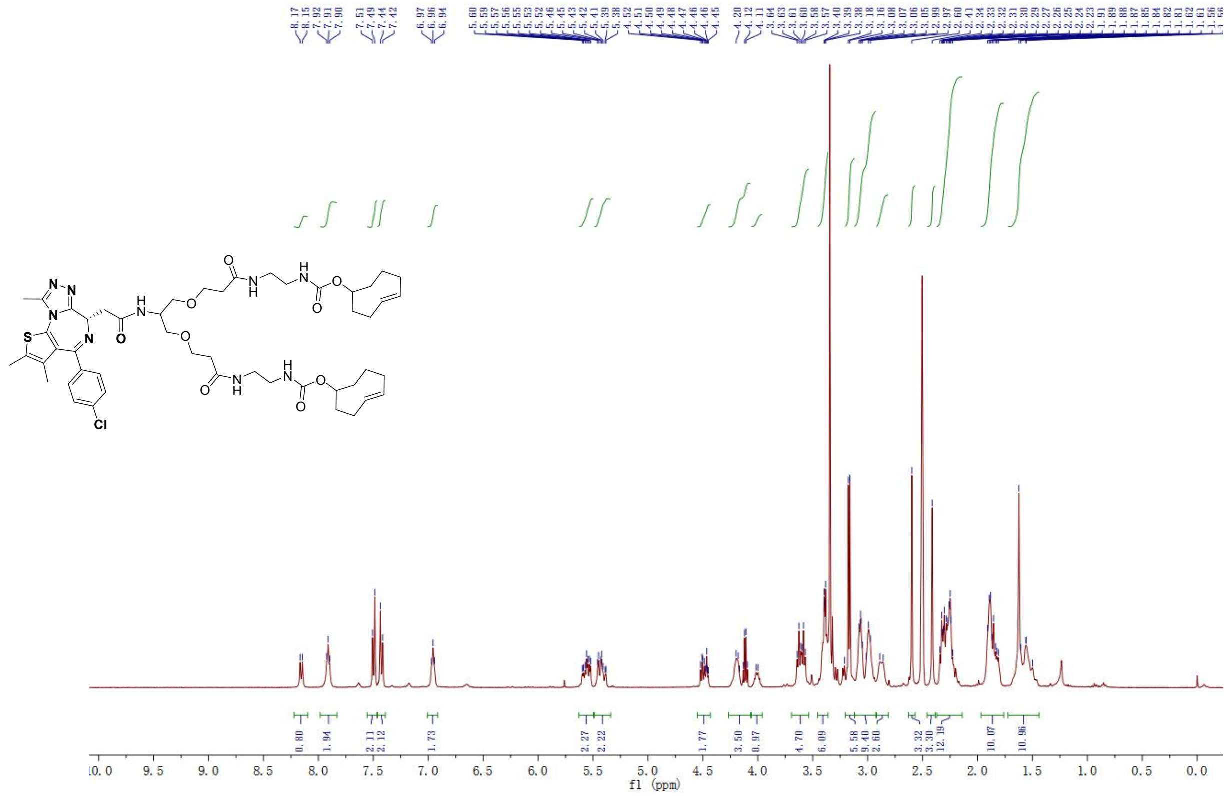

^{1}H NMR spectrum of JQ1-dTCO

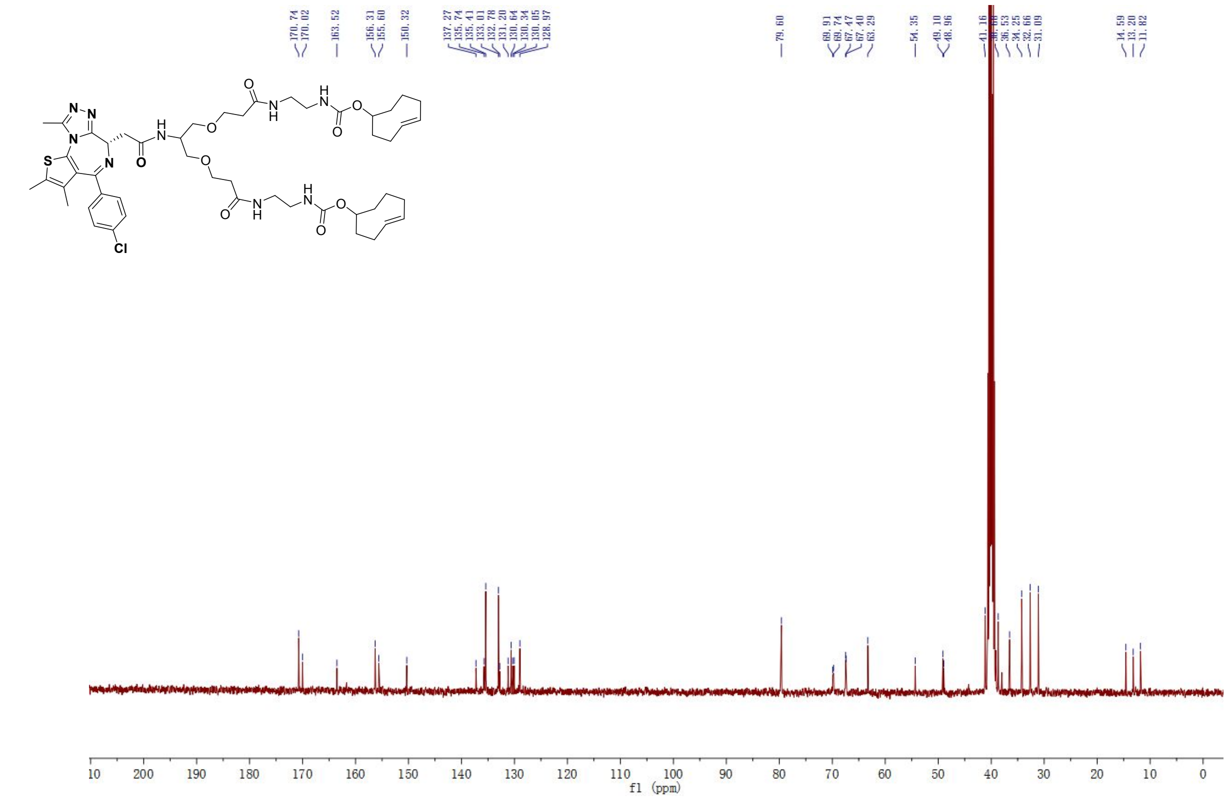

^{13}C NMR spectrum of JQ1-dTCO 的 ^{13}C NMR 谱图

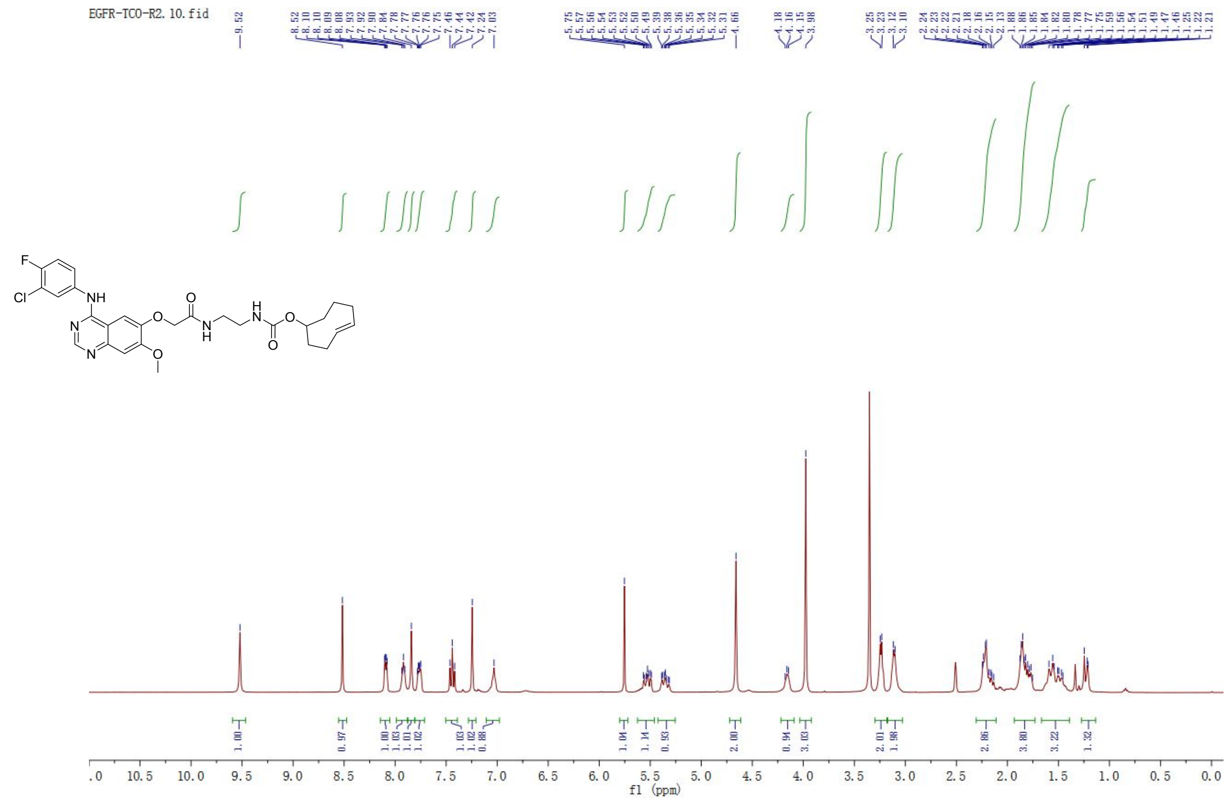

^{1}H NMR spectrum of EGFR-TCO

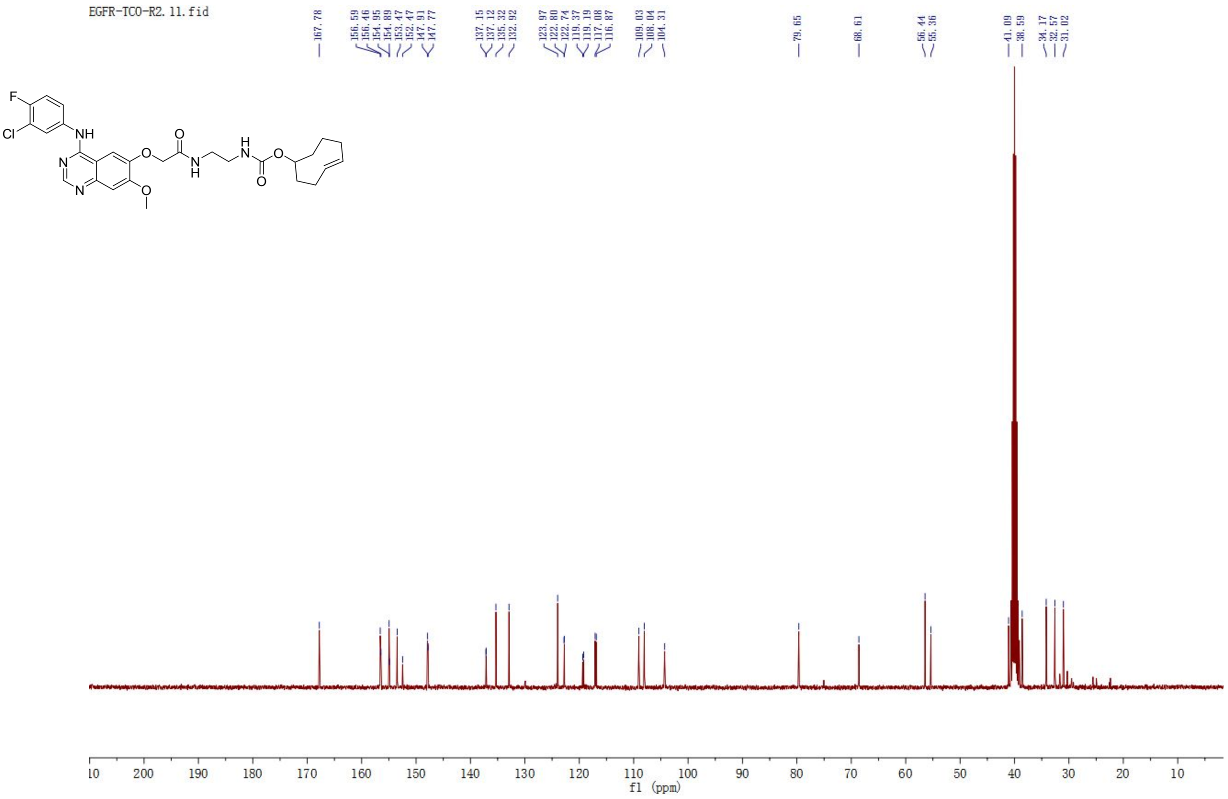

^{13}C NMR spectrum of EGFR-TCO

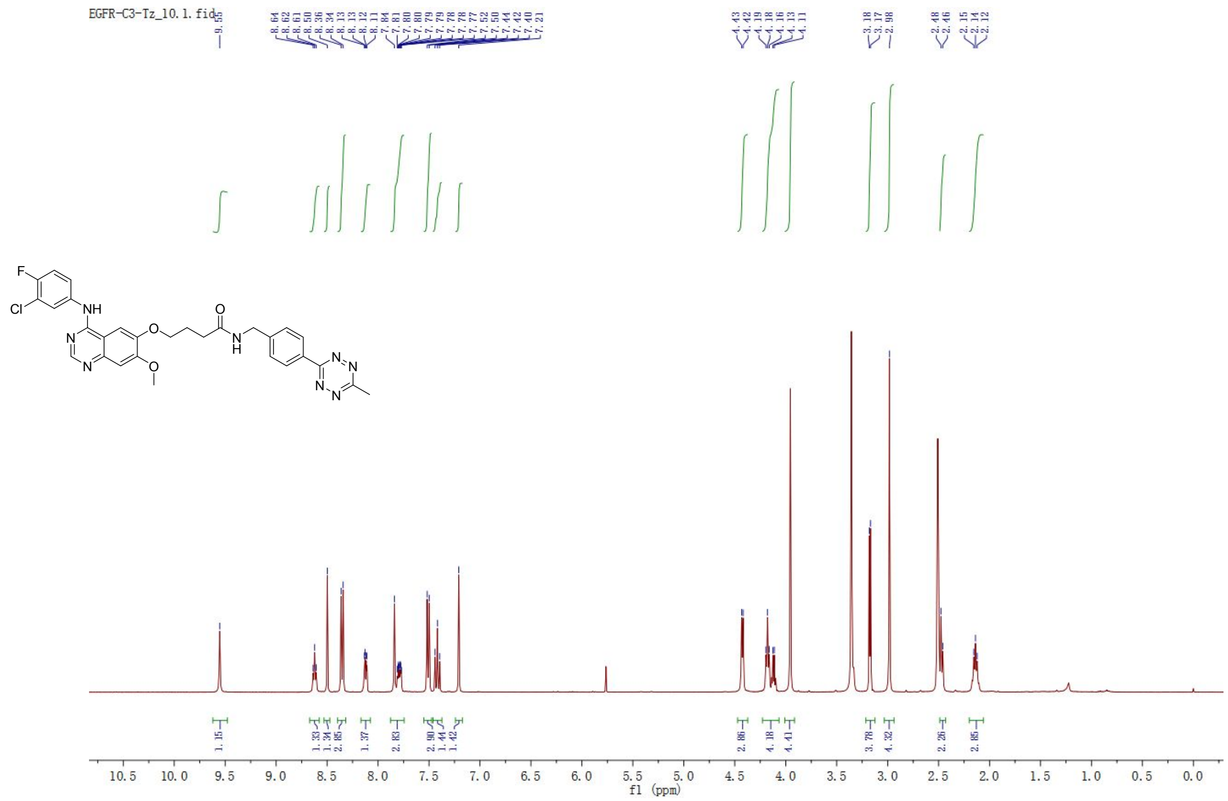

^{13}C NMR spectrum of EGFR-C3-Tz

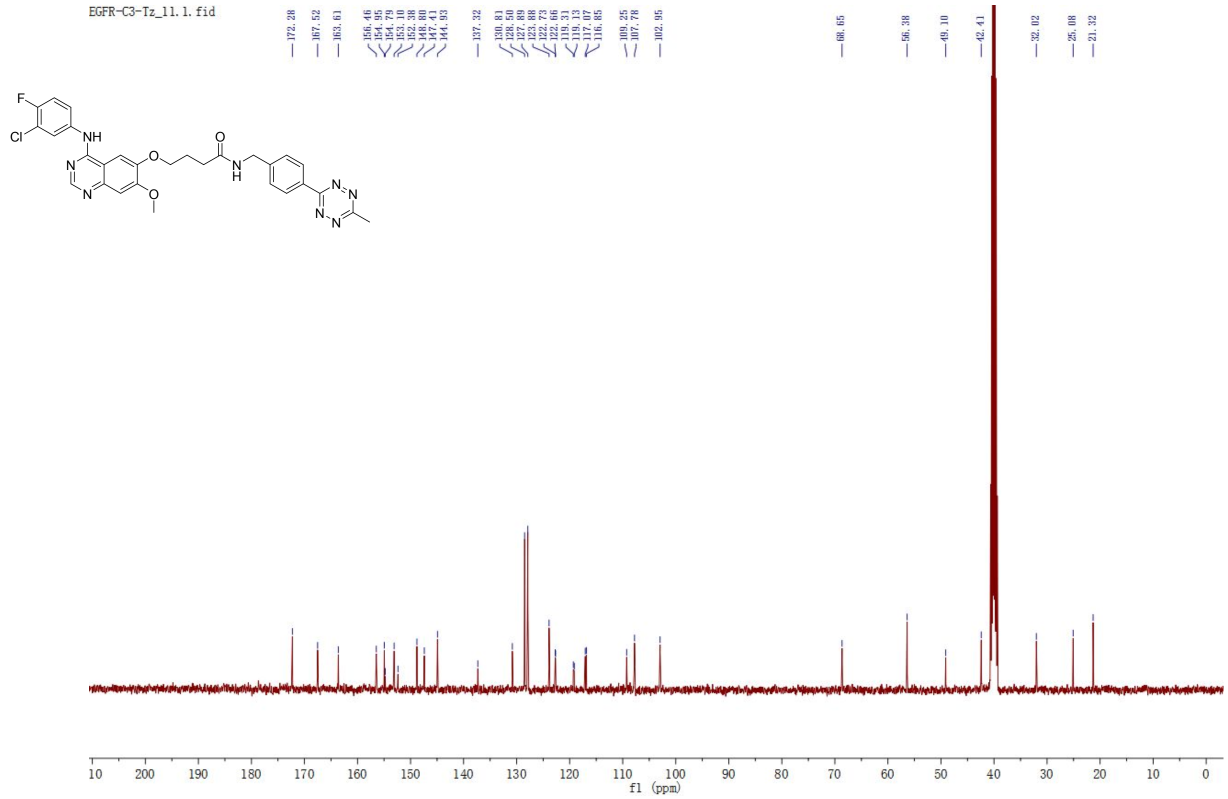

^{1}H NMR spectrum of EGFR-C3-Tz

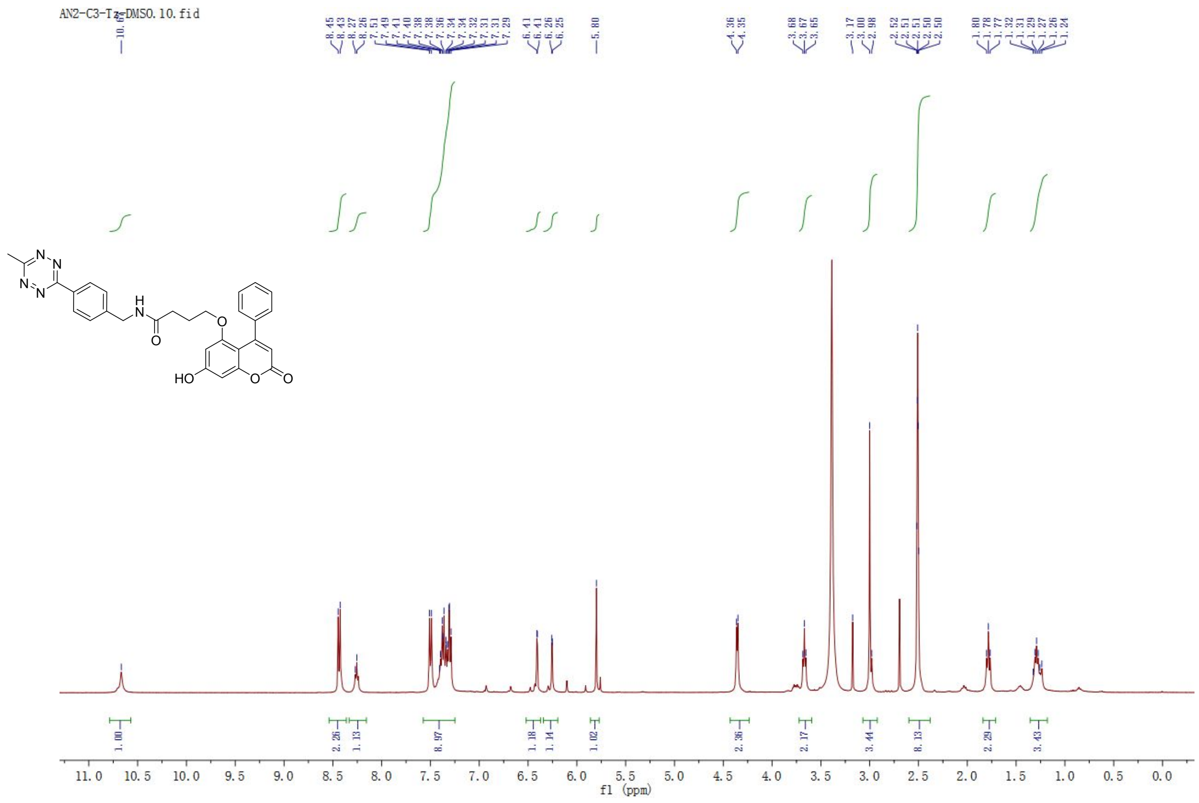

^{1}H NMR spectrum of AN2-C3-Tz

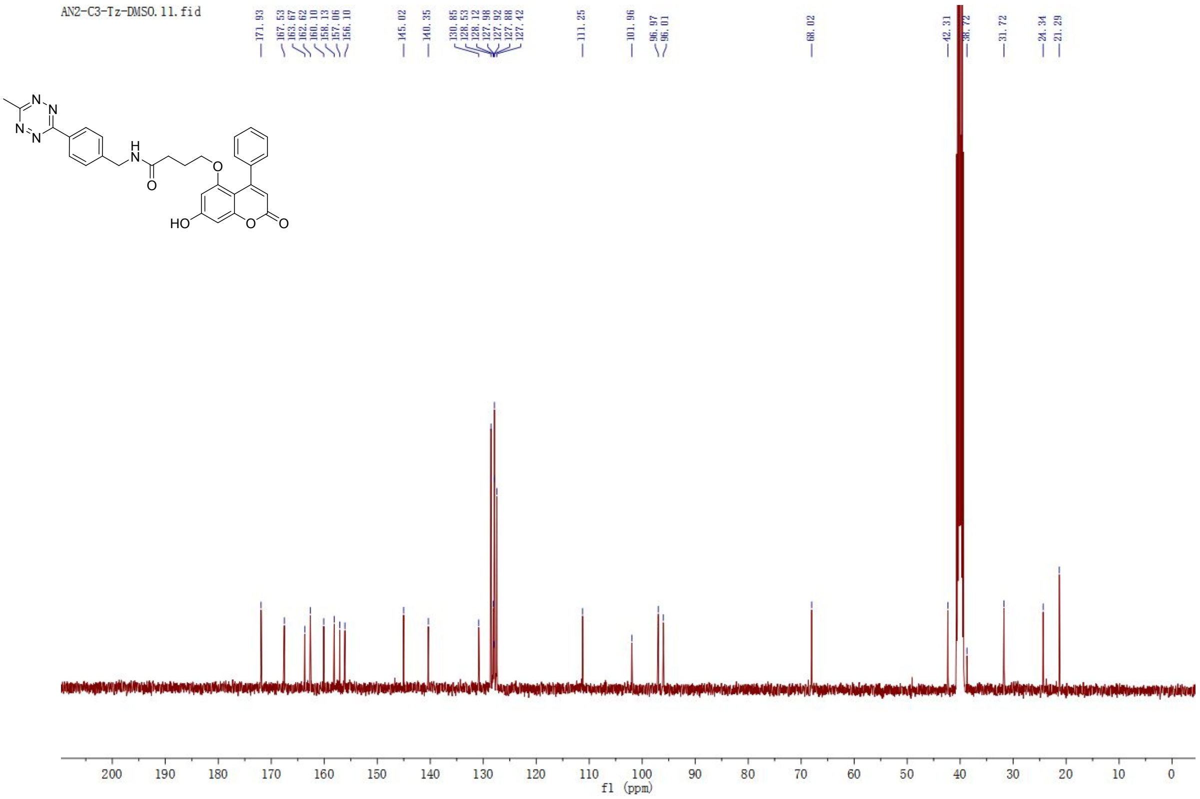

^{13}C NMR spectrum of AN2-C3-Tz

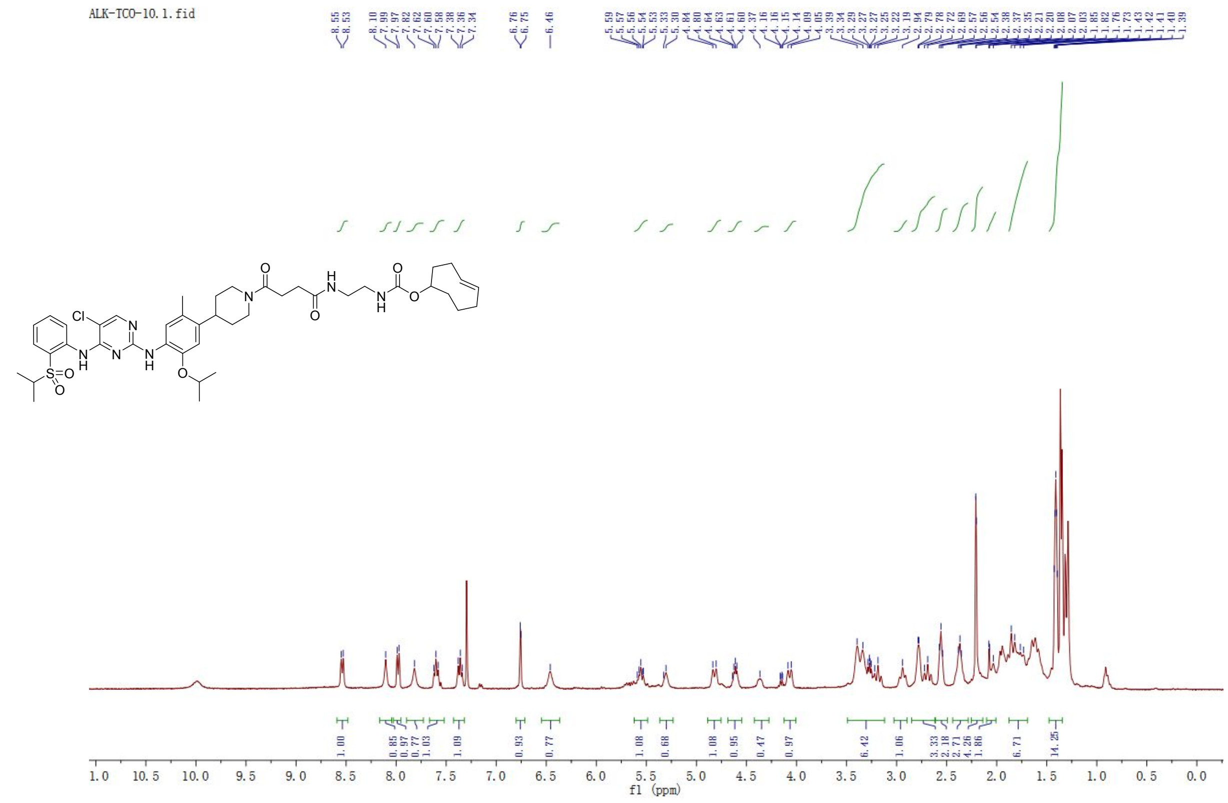

^{1}H NMR spectrum of ALK-TCO

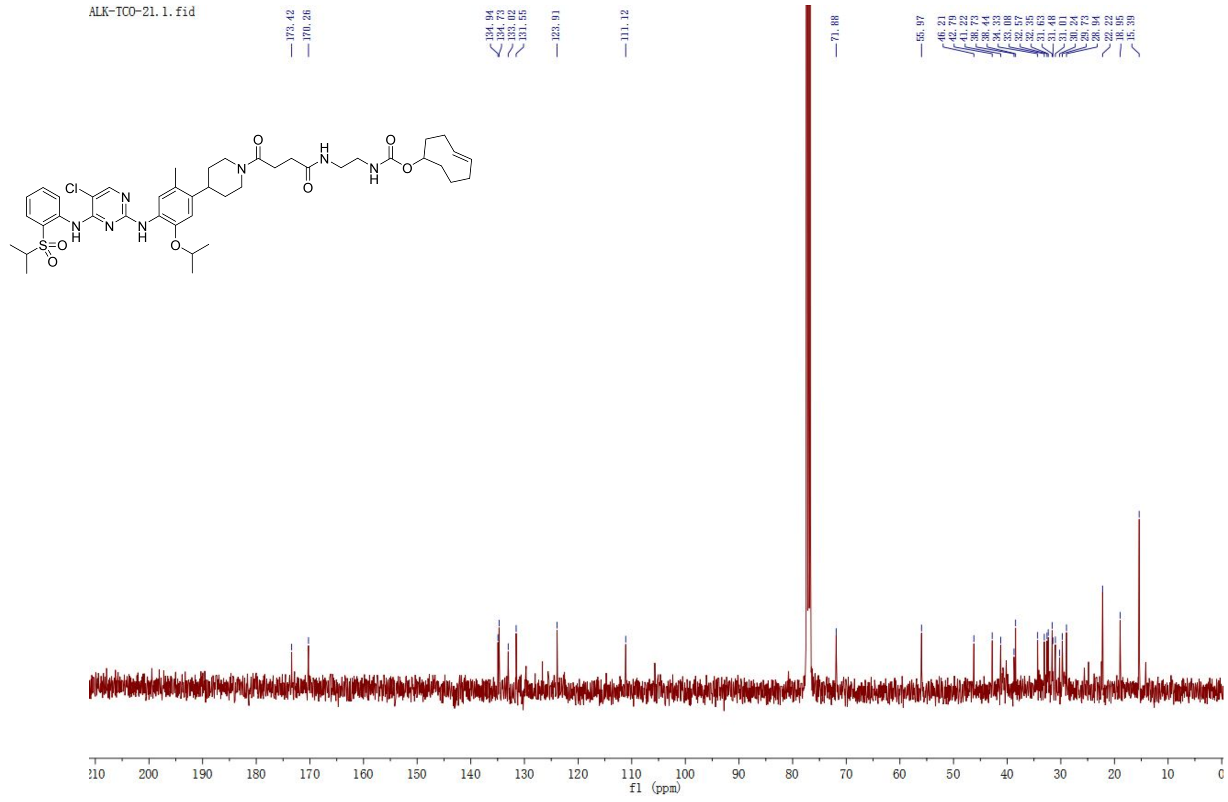

^{13}C NMR spectrum of ALK-TCO

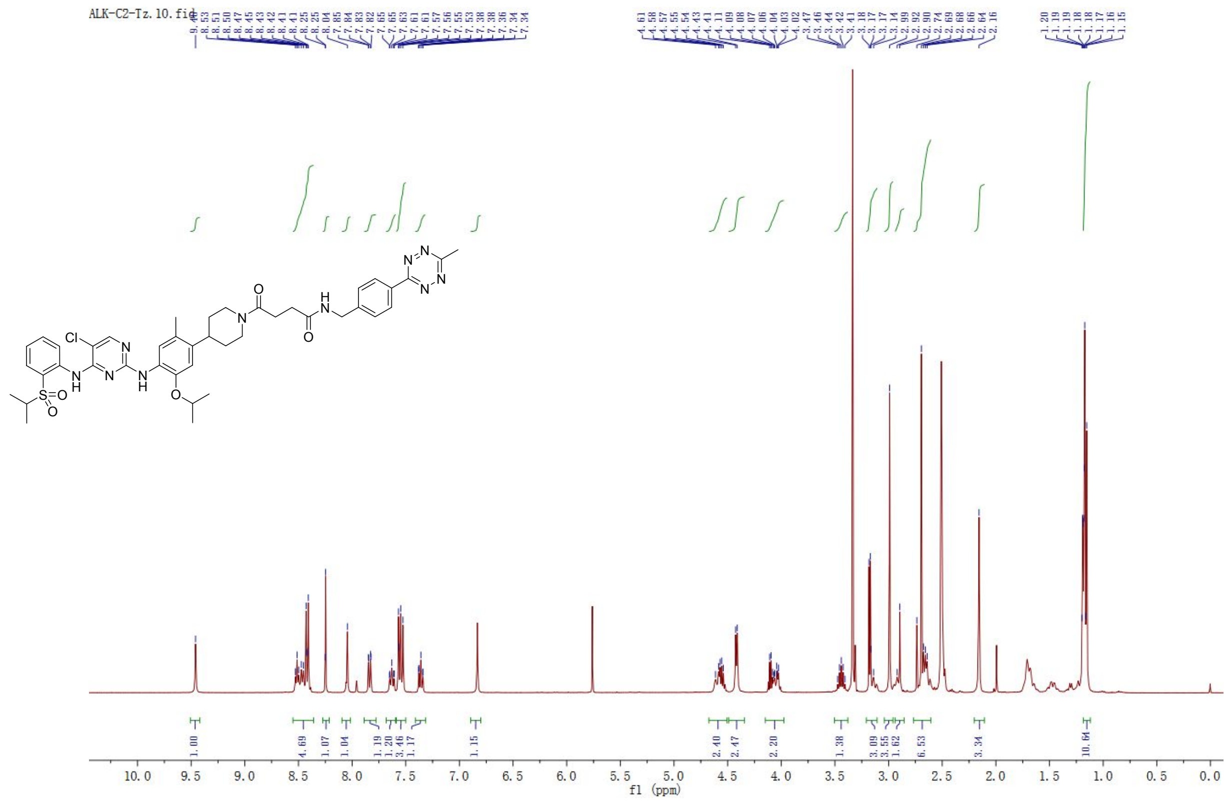

^{1}H NMR spectrum of ALK-C2-Tz

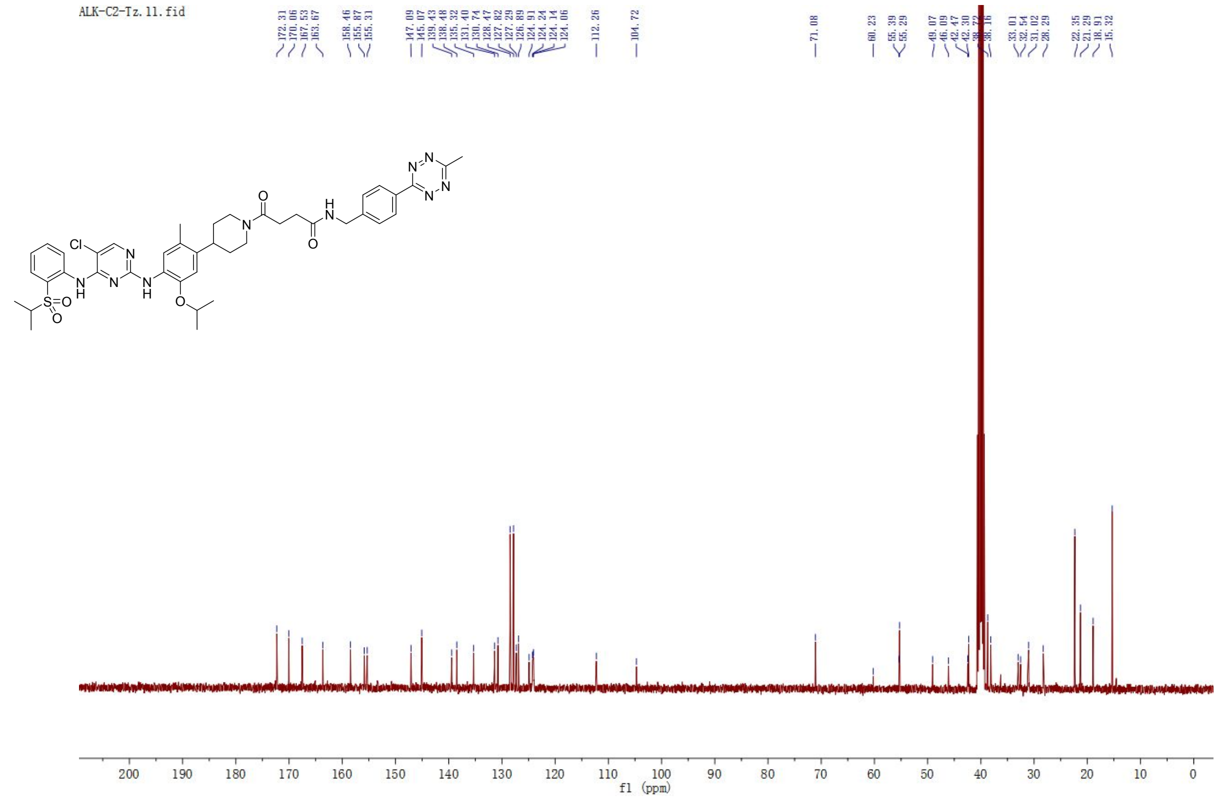

^{13}C NMR spectrum of ALK-C2-Tz

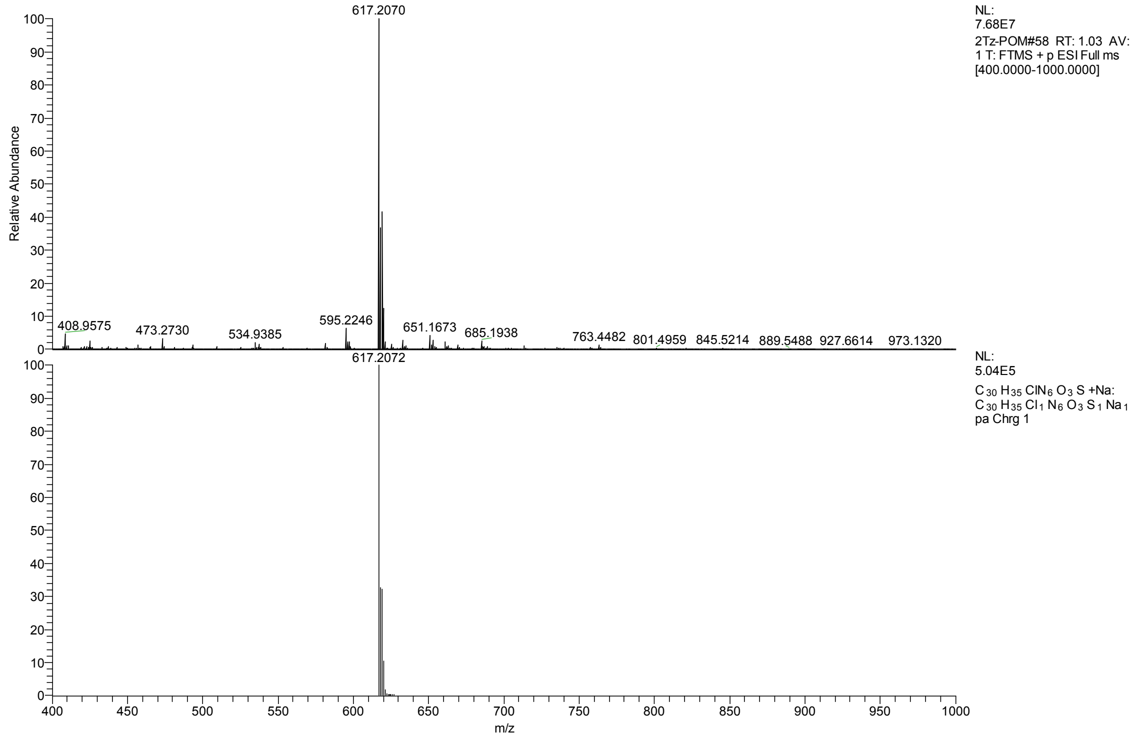

HRMS spectrum of JQ1-TCO

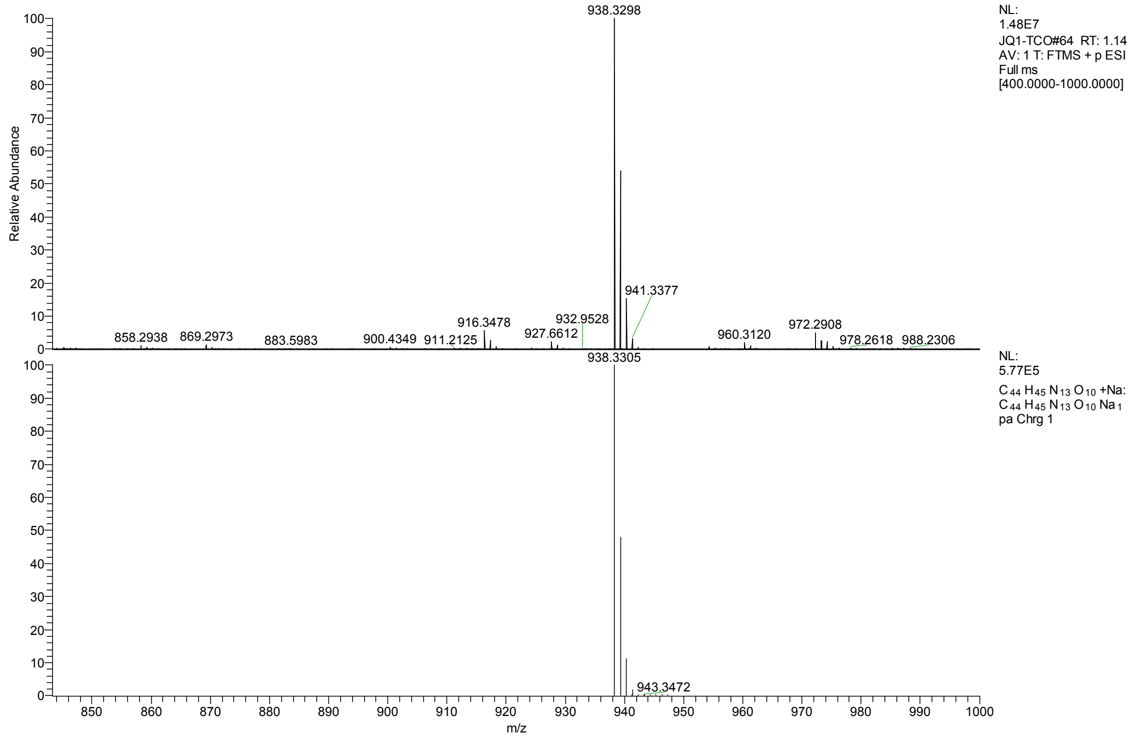

HRMS spectrum of POM-dTz

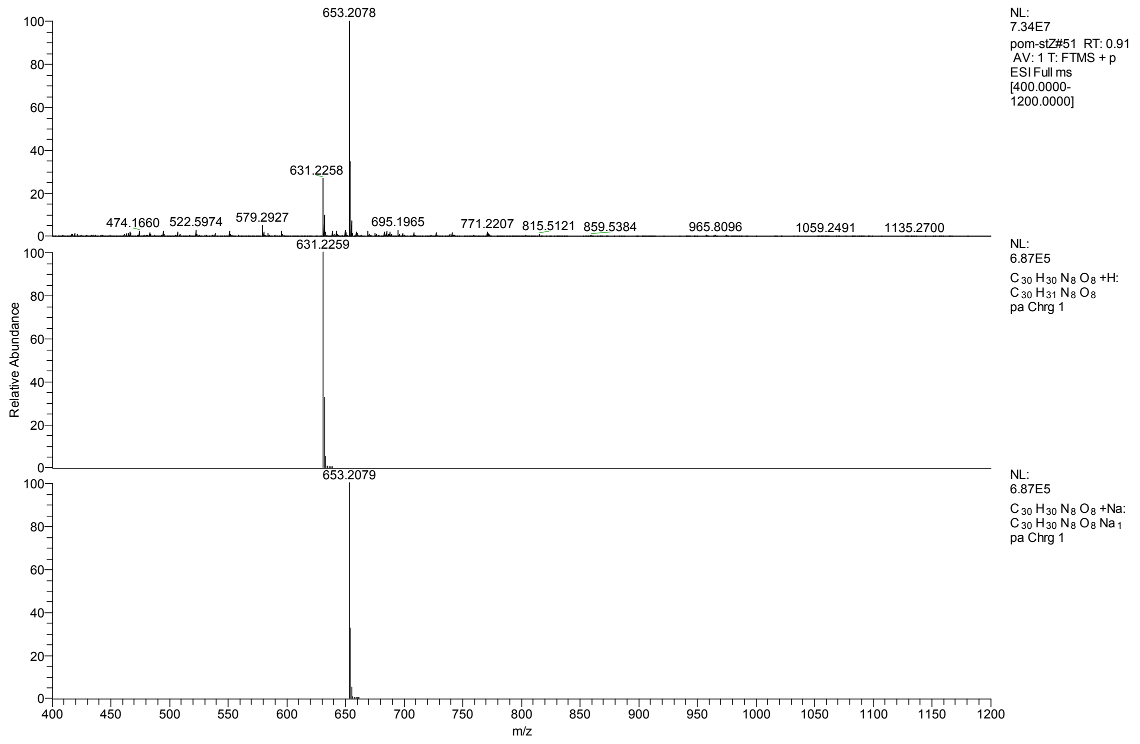

HRMS spectrum of POM-STz

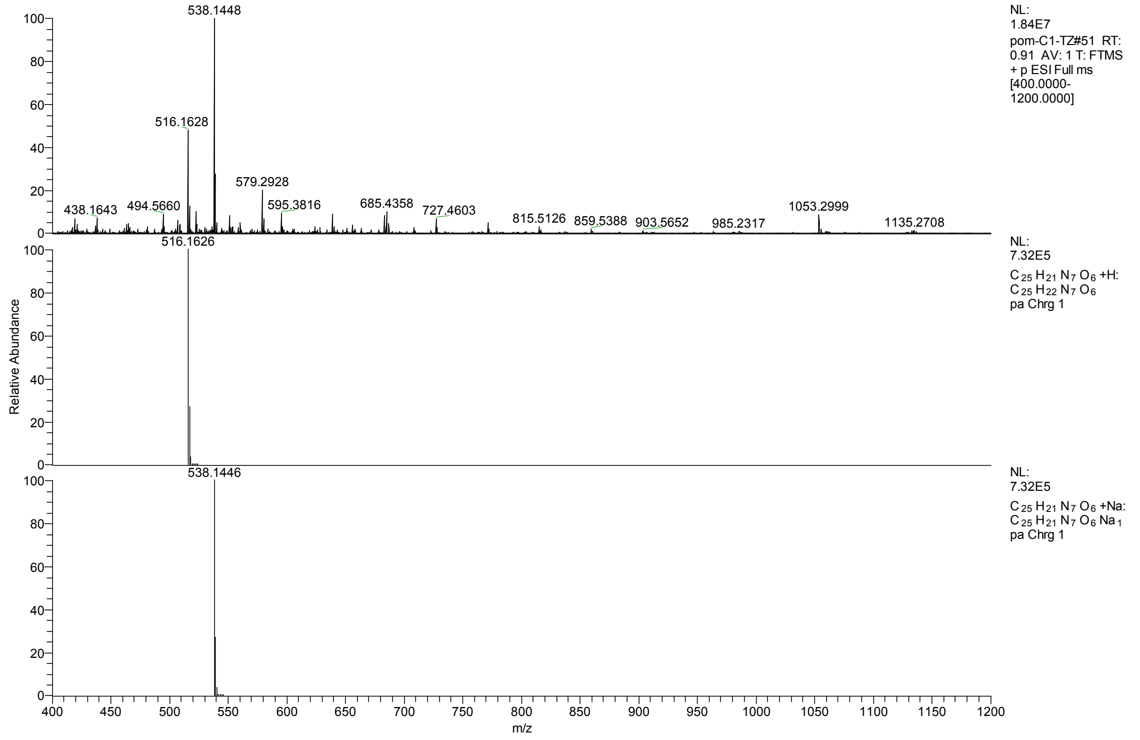

HRMS spectrum of POM-C1-Tz

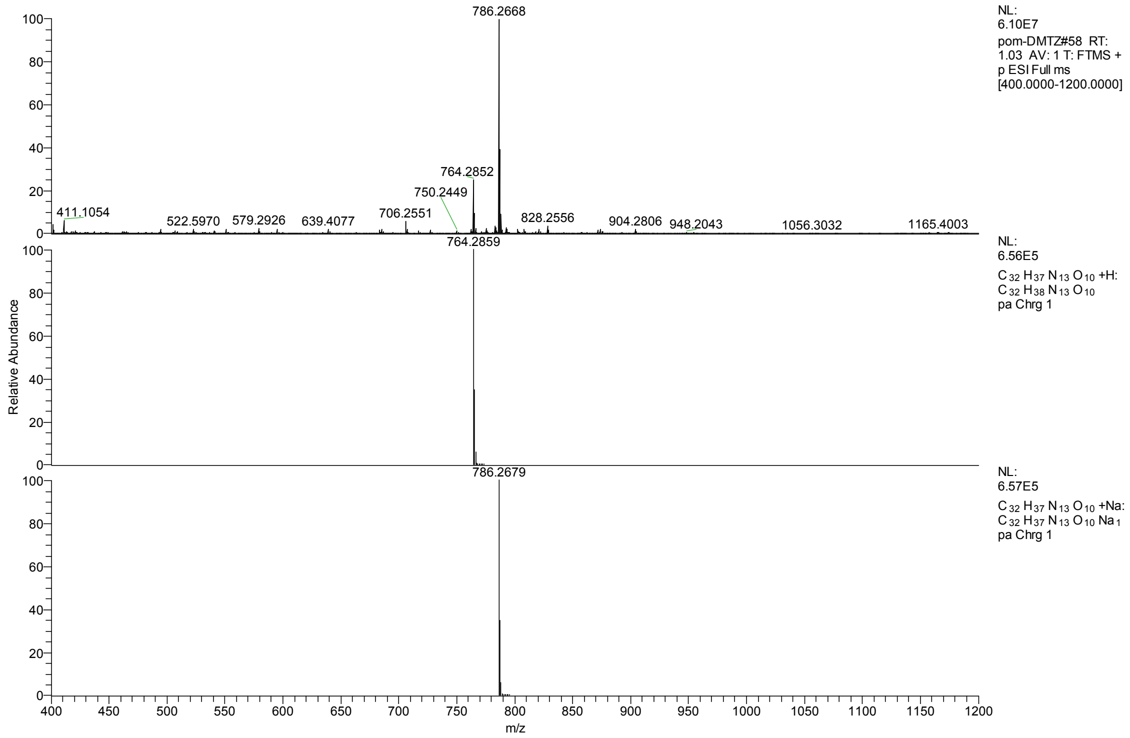

HRMS spectrum of POM-dmTz

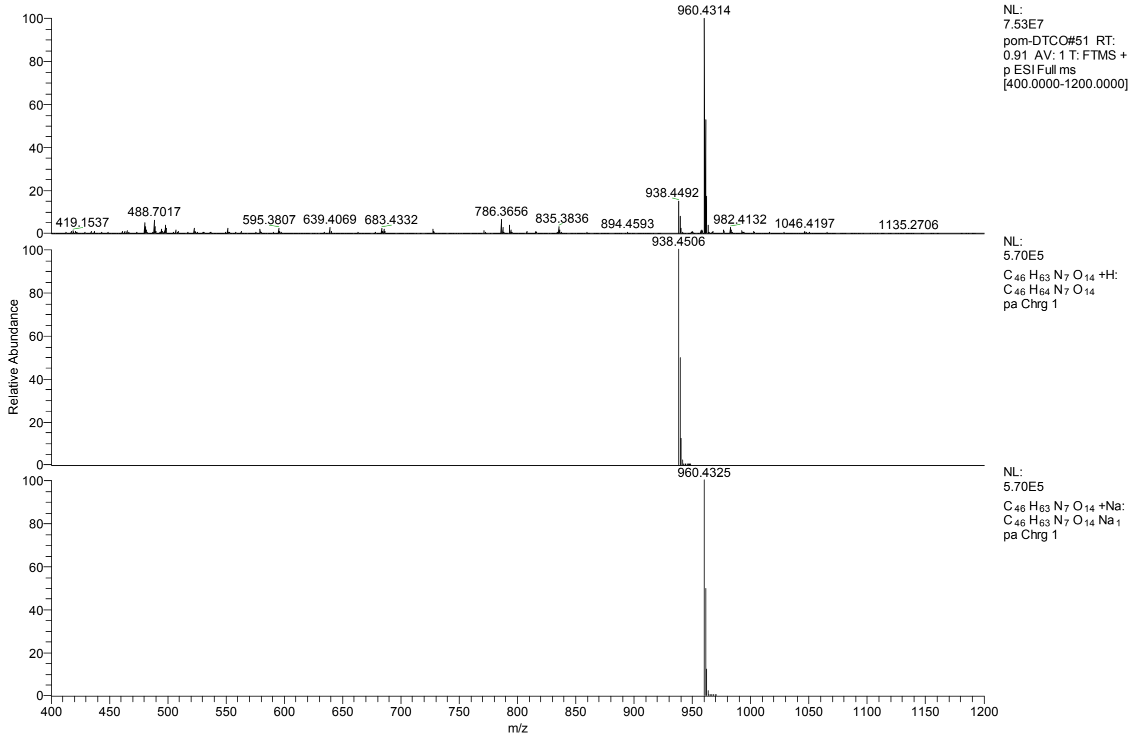

HRMS spectrum of POM-dTCO

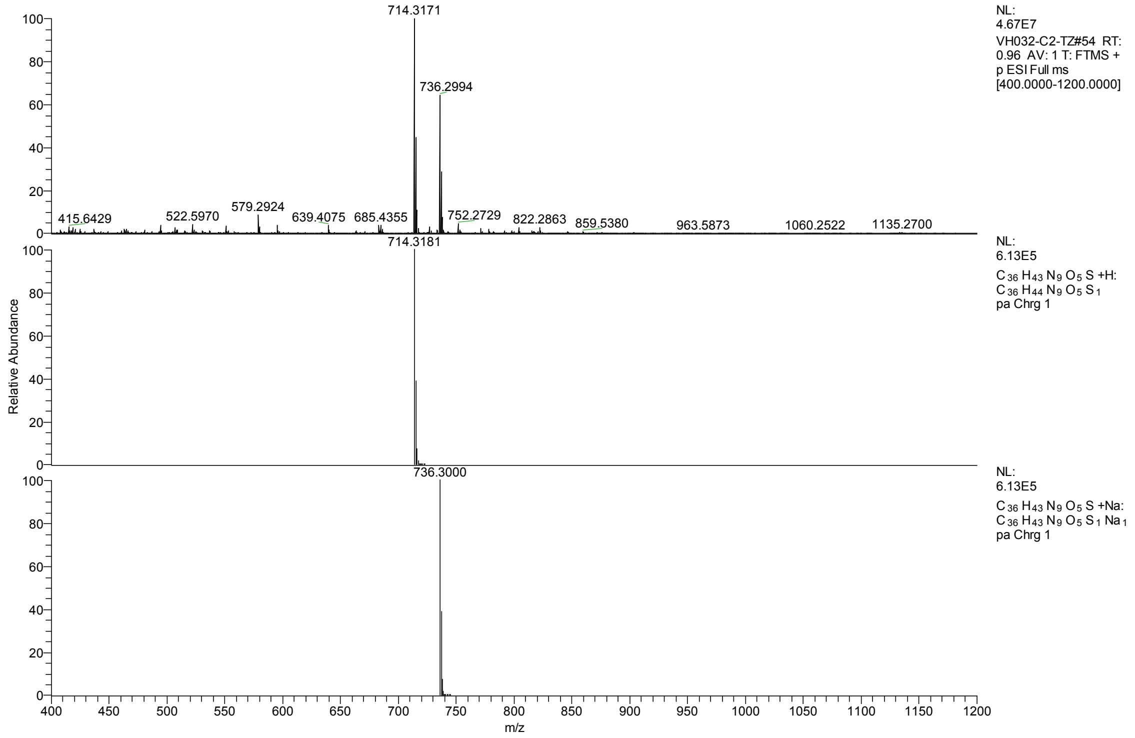

HRMS spectrum of VH032-C2-Tz

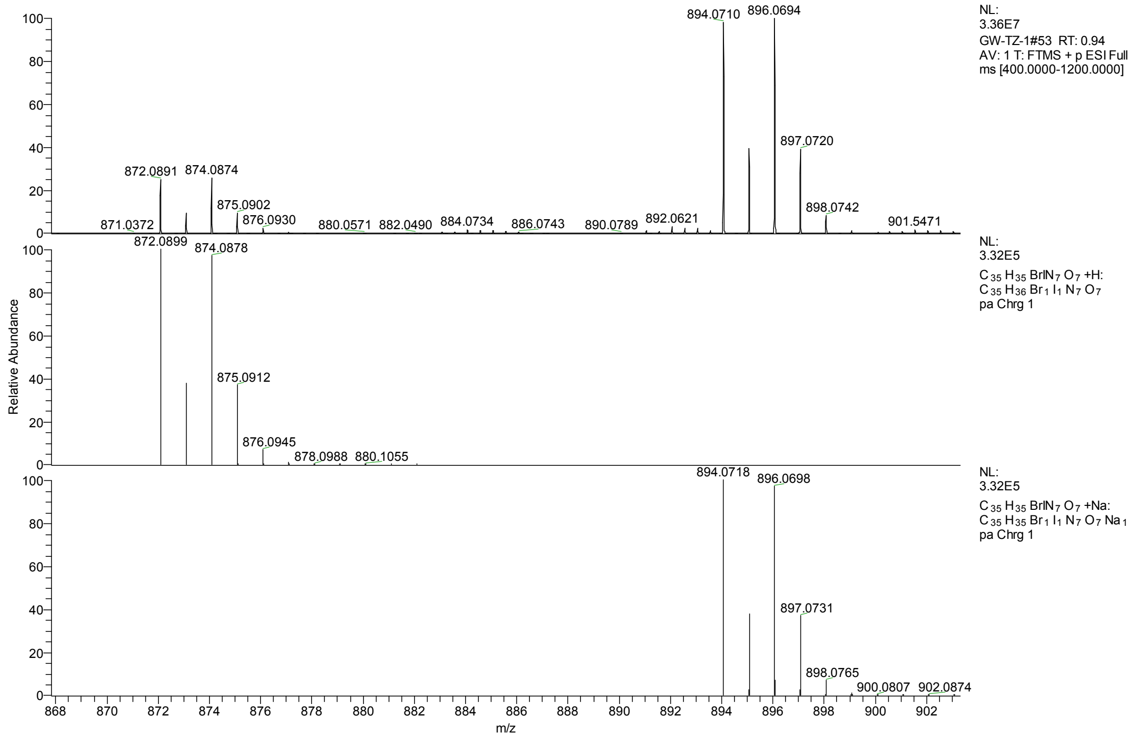

HRMS spectrum of GW-Tz-1

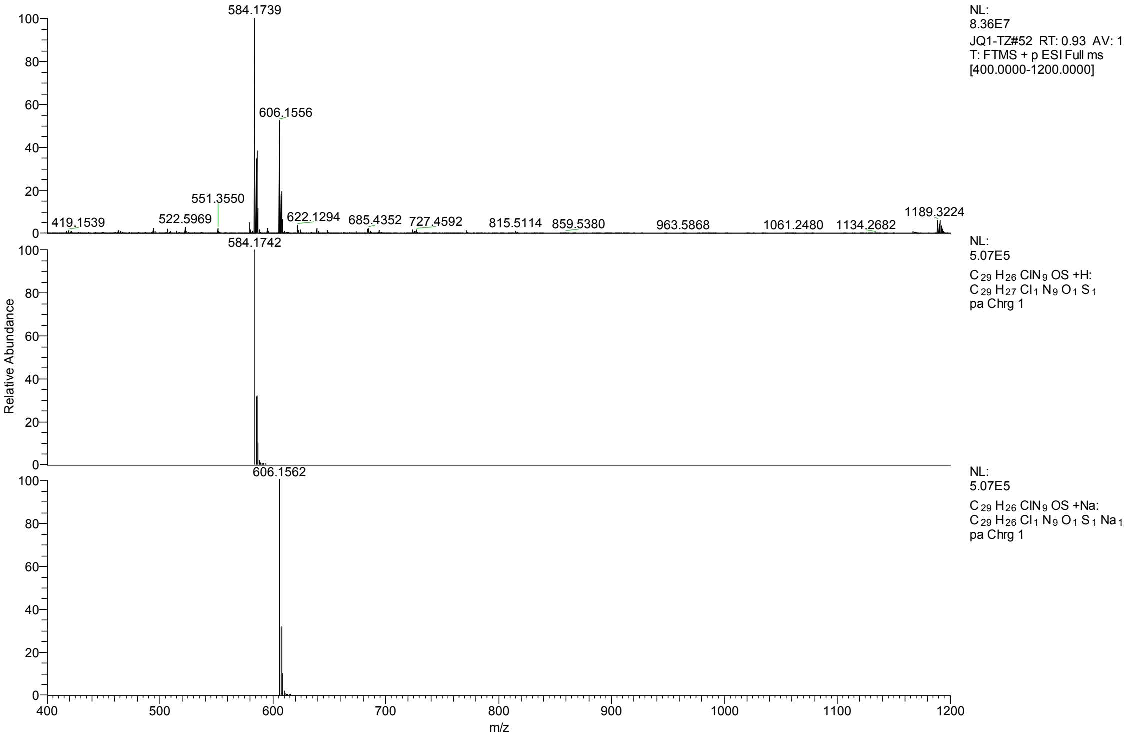

HRMS spectrum of JQ1-Tz

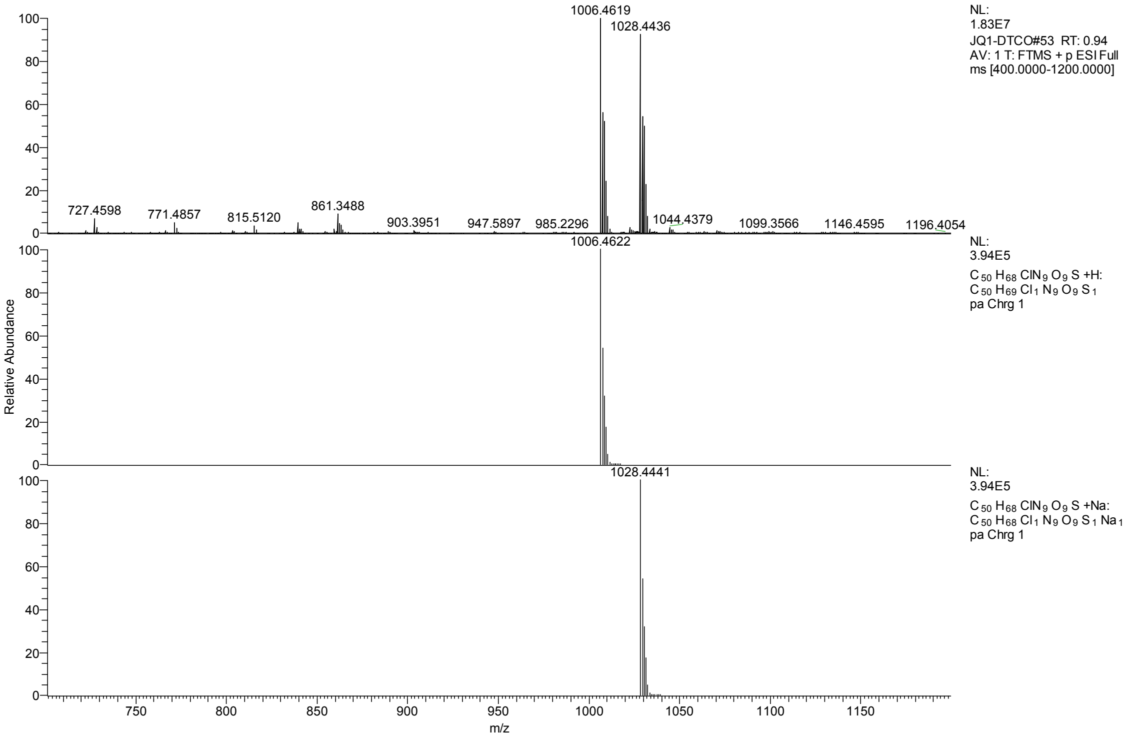

HRMS spectrum of JQ1-dTCO

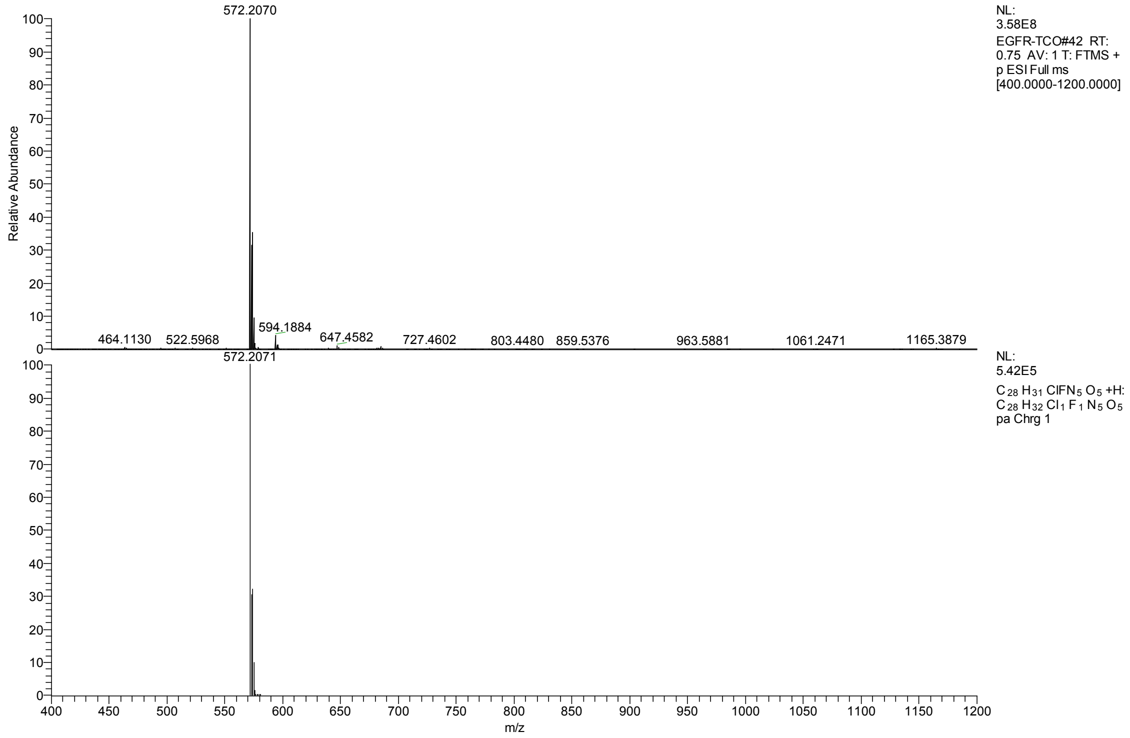

HRMS spectrum of EGFR-TCO

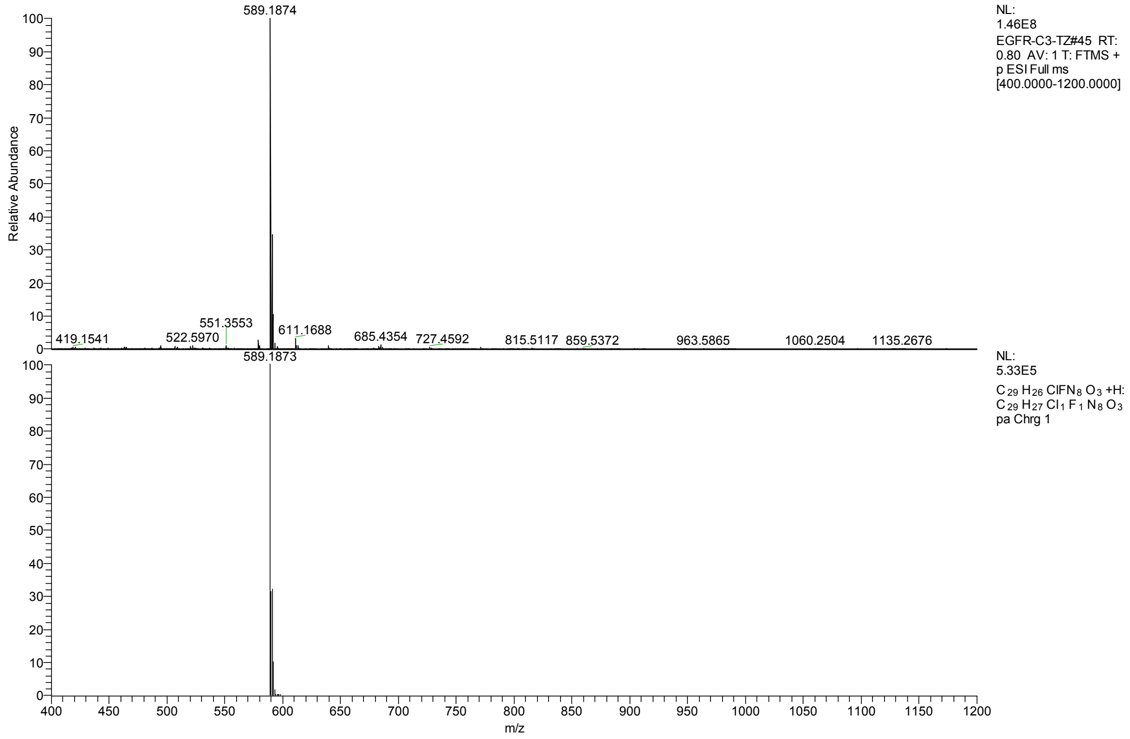

HRMS spectrum of EGFR-C3-TCO

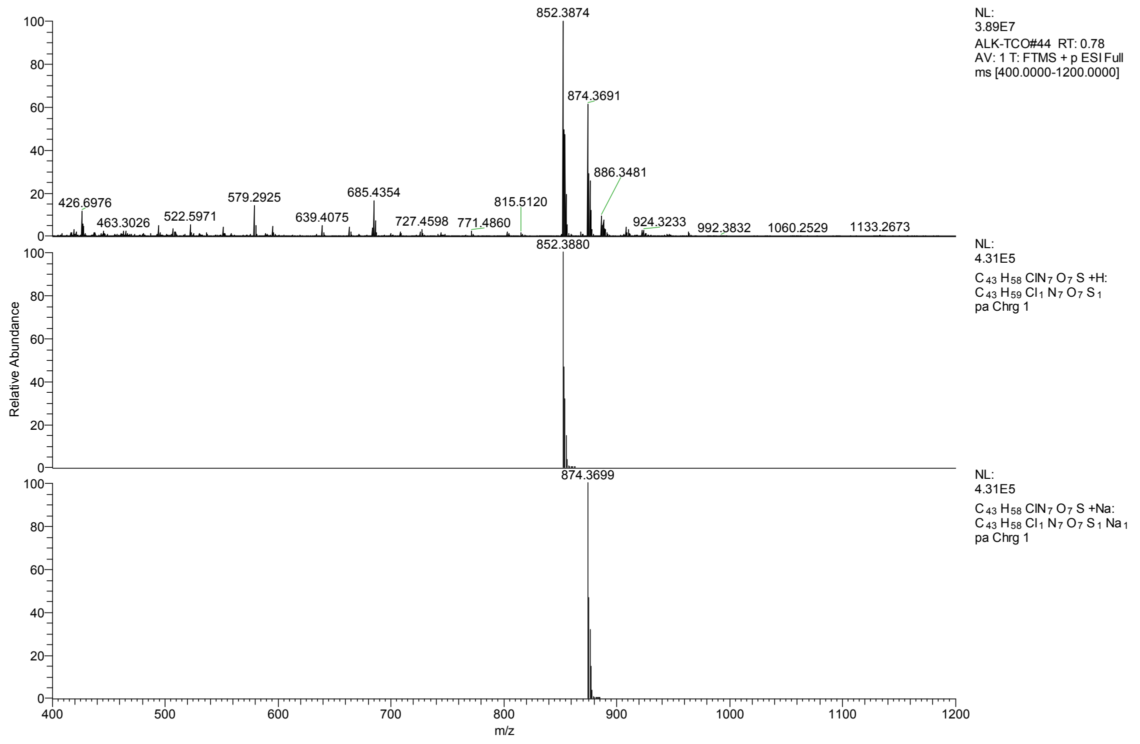

HRMS spectrum of ALK-TCO

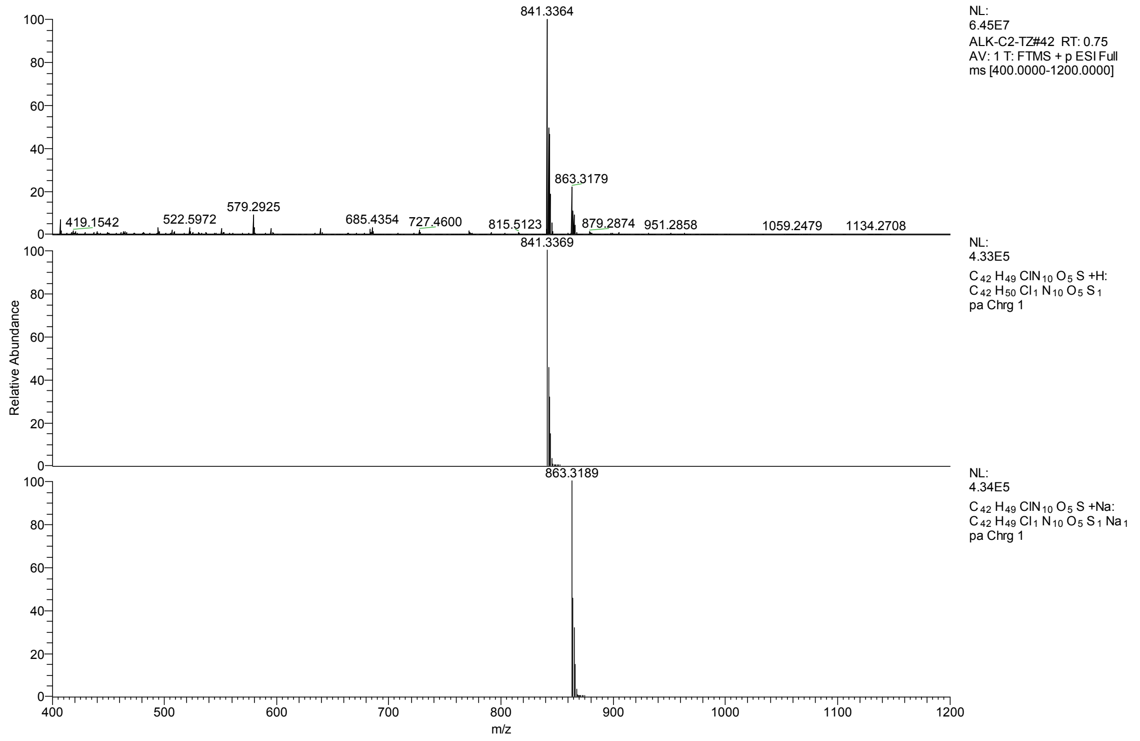

HRMS spectrum of ALK-C2-Tz

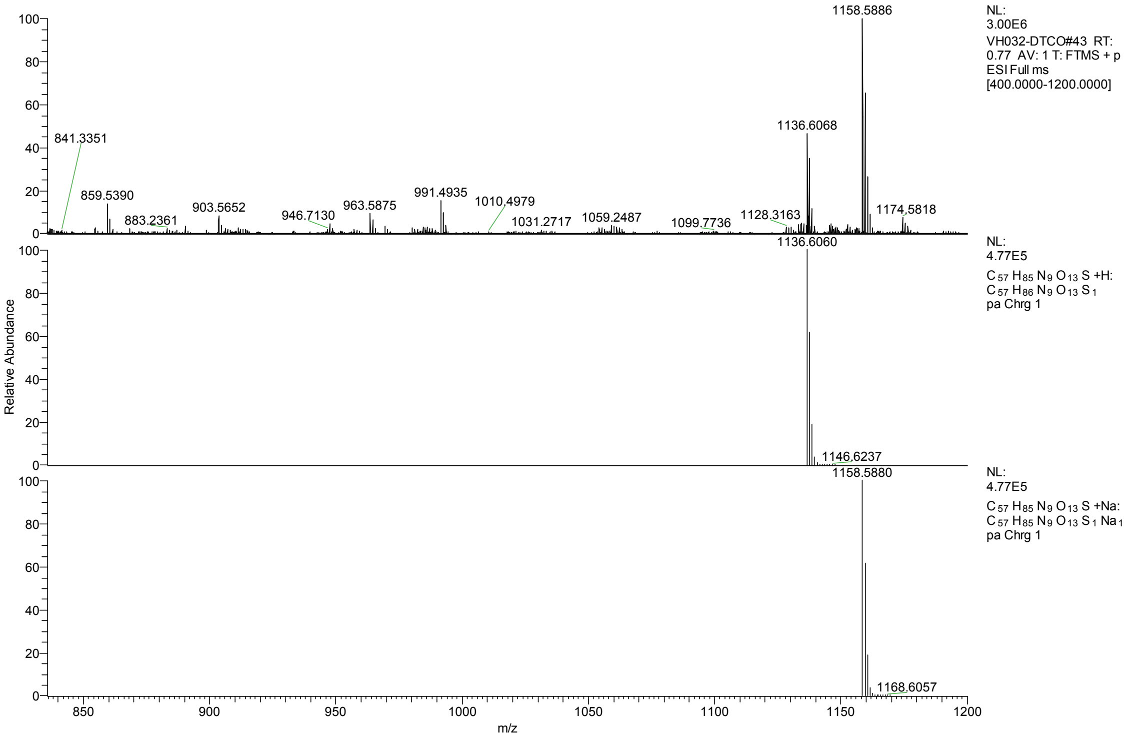

HRMS spectrum of VH032-dTCO

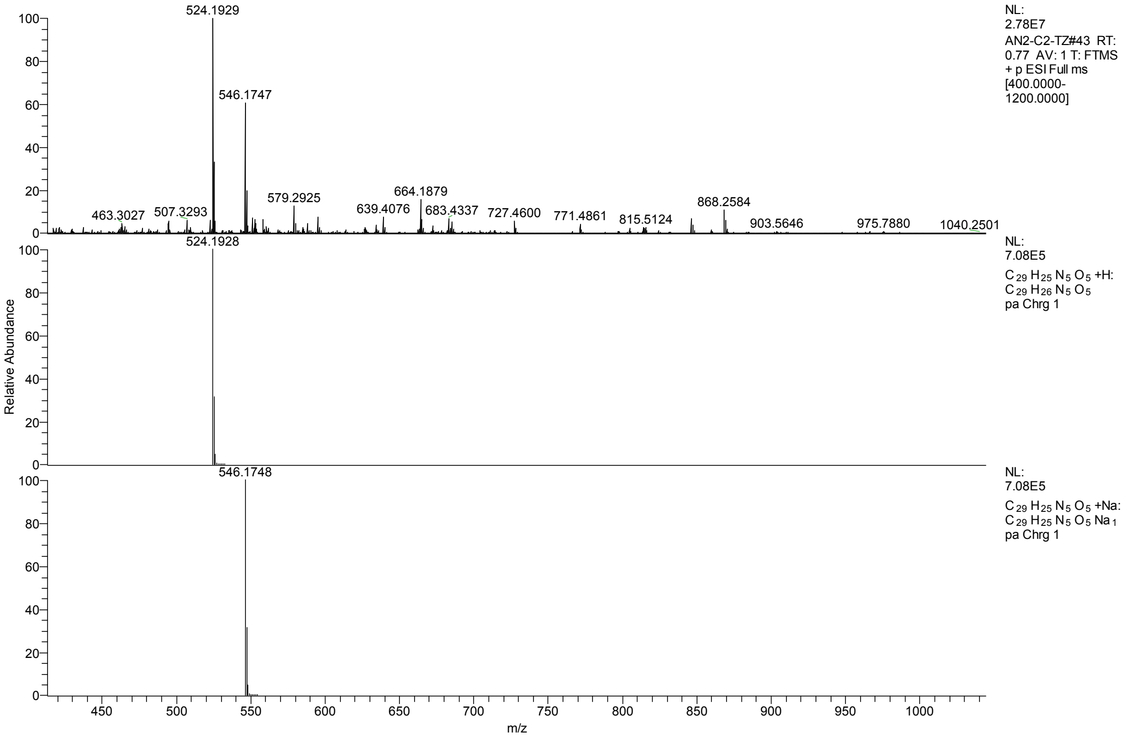

HRMS spectrum of AN2-C3-Tz

Appendix C HPLC traces of synthesized compounds

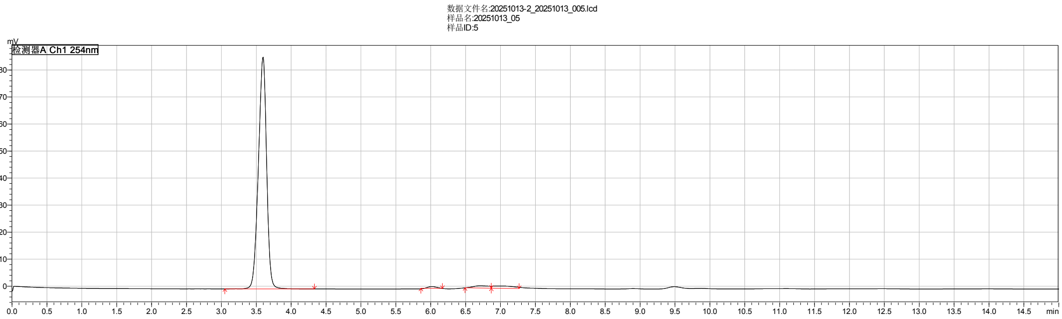

Peak No.	R. Time	Aera	Height	Aera/%	S/N
1	3.597	702968	85775	95.105	555.25
2	5.938	5355	185	0.725	1.20
3	6.711	13232	834	1.790	5.40
4	7.019	17592	836	2.380	5.41
**Total**		739146	87630	100.000	

HPLC trace of compound **EGFR-TCO**

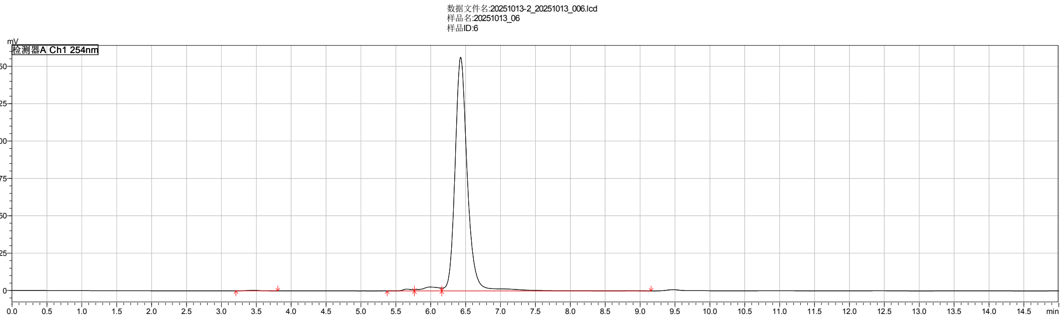

Peak No.	R. Time	Aera	Height	Aera/%	S/N
1	3.465	5018	379	0.252	4.69
2	5.658	11715	1238	0.589	15.31
3	5.992	44636	2615	2.244	32.34
4	6.428	1927563	156350	96.914	1933.47

**Total**		1988931	160582	100.000	

HPLC trace of compound **VH032-C2-Tz**

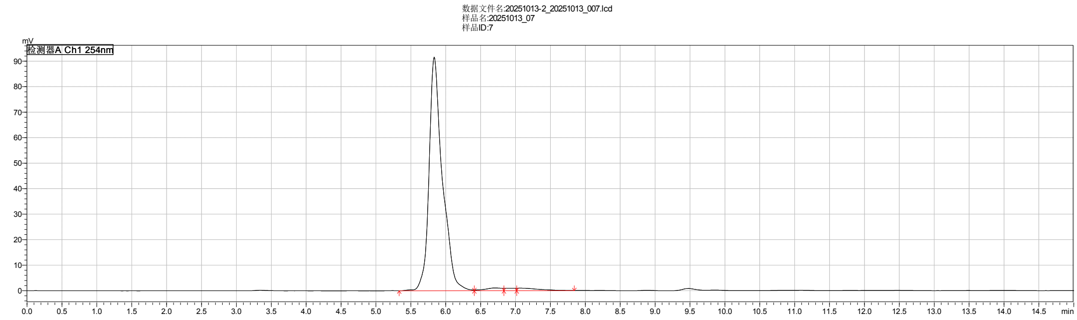

Peak No.	R. Time	Aera	Height	Aera/%	S/N
1	5.834	1221941	91645	95.742	1078.27
2	6.703	19583	1094	1.534	12.87
3	6.967	10911	1005	0.855	11.83
4	7.067	23844	1025	1.868	12.06
**Total**		1276279	94769	100.000	

HPLC trace of compound **POM-C1-Tz**

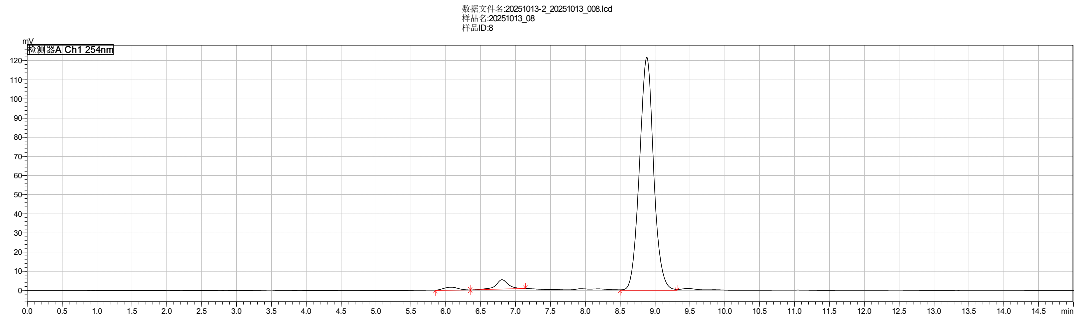

Peak No.	R. Time	Aera	Height	Aera/%	S/N
1	6.076	23029	1567	1.276	16.67
2	6.806	60138	4888	3.333	52.00
3	8.882	1721310	121864	95.391	1296.31
**Total**		1804478	128319	100.000	

HPLC trace of compound **ALK-C2-Tz**

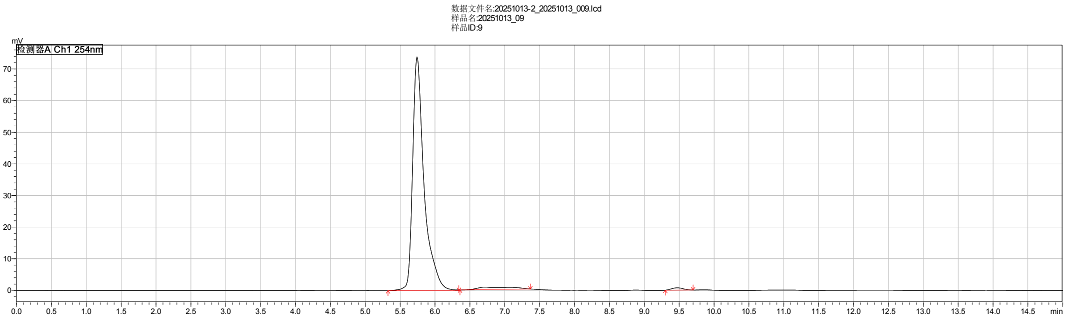

Peak No.	R. Time	Aera	Height	Aera/%	S/N
1	5.743	845760	73864	96.004	1647.65
2	7.085	26698	605	3.030	13.48
3	9.472	8509	723	0.966	16.12
**Total**		880967	75191	100.000	

HPLC trace of compound **POM-sTz**

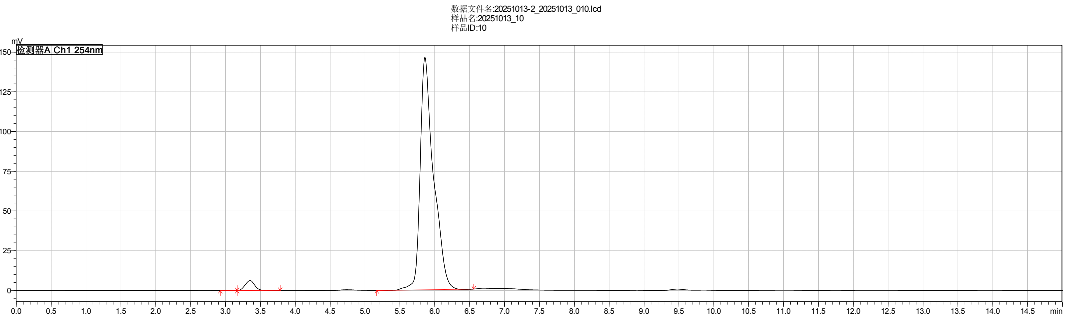

Peak No.	R. Time	Aera	Height	Aera/%	S/N
1	3.113	1141	155	0.057	1.42
2	3.352	59262	6248	2.947	56.98
3	5.859	1950241	146423	96.996	1335.30
**Total**		2010644	152826	100.000	

HPLC trace of compound **POM-dmTz**

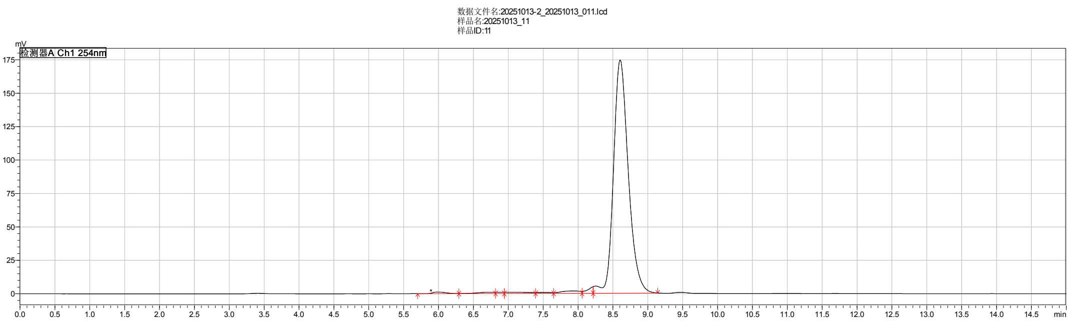

Peak No.	R. Time	Aera	Height	Aera/%	S/N
1	5.987	18103	1249	0.668	14.04
2	6.702	18039	1007	0.666	11.32
3	6.908	6923	930	0.255	10.46
4	7.100	22642	955	0.836	10.73
5	7.520	10063	754	0.371	8.48
6	7.940	32622	1704	1.204	19.16
7	8.213	26604	4704	0.982	52.89
8	8.604	2574708	174246	95.018	1959.23
Total		2709703	185549	100.000	

HPLC trace of compound **JQ1-TCO**

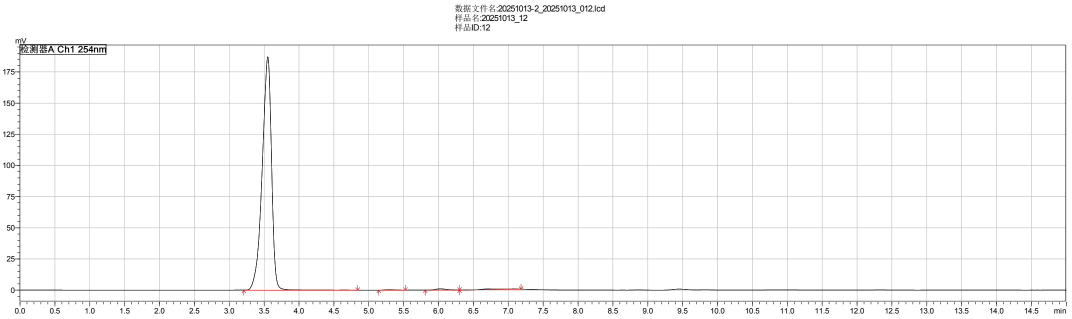

Peak No.	R. Time	Aera	Height	Aera/%	S/N
1	3.553	1745181	187177	98.611	2046.50
2	5.291	3280	403	0.185	4.40

3	6.025	11141	984	0.629	10.76
4	6.708	10155	508	0.574	5.55
**Total**		1769757	189071	100.000	

HPLC trace of compound **EGFR-C3-Tz**

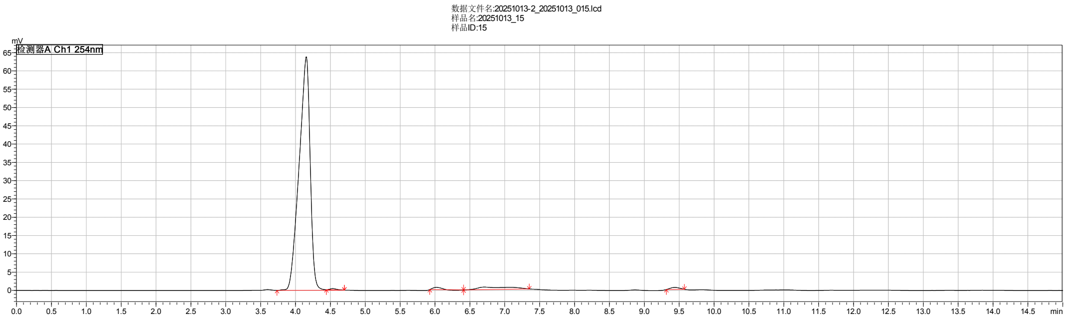

Peak No.	R. Time	Aera	Height	Aera/%	S/N
1	4.154	702983	63749	95.351	1390.33
2	4.533	2327	368	0.316	8.02
3	6.023	4245	622	0.576	13.56
4	6.707	22686	703	3.077	15.32
5	9.438	5015	547	0.680	11.94
**Total**		737257	65988	100.000	

HPLC trace of compound **POM-dTz**

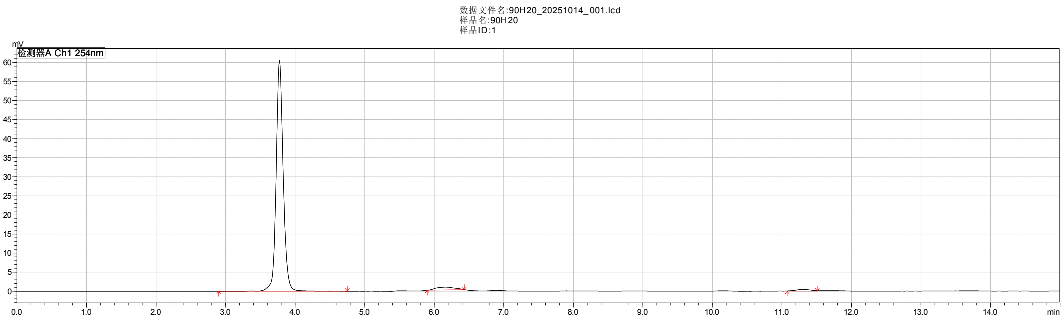

Peak No.	R. Time	Aera	Height	Aera/%	S/N

1	3.775	427887	60593	95.409	1679.16
2	6.165	13706	727	3.056	20.16
3	11.300	6886	457	1.535	12.66
**Total**		448478	61777	100.000	

HPLC trace of compound **JQ1-Tz**

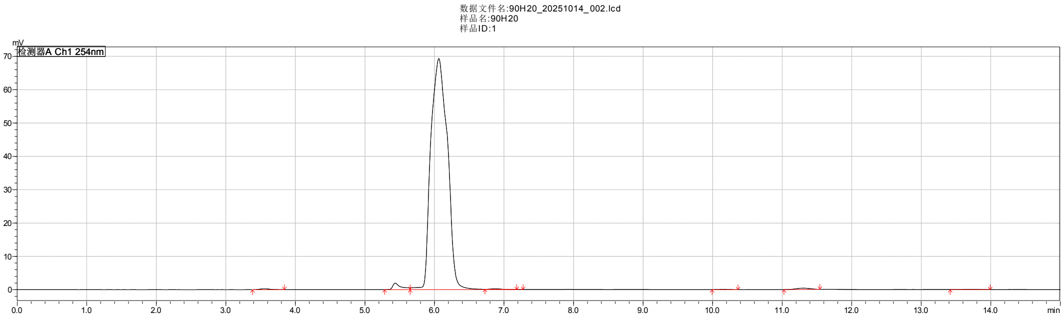

Peak No.	R. Time	Aera	Height	Aera/%	S/N
1	3.556	2938	287	0.249	14.53
2	5.436	16341	1950	1.387	98.65
3	6.063	1149839	69302	97.561	3505.19
4	6.875	1710	174	0.145	8.79
5	10.129	1136	82	0.096	4.15
6	11.305	5419	390	0.460	19.70
7	13.768	1207	53	0.102	2.66
**Total**		1178590	72238	100.000	

HPLC trace of compound **POM-dTCO**

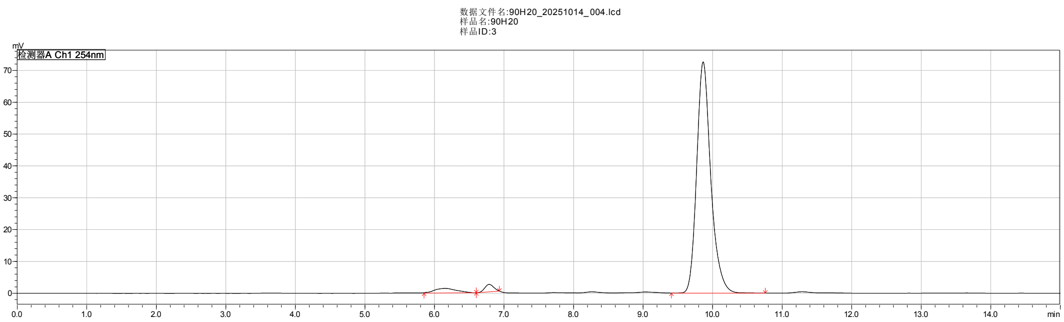

Peak No.	R. Time	Aera	Height	Aera/%	S/N
1	6.150	32391	1439	3.004	22.23
2	6.788	20961	2347	1.944	36.25
3	9.864	1024827	72653	95.052	1122.37
**Total**		1078179	76438	100.000	

HPLC trace of compound **JQ1-dTCO**

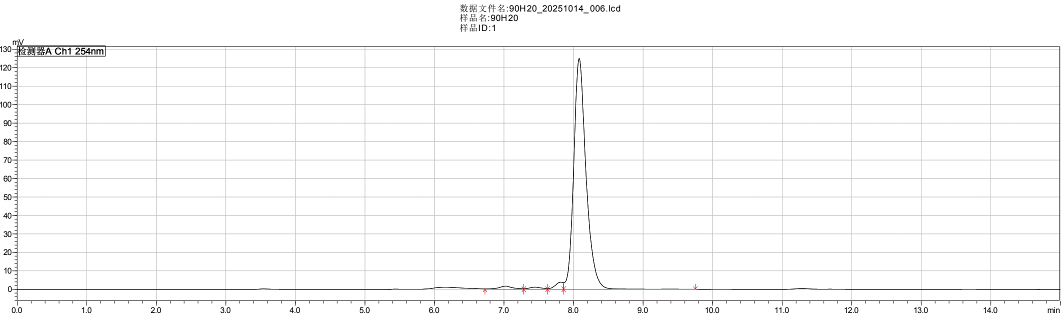

Peak No.	R. Time	Aera	Height	Aera/%	S/N
1	7.018	24982	1680	1.552	22.96
2	7.446	14566	1133	0.905	15.48
3	7.817	31512	3830	1.958	52.33
4	8.081	1538230	125060	95.584	1708.79
**Total**		1609290	131703	100.000	

HPLC trace of compound **VHO32-dTCO**

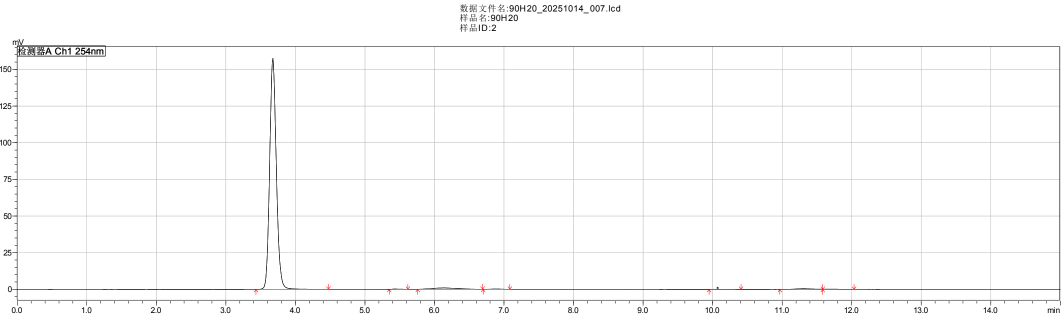

Peak No.	R. Time	Aera	Height	Aera/%	S/N
1	3.675	1051432	157598	96.699	8297.99
2	5.427	1327	220	0.122	11.56
3	6.141	21980	882	2.021	46.42
4	6.887	1805	174	0.166	9.15
5	10.168	1188	92	0.109	4.86
6	11.307	7604	464	0.699	24.43
7	11.668	1985	116	0.183	6.12
Total		1087321	159545	100.000	

HPLC trace of compound **ALK-TCO**

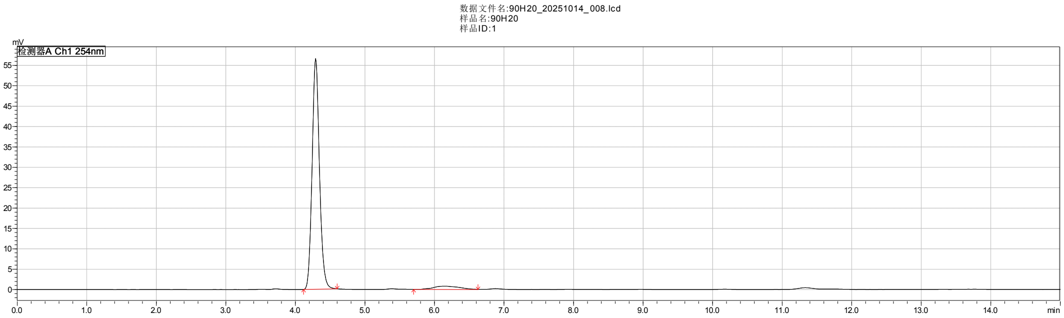

Peak No.	R. Time	Aera	Height	Aera/%	S/N
1	4.291	417074	56603	95.103	1130.34
2	6.150	21477	815	4.897	16.28
Total		438550	57418	100.000	

HPLC trace of compound **AN2-C3-Tz**
